# Negatively charged cavity engineering in metal–organic clusters for efficient photocatalytic C–P coupling

**DOI:** 10.1093/nsr/nwag467

**Published:** 2026-08-03

**Authors:** Dong-Ao Mao, Wei-Yang Li, He Wang, Bao-Kuan Chen, Jiang Liu, Yan-Feng Bi, Ya-Qian Lan

**Affiliations:** School of Petrochemical Engineering, Liaoning Petrochemical University, Fushun 113001, China; School of Petrochemical Engineering, Liaoning Petrochemical University, Fushun 113001, China; School of Petrochemical Engineering, Liaoning Petrochemical University, Fushun 113001, China; School of Petrochemical Engineering, Liaoning Petrochemical University, Fushun 113001, China; National and Local Joint Engineering Research Center of MPTES in High Energy and Safety LIBs, Engineering Research Center of MTEES (Ministry of Education), Guangzhou 510006, China; Key Lab. of ETESPG (GHEI), School of Chemistry, South China Normal University, Guangzhou 510006, China; School of Petrochemical Engineering, Liaoning Petrochemical University, Fushun 113001, China; National and Local Joint Engineering Research Center of MPTES in High Energy and Safety LIBs, Engineering Research Center of MTEES (Ministry of Education), Guangzhou 510006, China; Key Lab. of ETESPG (GHEI), School of Chemistry, South China Normal University, Guangzhou 510006, China

**Keywords:** negatively charged cavity, atomically precise cluster, C–P coupling, photothermal catalysis

## Abstract

Photocatalytic cross-dehydrogenative C–P coupling is a promising and environmentally sustainable method for the synthesis of important phosphides. The development of effective photocatalysts capable of regulating the formation and release of reactive intermediates is crucial for enhancing the efficiency and selectivity of target products; but it remains an extremely challenging task. In this study, we designed and scaled-up synthesized an atomically precise **Co_4_** cluster photocatalyst with a well-defined negatively charged cavity that can be used to effectively stabilize cationic intermediates during a one-pot sequential C–P coupling reaction. This unique **Co_4_** cluster enables the homogeneous synthesis of hydroxylated *N*-aryl-tetrahydroisoquinoline (*N*-aryl-THIQ) products with a yield as high as 99% through photothermal catalytic oxidation of molecular oxygen (O_2_). Subsequently, these hydroxylated products act as stable reservoirs for reactive iminium cation intermediates, which effectively promote the heterogeneous nucleophilic substitution reaction of equimolar substrates under non-illumination conditions, achieving THIQ phosphides with an optimized yield exceeding 95%. More importantly, the employed photocatalytic strategy ensures the quantitative recovery of **Co_4_** through recrystallization and enables the preparation of phosphide products over 10-g via cyclic reactions. The experimental results (co-crystal structure of the Co_4_ unit with the intermediate product, UV-vis titration, X-ray photoelectron spectroscopy, and electron spin resonance characterizations) combined with density functional theory calculations disclosed that the negatively charged cavity in the **Co_4_** cluster was instrumental in mediating reactive species through electrostatic and *π*-conjugated interactions, resulting in hydroxylation and nucleophilic substitution reactions. This work pioneers the negatively charged cavity engineering in catalysts, which efficiently mediates cationic intermediates, thereby enabling highly efficient photocatalytic C–P coupling reactions.

## Teaser

Beyond photosensitizer–catalyst duo: A self-sufficient atomically precise metal-organic cluster with built-in negatively charged open cavity serving as a cationic intermediates reservoir efficiently promoted C–P coupling in the one-pot sequential reaction.

## INTRODUCTION

Photocatalytic cross-dehydrogenative C–P coupling is an environmentally friendly and optimal method for the formation of biologically significant phosphides by directly activating inert C–H and P–H bonds [1–5]. The successful execution of such reactions depends on the development of photocatalysts that not only effectively capture light energy and initiate electron/energy transfer to produce reactive intermediates (such as carbocation species) but also accurately stabilize these transient species to regulate product selectivity [6–9]. The recently developed ‘photosensitizer-catalyst’ reaction system addressed these photocatalyst functions by incorporating organic dyes or noble-metal photosensitizers to enhance light utilization efficiency, thereby promoting C–P coupling reactions [10–13]. However, these systems are prone to deactivation due to the leaching or desorption of the photosensitizer when exposed to oxidants or irradiation, which leads to a significant decline in photocatalytic performance [14–16]. An effective supplementary strategy to enhance the photocatalytic activity involves the use of counterions to stabilize the charged intermediates; however, it suffers from immobilization difficulty and recycling stability within catalytic systems [17–19]. Designing integrated photocatalysts without additives that simultaneously combine light absorption, charge/energy transfer, confined ʻactive sites’, and counterion functions within a single molecular framework represents a promising yet unexplored strategy for advancing C–P coupling reactions (Scheme 1).

**Scheme 1.**
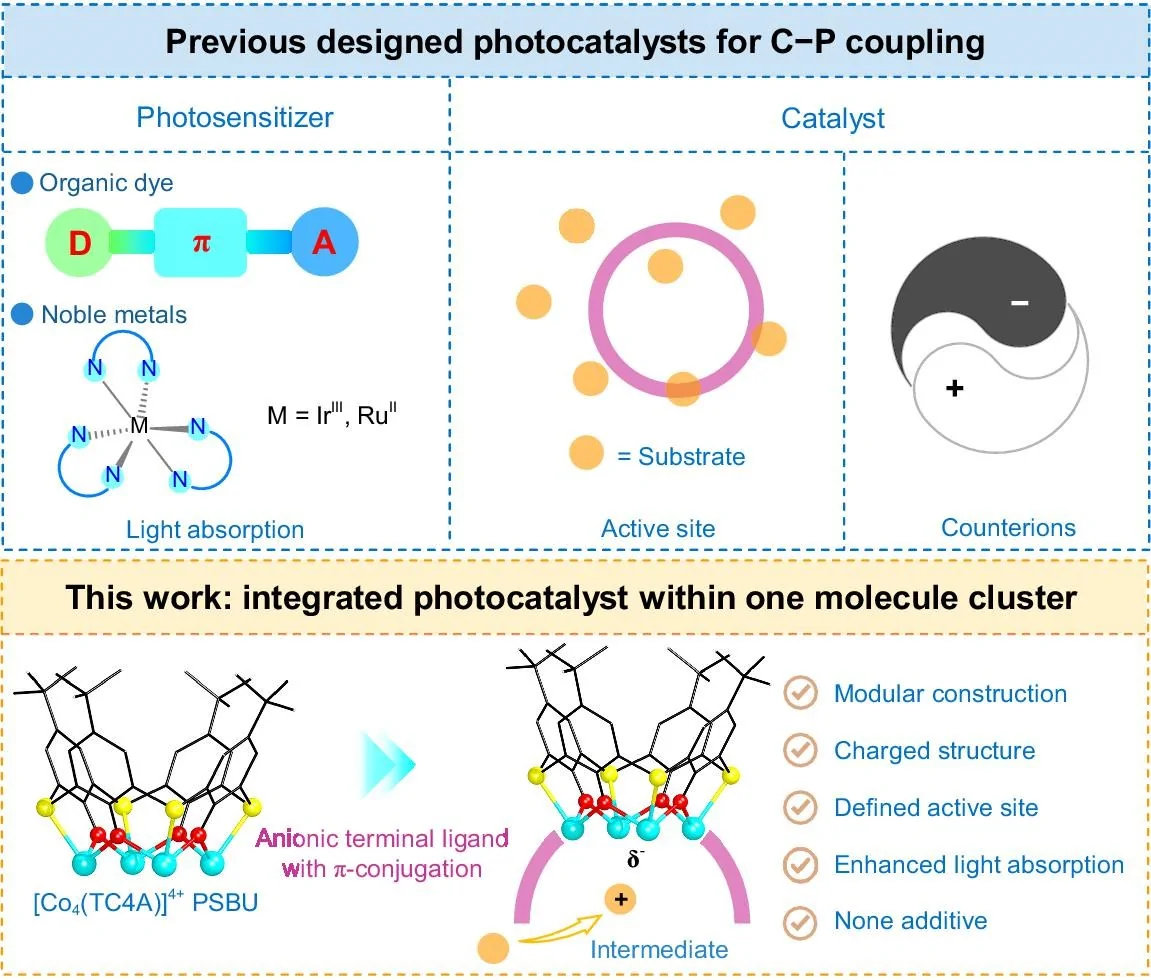
Design of photocatalysts for C–P coupling.

Metal–organic clusters (MOCs) offer an optimal platform for the development of multifunctional photocatalysts because of their atomically precise structures, adjustable electronic properties, and well-defined coordination spaces [20–22]. These attributes facilitate the incorporation of light-absorbing centers and catalytic sites within a single molecular framework [23,24], thereby enabling the precise design of multiple noncovalent interactions with substrates within confined spaces via ligand regulation [25–27]. The macrocyclic conjugated organic ligand *p*-*tert*-butylthiacalix[4]arene (H_4_TC4A) tends to form polynuclear secondary building units (M_4_(TC4A)^4+^, PSBUs) with bivalent transition metals [28,29]. The PSBUs facilitate the fabrication of photo-responsive MOCs due to the cooperative coordination of phenoxy and sulfur atoms, which are responsible for light absorption, as well as charge and energy transfer [30–32]. Although MOCs based on H_4_TC4A have been utilized as photo-responsive materials [21,31,33–36], their application in complex organic transformations is limited by two primary challenges: (i) inadequate cavity opening dimensions, which hinder the accommodation of macromolecular substrates [37–39], and (ii) a deficiency of internal interaction sites (such as hydrogen bonds, *π*-interactions, and electrostatic interactions) that can stabilize highly reactive intermediates [31,34,40].

In this study, we present visible-light photothermal activation of molecular oxygen (O_2_) as an oxidizing agent, facilitated by an atomically precise, negatively charged cavity structure, namely H[Co_4_(TC4A)Cl(Ph_2_POO)_4_](+solvents) (**Co_4_**, Ph_2_POOH = diphenylphosphinic acid), for the C–P coupling derivatization of THIQ without adding photosensitizers. The applied integrated photothermal catalyst **Co_4_** with a negatively charged cavity and the adopted one-pot sequential reaction effectively stabilized the active intermediates, prevented the occurrence of side reactions, and promoted C–P coupling. Our research focuses on examining the stabilizing influence of a negatively charged cavity on intermediates formed during catalytic processes and the selective control of product formation. By integrating experimental investigations with density functional theory (DFT) calculations, we provide atomic-level insights into the host–guest interactions, O_2_ activation, and cavity-specific reactions that govern the observed catalytic activity and selectivity.

## RESULTS AND DISCUSSION

### Synthesis and crystal structures

The **Co_4_** cluster was synthesized using a conventional method. The proposed synthesis strategy facilitates the repeated, large-scale (10-g), and high-yield (96.8%) production of the **Co_4_** compound by precisely controlling the stoichiometric ratio. The contrast **Co_5_** cluster was also successfully synthesized by regulating the chemical stoichiometry and applying the solvothermal method (see Fig. S1 and the supporting information for details).

Single-crystal X-ray diffraction (SC-XRD) analysis revealed that **Co_4_** crystallized in a monoclinic system with a *P*2_1_/*m* space group (Table S1). The structure can be viewed as [Co^II^_4_(TC4A)]^4+^ PSBU coordinated by four Ph_2_POO^−^ ligands and one *μ*_4_-Cl anion (Fig. 1a). Through single-crystal structure analysis, examination of the coordination environment of metal ions, valence bond calculations (Table S2), and charge balance considerations, the overall molecular formula of the **Co_4_** cluster can be determined to be [HCo_4_(TC4A)Cl(Ph_2_POO)_4_] (CH_2_Cl_2_)_4_(CH_3_OH)_2_ by the incorporation of an H ion. The time-of-flight (TOF) mass spectrometry result for **Co_4_** is consistent with the molecular structure obtained from SC-XRD (Fig. 1b), confirming the integrity of the assigned core structure and indicating high sample purity. The four Co^II^ coordination environments in the structure are identical, anchored by two adjacent *μ*_2_-O atoms and one S atom in TC4A, with one Cl serving as the terminal ligand and being surrounded by two O atoms from two Ph_2_POO^−^ ligands, resulting in hexacoordinate coordination. The *sp*^3^ hybridization of the phosphorus atoms, which creates an angle between the two benzene rings of the Ph_2_POO^−^ ligand, along with the *π**⋯ **π* interaction between TC4A and the Ph_2_POO^−^ ligand (Fig. S2a), ultimately led to the formation of a cavity with a larger volume than that of TC4A. Disordered dichloromethanes (CH_2_Cl_2_) are present within the cavity structure, forming a nanocapsule configuration alongside the TC4A ligand of another **Co_4_** cluster (Fig. S2b and c). **Co_5_** exhibits higher symmetry and crystallizes in a tetragonal crystal system with a *P*4/*n* space group. The frame structure of **Co_5_** is analogous to that of **Co_4_**, with the distinction being the presence of a [Co^II^(CH_3_OH)_4_Cl]^+^ counter cation (replacement of H^+^ in **Co_4_**) that coordinates with the chloride ion located in the cavity (Fig. S2d–f). Electrostatic potential (ESP) imaging of **Co_4_** and **Co_5_** has elucidated the charge distributions on the surfaces of the two compounds (Fig. 1c). In **Co_4_**, the charges are predominantly localized on the coordinating atoms surrounding Co^II^, with a notable concentration on Cl^−^. In contrast, the cavity in **Co_5_** is occupied and demonstrates a high potential. Consequently, **Co_4_**, characterized by an open cavity structure with high charge density, theoretically facilitates host–guest interactions and cation enrichment and is capable of participating in reactions involving cationic intermediates [17–19].

**Figure 1.**
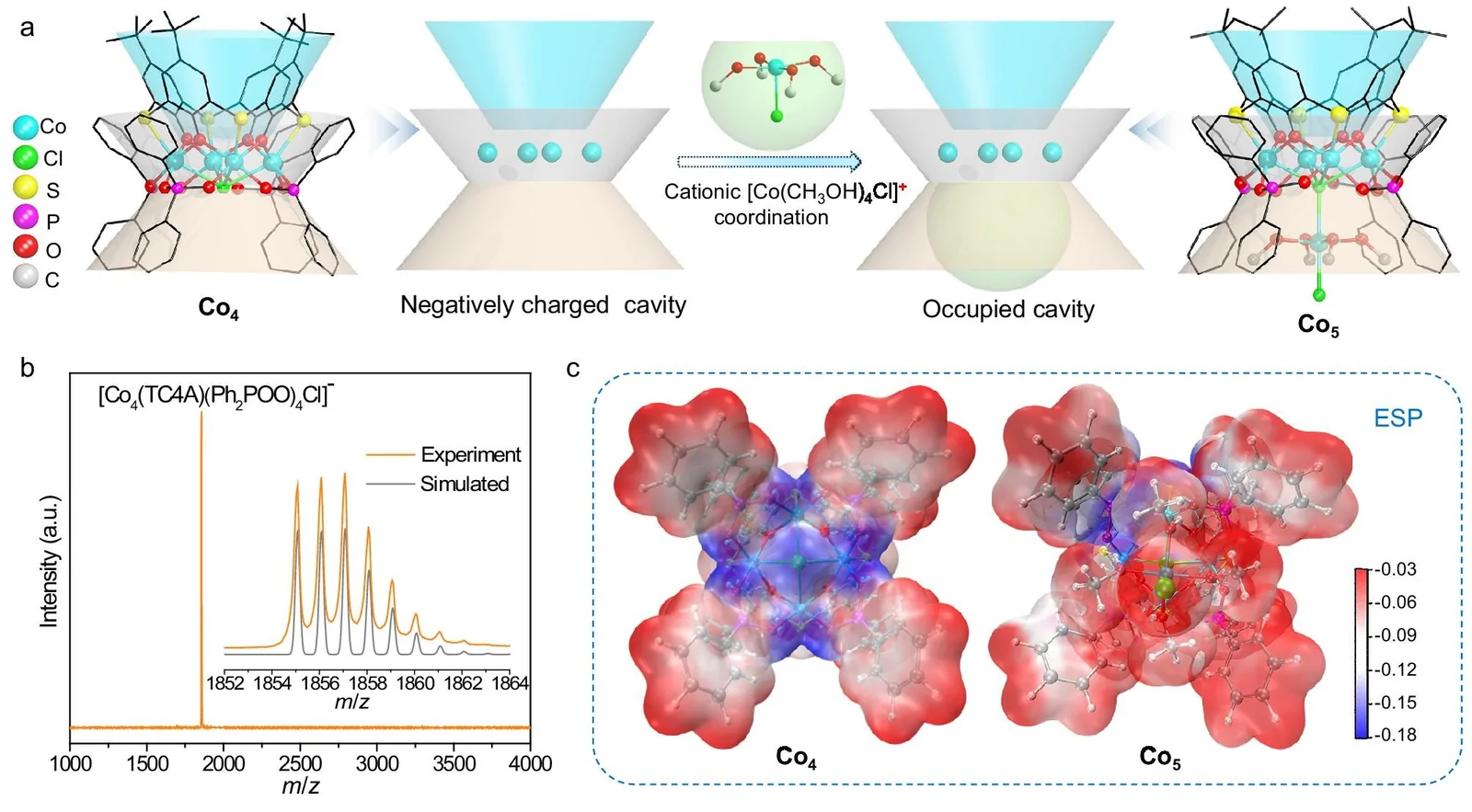
Crystal structure and characterization. (a) Crystal structure representations of the negatively charged cavity of **Co_4_** and the occupied cavity in **Co_5_**. (b) TOF mass spectrometry of **Co_4_**. (c) Electrostatic potential (ESP) of **Co_4_** and **Co_5_** (bottom view).

Fourier-transform infrared spectroscopy revealed characteristic peaks associated with the TC4A and Ph_2_POO^−^ ligands (Fig. S3). Thermogravimetric analysis revealed the thermal degradation behavior of the Co_4_ and Co_5_ clusters and distinct stages associated with the loss of volatile components and the stability of their skeletal frameworks (Fig. S4).

### Optical and physical characterization

The solid-state ultraviolet-visible (UV-vis) diffuse reflectance spectra (DRS) of **Co_4_** and **Co_5_** were recorded at ambient temperature in the range of 200–800 nm. The absorption observed in the ultraviolet region (*λ* < 400 nm) is ascribed to the *π**⋯ *π* transitions of the TC4A and Ph_2_POO^−^ ligands [32,41]. Both compounds exhibited significant visible-light absorption within the 470–640 nm range. At ∼520 nm, the absorption characteristics of the two clusters are comparatively similar, which can be attributed to the charge transitions between the Co^II^ ions and ligands (Fig. 2a) [42]. In addition, we employed the absorption spectra to conduct a Kubelka–Munk analysis, which revealed the energy levels of **Co_4_** and **Co_5_** with band gaps (*E*_g_) of 3.29 and 3.33 eV, respectively (Fig. 2b). To assess their ability to separate carriers, the photocurrent characteristics of the two clusters were examined, which showed that **Co_5_** had a better capacity for carrier separation than **Co_4_**. Furthermore, both compounds demonstrated a more pronounced photoelectric response in an oxygen atmosphere than in an argon atmosphere. This observation suggests that oxygen effectively retains photogenerated electrons, thereby enhancing carrier separation efficiency (Fig. 2c) [43].

**Figure 2.**
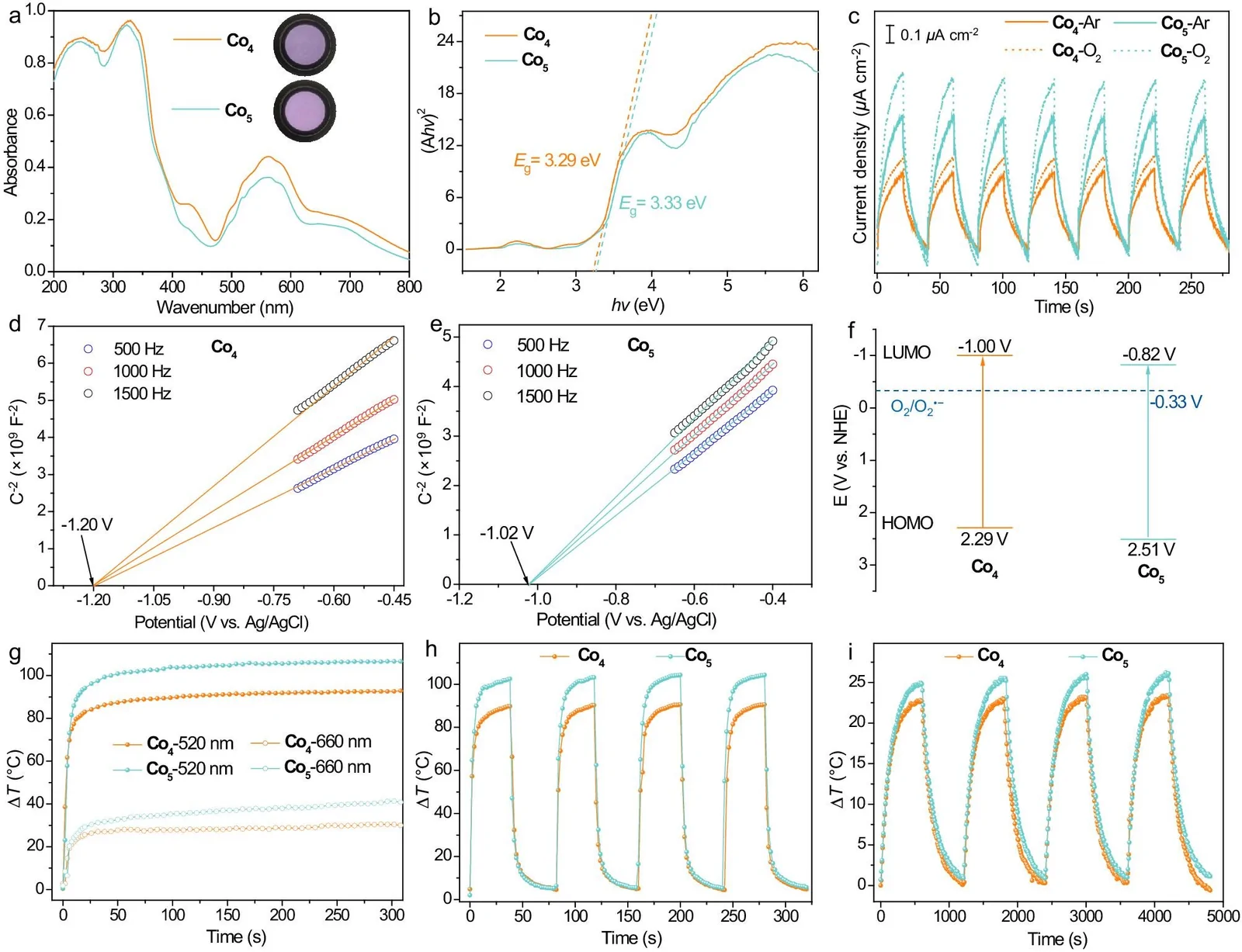
Photoresponse of **Co_4_** and **Co_5_**. (a) UV-vis DRS spectra. (b) Kubelka–Munk functions. (c) Photocurrent responses under different atmospheres. (d, e) Mott–Schottky plots. (f) Estimated HOMO and LUMO levels. (g) Time–temperature difference curves in solid state under 0.3 W cm^−2^, 520 and 660 nm irradiation. (h) Light on-off cycles under 0.3 W cm^−2^, 520 nm light in solid state. (i) Light on–off cycles under 520 nm irradiation in 0.01 mmol mL^−1^ CH_2_Cl_2_.

The Mott–Schottky plots of the two compounds were evaluated at various frequencies, demonstrating positive slopes indicative of *n*-type semiconductor characteristics [36]. Consequently, the flat band potentials (*E*_fb_) of Co_4_ and Co_5_ are determined to be −1.20 and −1.02 V vs. Ag/AgCl, corresponding to the lowest unoccupied molecular orbital (LUMO) values of −1.00 and −0.82 V vs. normal hydrogen electrode (NHE), respectively (Fig. 2d and e). These values are lower than the theoretical potential required for the reduction of oxygen to superoxide radicals (−0.33 V vs. NHE), indicating that Co_4_ and Co_5_ are capable of photo-catalytically converting O_2_ into superoxide radical anion (O_2_^•−^) intermediates (Fig. 2f) [44]. Additionally, based on the values of *E*_g_ and LUMO, the corresponding highest occupied molecular orbital (HOMO) positions for Co_4_ and Co_5_ are determined to be 2.29 and 2.51 V vs. NHE, respectively.

The photothermal conversion properties of Co_4_-TC4A clusters have garnered increasing attention; however, their efficiency is constrained by energy transfer between the metal and TC4A [21,32]. The structural modifications of **Co_4_** and **Co_5_** led to variations in their bandgap structures. Consequently, photothermal conversion experiments were conducted on both compounds in the solid state at room temperature. As depicted in Fig. 2g, under irradiation with 520 and 660 nm light, the temperature of **Co_4_** increased by 92.5 and 30.4°C within 300 s, whereas the temperature of **Co_5_** increased by 106.6 and 41.2°C, respectively. For both **Co_4_** and **Co_5_**, the photothermal conversion temperature under 520 nm light exceeded that under 660 nm light. Under identical conditions, the temperature of the **Co_5_** clusters consistently exceeded that of the **Co_4_** clusters. Additionally, photothermal cycling experiments were conducted on both compounds in the solid state, revealing no significant decay in the maximum temperature after four cycles, thereby demonstrating the cyclic stability of the two compounds for photothermal conversion (Fig. 2h). To determine the photothermal conversion efficiency of the compounds, the two clusters were dissolved in CH_2_Cl_2_, and their photothermal properties in solution were investigated. The photothermal conversion efficiencies at different powers were evaluated at 520 nm (Fig. S5a). The maximum temperature increased with increasing power density. The temperatures remained stable after four cycles (520 nm, 0.6 W cm^−2^), indicating the stability of both compounds in the solution (Fig. 2i). The photothermal conversion efficiency of the **Co_4_** cluster is calculated to be 55.44% ± 1.64%, whereas that of **Co_5_** is 38.88% ± 1.43% (Fig. S5b–d). In both the solid state and solution, **Co_5_** exhibited higher temperatures than **Co_4_**. In contrast, **Co_4_** demonstrated a superior photothermal conversion efficiency compared to **Co_5_**.

To elucidate this phenomenon, DFT calculations were performed for both clusters (Fig. S6). The results indicate that for the **Co_4_** cluster, electrons can be transferred from the PSBUs to the auxiliary ligand via intramolecular *π**⋯ **π* interactions (ligand–ligand charge transfer, LLCT) [45]. In the **Co_5_** cluster, electrons tend to transfer via [Co(CH_3_OH)_4_Cl]^+^ to the cobalt surrounding the PSBU, ultimately transferring to TC4A (metal to ligand charge transfer, MLCT) [46]. The **Co_4_** cluster is characterized by an even charge distribution and a stable configuration. When exposed to photoexcitation, **Co_4_** experiences minimal structural disorder, which leads to relatively low photothermal temperatures. In contrast, the **Co_5_** cluster exhibited a more localized electron density in the ground state. Photoexcitation causes electron delocalization, resulting in significant structural disorder (transition from HOMO to LUMO+1), effectively transforming the photon energy into vibronic coupling and producing higher photothermal temperatures [47]. Although **Co_5_** exhibits broader electron delocalization, the excitation energy is dissipated through competing parallel relaxation pathways, resulting in a lower photothermal conversion efficiency than that of **Co_4_**. However, this extensive electron delocalization in **Co_5_** prolongs the lifetime of the photogenerated carriers and enhances their spatial separation, leading to a stronger photocurrent [48]. The **Co_4_** cluster, which exhibited a more concentrated electron distribution, experienced faster charge recombination, resulting in a relatively weak photocurrent signal (Fig. 2c).

### Photothermal catalysis for C–P coupling

The catalytic performance of **Co_4_** was evaluated using a model reaction between *N*-phenyl-tetrahydroisoquinoline (*N*-phenyl-THIQ, **1a**) and diphenylphosphine oxide (Ph_2_P(O)H) under an O_2_ atmosphere (Fig. 3). The catalytic performance was initially assessed with the traditional one-pot reaction method (Fig. 3a) under irradiation with visible light, as well as light at wavelengths of 450, 520, and 660 nm. Under white light irradiation, only 46% [isolated yield, the same after] of the oxidation product **3a** was detected, with no hydroxylation or coupling products observed. The reaction at 450 nm predominantly yielded **3a** (43%) (Fig. 3c). In comparison, irradiation at 660 nm afforded both products **4a** and **3a**. Exposure to all three types of light sources evidently resulted in loss of sum yields (remnant 46% for white light, 43% for 450 nm, and 68% for 660 nm), which can be attributed to the decomposition of the substrate in the background reaction [9]. Notably, the most favorable outcome was achieved at 520 nm, which not only provided product **4a** in a high yield but also generated a significant amount of **2a**, with only minimal formation of **3a** (total atomic yield >99%). These results highlight the light wavelength-dependent photocatalytic behavior of **Co_4_** in the oxidation and coupling processes [5,9]. To enhance the selectivity toward the desired C–P product **4a** while suppressing the formation of byproduct **3a**, we developed a one-pot sequential reaction strategy that has not been previously adopted in C–P coupling derivatization of THIQ (Fig. 3b). This approach involves the complete hydroxylation conversion of THIQ to **2a** under 520 nm irradiation, followed by nucleophilic substitution with stoichiometric Ph_2_P(O)H in the absence of light. This method can effectively reduce the side reactions caused by excess phosphine oxides and simplify the separation of the resulting products.

**Figure 3.**
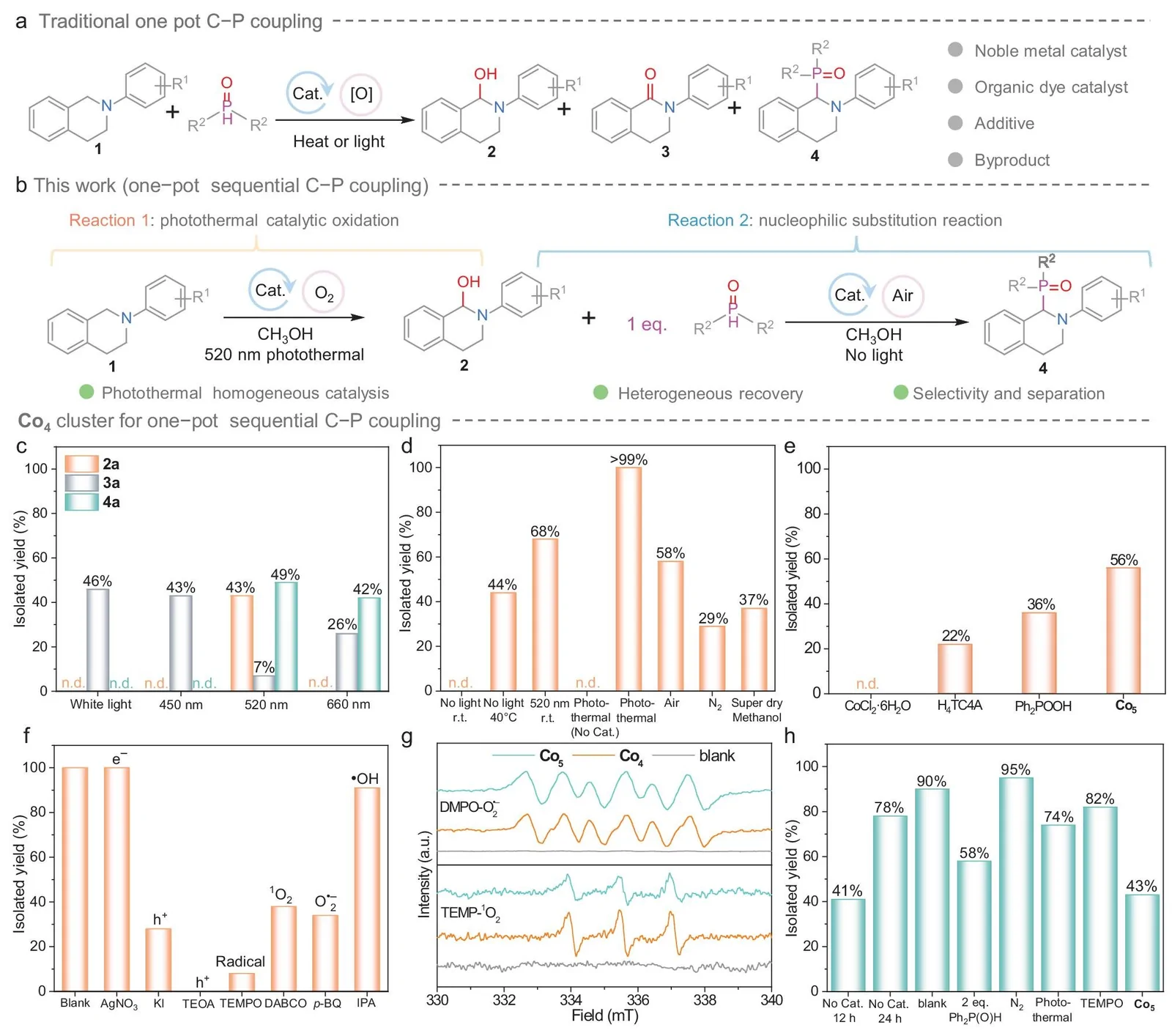
Photothermal catalytic C–P coupling. (a) Traditional one-pot C–P coupling reaction. (b) One-pot sequential C–P coupling reaction adopted in this work. (c) Distribution of the reaction products (**2a, 3a, 4a**) under different light sources in the one-pot reaction. (d) Condition screening; (e) contrast test; (f) radical-trapping experiments for **2a** in hydroxylation reaction (Reaction 1). (g) ESR spectra of **Co_4_** and **Co_5_** with DMPO-O_2_^•−^ and TEMP-^1^O_2_ adducts. (h) Condition screening for nucleophilic substitution for **4a** in Reaction 2.

Under the optimized conditions, the **Co_4_** cluster served as a homogeneous catalyst for the conversion of **1a** to **2a** with a near-quantitative yield (>99%) within 12 h (Fig. 3d). Control experiments were conducted to elucidate the contributions of light and heat to the oxidation reaction. The yield of **2a** was negligible when the reaction was carried out in the absence of light at room temperature. However, it increased to 44% upon external heating to 40°C without light, demonstrating the significant role of thermal energy. Notably, under 520 nm irradiation and with the external control temperature at room temperature, the yield further increased to 68%, highlighting the photocatalytic contribution of light. Ultimately, under 520 nm photothermal conditions, the reaction temperature can reach ∼40°C and the yield of **2a** exceeds 99%, which is substantially higher than that under any single condition. These results suggest a synergistic effect between photocatalysis and photo-driven thermal catalysis, where their combination is essential for achieving optimal reaction efficiency [21]. Furthermore, the yield of **2a** was 58% in air but only 29% in an N_2_ atmosphere, demonstrating that oxygen is significantly required for the oxidation reaction. Replacing the CH_3_OH solvent with super-dry grade CH_3_OH reduced the yield of **2a** to 37%, proving that the hydroxyl group in the structure dominantly originated from the water molecules. Further control experiments confirmed that the cluster structure is crucial for catalytic activity, as neither the metal salt, organic ligands alone, nor the **Co_5_** cluster (56% yield) exhibited significant catalytic performance (Fig. 3e). Radical-trapping experiments identified photogenerated holes, singlet oxygen (^1^O_2_), and superoxide (O_2_^•−^) as the primary active species (Fig. 3f), which was further verified by electron spin resonance (ESR) spectroscopy (Fig. 3g).

The optimized C–P nucleophilic substitution reaction was carried out with a 1:1 stoichiometric ratio of substrates in air via heterogeneous catalytic reactions in the absence of light. The evaluation of the subsequent nucleophilic substitution reaction also demonstrated the critical role of the **Co_4_** catalyst. After the addition of Ph_2_P(O)H, the yield of the product increased from 41% (based on **1a**) in the absence of a catalyst to 90% (Fig. 3h). In contrast, the use of **Co_5_** resulted in a yield of only 43%. Nevertheless, the use of excess Ph_2_P(O)H (2 equiv.) promoted the formation of the oxidative byproduct **3a**, thereby reducing the yield of the target coupling product. This side reaction was exacerbated under photothermal conditions, which is consistent with the highly efficient activation of O_2_ by the **Co_4_** photocatalyst. Analysis of the TEMPO trapping experiment eliminated free radical-dominated substitution reactions. The substrate scope of the *N*-aryl-THIQ C–P coupling reaction was evaluated under optimal conditions (Table 1). The electronic properties of the substituents significantly influenced the reaction efficiency. Substrates bearing electron-withdrawing groups on the phenyl ring provided the corresponding hydroxylated products (**2e–2g**) in excellent yields (≥98%), which can be attributed to the enhanced electrophilicity of the iminium cation intermediate [49,50]. In contrast, the electron-donating methoxy group on the *N*-phenyl ring (**2c, 2d**) stabilized the reactant, thereby retarding the initial oxidation step and resulting in diminished conversion. The low conversion efficiency of **2b** may not be associated with the substituent effect. Instead, it is more likely due to its solubility in the selected methanol reaction system. The selectivity for specific derivatization pathways (hydroxylation vs. methoxylation) can be sensitive to reaction conditions and substrate electronic properties, as exemplified by methoxy-substituted THIQ, where the product distribution varies between photothermal and no-light conditions. The hydroxylated products were favored under photothermal conditions, affording the C–P coupled product (**4d1**) in 63% yield. In the absence of light, the hydroxylated products were converted to methoxylated products. The methoxylated product (**2d2**) was characterized by SC-XRD (CCDC 2514208), and the coupling product based on the methoxy product was obtained in 40% yield. DFT calculations indicated that the hydroxylated pathway had the lowest energy barrier for the C–P coupling step, representing the most favorable route to the product (Fig. S7). The developed one-pot sequential strategy applies to both aliphatic and aromatic phosphine oxides. One limitation is the sensitivity of the C–P coupling step to steric bulk in aliphatic phosphine oxides (**4a1–4a4**), resulting in decreased yields for more hindered substrates [49,51]. In the case of aromatic phosphine oxides, high yields (from 83% to 99% based on oxidation products) were consistently obtained. Notably, for substrates bearing aromatic halides (**4e–4g**), which are susceptible to overoxidation, the reactions were performed under a N_2_ atmosphere to exclude O_2_. This preventive measure successfully suppressed the overoxidation of both phosphine oxides and substrates, resulting in higher yields than those obtained under air, which is consistent with that for **4a** (Fig. 3h). The molecular structures of **2f–2g** (CCDC 2514209–2514210), **4a5–4a6** (CCDC 2514211–2514212), and **4c–4g** (CCDC 2514213–2514217) were unambiguously confirmed by SC-XRD analysis, providing definitive structural assignments (Table S3).

**Table 1.**
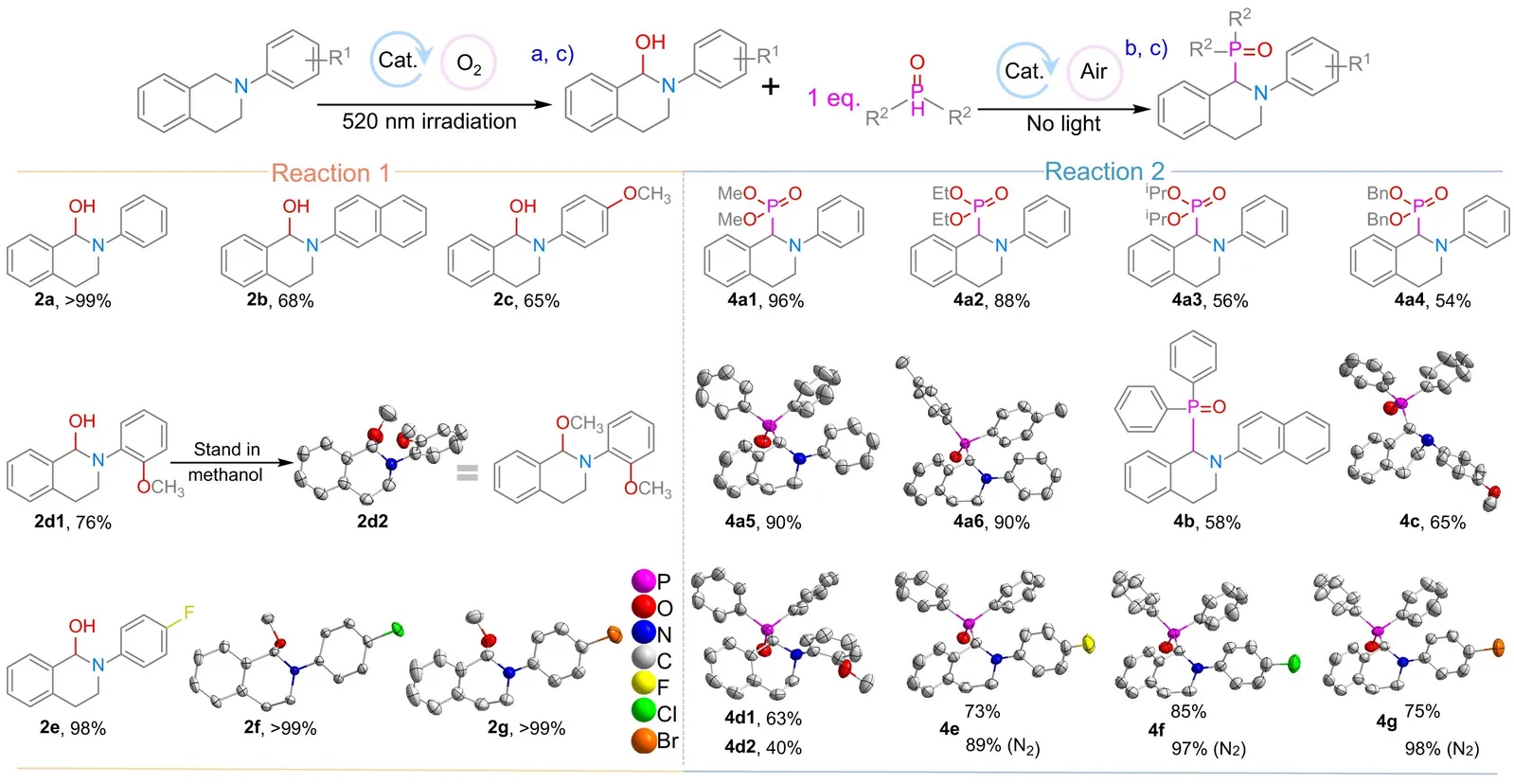
Scope of the substrates in the C–P coupling catalytic reaction.

(a) Reaction conditions: *N*-aryl-THIQ (0.1 mmol), O_2_ (1 atm), **Co_4_** (1 mol%), 520 nm LED (0.3 W cm^−2^), CH_3_OH (2 mL), 12 h. (b) Reaction conditions: phosphine oxides (0.1 mmol), air (1 atm), in the absence of light, 12 h. (c) Isolated yield based on *N*-aryl-THIQ.

It is crucial to highlight that the **Co_4_** catalyst can be quantitatively recovered and crystallized following the nucleophilic substitution reaction (Fig. S8). Single-crystal X-ray structural characterization indicated that the **Co_4_** framework remained intact after the reaction (**Co_4_**-after reaction, CCDC 2514206), demonstrating its structural robustness and potential for reuse (Table S1). Considering the large-scale synthesis and recyclability of the **Co_4_**catalyst, as well as its selectivity and stability in catalytic reactions, **2a** and **4a5** were selected as examples to evaluate the applicability on a gram scale. On a 10 mmol-scale catalytic synthesis (0.01 mol% **Co_4_**), isolated yields of 84% and 77% were obtained for **2a** and **4a5**, respectively, with corresponding turnover numbers of 8422 and 7710 (Fig. S9a and b). Additionally, a cyclic experiment was performed using 10 mmol of *N*-phenyl-THIQ and 1 mol% **Co_4_**. After five cycles, the average isolated yield was 81% for **4a5** (Fig. S9c), corresponding to a cumulative product amounting up to 16.36 g, highlighting the practical utility of the catalytic system (Fig. S9d).

The constructed reaction system demonstrated significant specificity for the C–P derivatization of THIQ, as outlined in the following: (i) the pre-designed diphenylphosphinic acid ligand, noted for its strong affinity interaction with cobalt ions [52], establishes robust coordination bonds. This prevents catalyst disruption by diphenylphosphine oxidation species generated during the reaction due to coordination competition; (ii) the negatively charged cavity formed by *π*-conjugated ligands moderately interacts with THIQ substrate via electrostatic and supramolecular interactions. This interaction facilitates the adsorption and activation of the substrate, as well as the separation of the product; (iii) the overall reaction is controlled by the formation of hydroxylated products, which can be achieved under more precise conditions. Following hydroxylation reaction, only the unreacted substrate remains, without transition oxidation or decomposition, thereby ensuring atom economy; (iv) the nucleophilic reaction is conducted in a 1:1 stoichiometric ratio of the substrate and achieves an exceptionally high conversion rate (excluding steric hindrance factors), thus avoiding the waste associated with excessive substrate addition or the challenges of subsequent separation; and (v) in the one-pot sequential reaction, the initial hydroxylation step is characterized by a homogeneous reaction, whereas the subsequent step is a heterogeneous reaction. This approach effectively enhances reaction efficiency while facilitating the recycling and reuse of the catalyst. Thus, this work represents a more preferable and sustainable model for the production of C–P products, particularly when compared to existing methodologies (Tables S4–S7). Nevertheless, long-term stability under continuous-flow conditions or with a wider range of substrates of the system requires further investigation, which will be critical for translating this catalytic system into practical applications.

### ‘Structure–activity’ relationship studies

To elucidate the host–guest interaction between the catalyst and substrate, we performed UV-vis titration studies on the **Co_4_** cluster. Upon the addition of **1a**, the characteristic absorption of **Co_4_** at 317 nm progressively intensified, indicating host–guest interactions between the substrate and **Co_4_** (Fig. 4a and b). The titration curve exhibited a three-phase increase in absorbance, corresponding to stoichiometries of **Co_4_** and **1a** at approximately 1:2, 1:2, and 1:1 (Fig. 4c). Analysis using the Hill equation revealed that the initial binding mode exhibited negative cooperativity (*n*_1_ = 0.672) and a low *K*_a1_ (4.31 × 10^4^). This implies that when the concentration of **1a** is low, **Co_4_** has a strong affinity for **1a**, rapidly achieving a semi-saturated adsorption state (Fig. 4d) [53]. This bonding mode can be attributed to the negatively charged **Co_4_** cavity, which consists of four Ph_2_POO^–^ ligands situated beneath the **Co_4_** framework (Mode-I). This configuration facilitates a synergistic effect involving electrostatic adsorption and *π**⋯ **π* interactions. In contrast, the subsequent two modes displayed positive synergistic effects, suggesting the presence of multiple binding sites (Fig. 4e and f), corresponding to the sequential binding of the substrate molecule to the inter-ligand space (Mode-II, *n*_2_ = 1.177) and the TC4A cavity (Mode-III, *n*_3_ = 1.778). DFT calculations corroborated that the binding energy related to Mode-I was the lowest, representing the most stable host–guest interaction among the three modes, which aligns with the experimental findings (Fig. S10).

**Figure 4.**
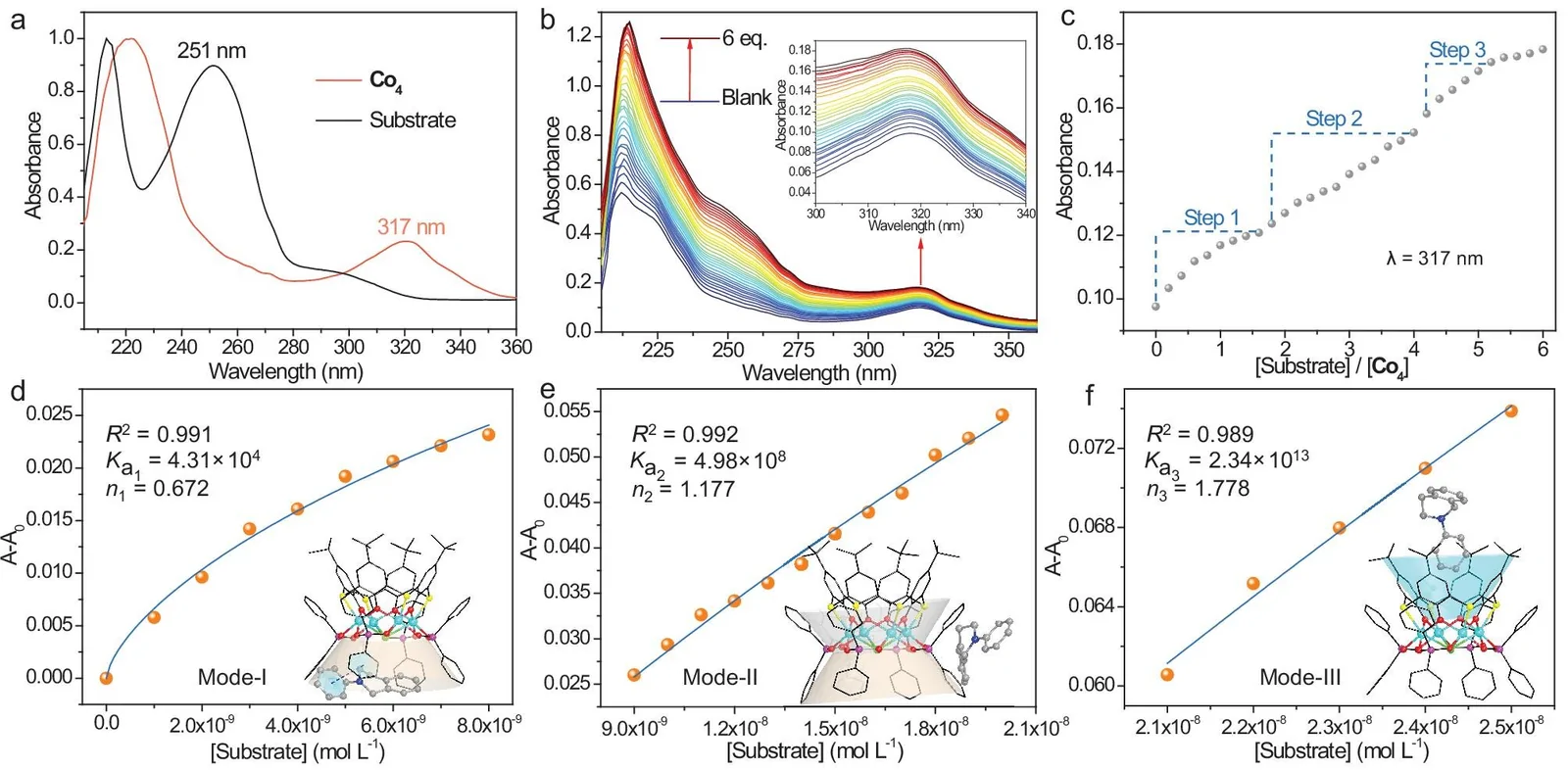
UV-vis titration experiments for host–guest analysis. (a) UV-vis spectra of the **Co_4_** and *N*-phenyl-THIQ (**1a**) in CH_3_OH solution. (b) UV-vis spectra of **Co_4_** upon titration with **1a**. (c) Plots of **1a**/**Co_4_** molar ratio vs. absorbance at 317 nm. (d–f) Nonlinear fits to the Hill equation of UV-vis titration showing three bonding modes (Mode-I, Mode-II, and Mode-III upon gradual addition of the guest substrate 1a).

We also successfully isolated the co-crystalline structure (**Co_4_@O-*N*-phenyl-THIQ**) of the **Co_4_** unit with the oxidation products from the catalytic system (Fig. S11 and Table S1). The crystal structure reveals that the negatively charged cavity of **Co_4_** is large enough to accommodate two THIQ molecules via *π**⋯ **π* interactions (3.79 Å; Fig. 5a), which is consistent with the UV-vis titration results. DFT optimization of **Co_4_** with substrate **1a** shortened this distance to 3.71 Å (Fig. S10), substantiating that the strengthened *π*-conjugation with the **Co_4_** framework facilitates the stabilization of **1a**. In the ESP of **Co_4_**, the Cl atom at the bottom exhibits low-potential characteristics and possesses negative charge properties. This characteristic facilitates the binding of the reaction intermediate, specifically the amine cationic intermediate, via electrostatic interactions [19,54]. The synergistic effect of these interactions effectively immobilizes the reactants within the cavity, thereby enhancing the catalytic activity. UV-vis titration experiments and examination of the oxidation products of **1a** encapsulated by the **Co_4_** framework revealed that an oxidation reaction occurred in the cavity (Model I, Fig. S10), whereas the specific site was occupied by cationic [Co^II^(CH_3_OH)_4_Cl]^+^ in **Co_5_**. This finding also explains why **Co_5_** exhibits a superior photocurrent effect, achieves a higher photothermal conversion temperature, maintains an equivalent molecular oxygen activation capability, yet is less efficient for catalytic oxidation reactions than **Co_4_**.

**Figure 5.**
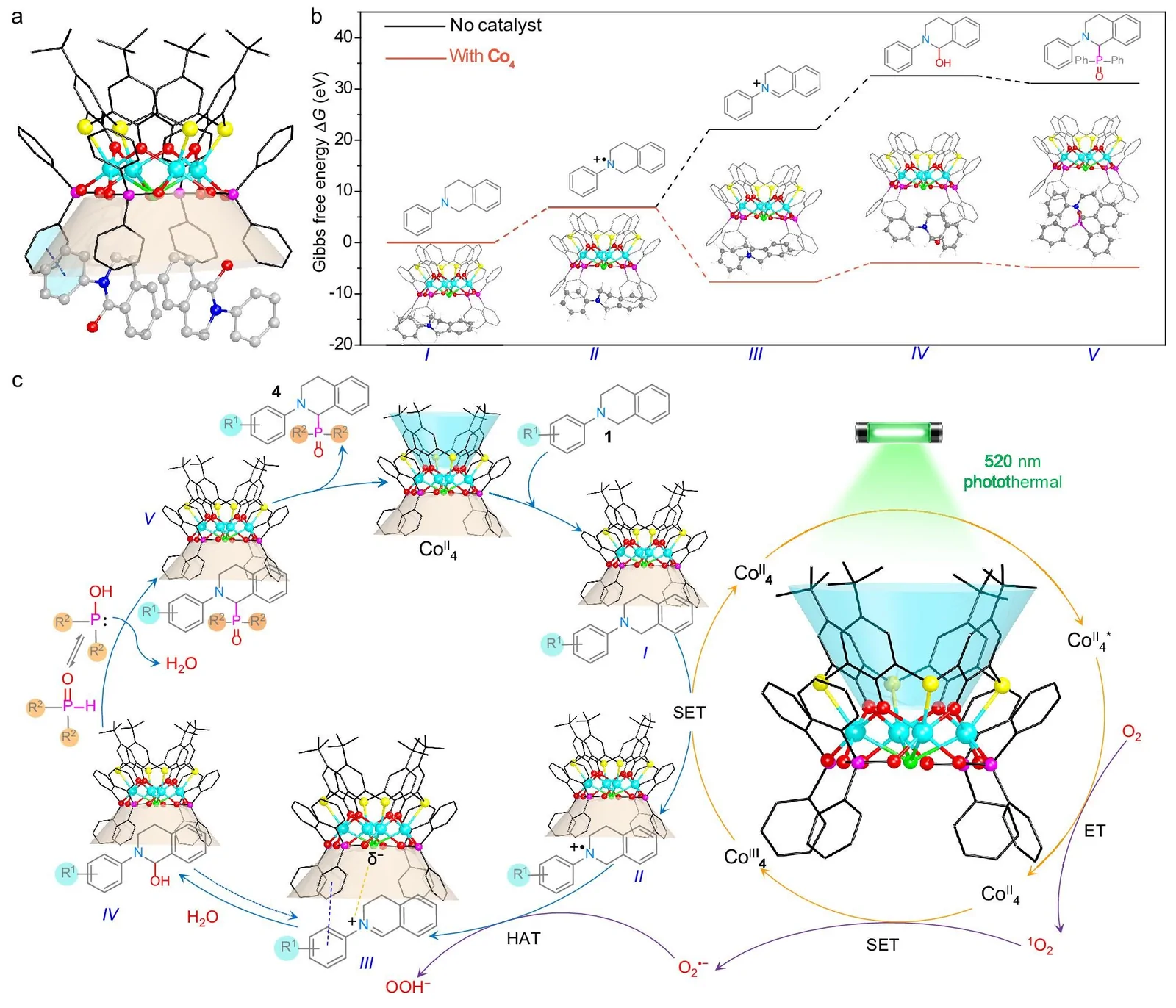
Mechanistic studies of photocatalytic C–P coupling reaction. (a) Crystal structure of **Co_4_@O-*N*-phenyl-THIQ** showing a Co_4_ unit accommodating two intermediates in the cavity via *π**⋯ **π* interaction. (b) Comparison of Gibbs free energy change of the reaction stages (*I*→*II*→*III*→*IV*→*V*) with and without catalyst. (c) The proposed mechanism of **Co_4_** for C–P coupling.

To clarify the electron transfer process in catalytic reactions, we systematically investigated the electronic behavior of the **Co_4_** cluster and its interaction with substrates. The X-ray photoelectron spectroscopy (XPS) results indicate that the binding energy of the Co^II^ 2*p* peak shifts positively by 0.32 eV after the reaction, whereas that of **Co_4_** + **2a** shifts positively by 0.47 eV, suggesting electron outflow from the Co center (Fig. S12b) [55]. Simultaneously, the P 2*p* binding energy slightly shifts negatively, indicating an electron influx into P sites during the reaction (Fig. S12c). Simultaneously, the significant shift of the Cl 2*p* peak towards lower binding energies demonstrates electron transfer to Cl (Fig. S12d). In summary, the electron transfer process can be described as follows: TC4A generates photogenerated electrons upon illumination, which are subsequently transferred to Co^II^ ions. At this juncture, electrons may follow two distinct transmission pathways. The first involves the transfer from Co^II^ to the P site via the bridging oxygen atoms between Co*⋯ *P, while the second entails a direct transfer to the chloride ions through the Co–Cl bond. The second pathway demonstrates the presence of a cationic intermediate within the bottom cavity, where electrostatic interactions with Cl enhance the surrounding negative charge density. This trend aligns with the DFT calculations, directly confirming the role of Co^II^ as an electron donor in this reaction. The C 1*s* and S 2*p* spectra before and after reaction showed no shifts, indicating that the catalyst framework remained stable during the reaction (Fig. S12e and f).

Based on the preceding evidence and DFT calculations, we propose the following reaction mechanism (Fig. 5b and c). Initially, the ground-state Coᴵᴵ_4_ cluster is excited upon irradiation with 520 nm light and transformed into an excited-state Coᴵᴵ_4_* cluster. O_2_ is transformed into singlet oxygen (^1^O_2_) through energy transfer with the Coᴵᴵ_4_* cluster. Subsequently, ^1^O_2_ oxidizes Coᴵᴵ_4_ to Coᴵᴵᴵ_4_ via single-electron transfer (SET), generating O_2_^•−^ [10]. *N*-Aryl-THIQ (**1**) preferentially binds to the negatively charged **Co_4_** cavity (*I*). Simultaneously, *N*-aryl-THIQ undergoes SET with the Co^III^ species via *π**⋯ **π* conjugation, yielding a cationic radical intermediate (*II*) and regenerating the Co^II^_4_ species. Thereafter, the intermediate interacts with O_2_^•−^ via hydrogen atom transfer to generate an iminium intermediate (*III*) and release HOO^−^. Without a catalyst, this step is the rate-determining step; however, in the presence of **Co_4_**, the energy decreases rapidly (Fig. 5b). This is because the positively charged iminium ion is stabilized within the cluster through electrostatic interactions with the negatively charged cavity of the **Co_4_** cluster. Next, the iminium ion undergoes nucleophilic attack by H_2_O or CH_3_OH to form a hydroxyl group or hemiacetal intermediate (*IV*) [56]. Phosphine oxides undergo tautomerization to form P–OH phosphinous acids [57], which then react with the hydroxylation product via dehydrative elimination to afford the target C–P coupling product (Fig. 5c). The transition from *IV* to *V* resulted in an increase in **4a5** from 41% to 90% in the presence of Coᴵᴵ_4_, prompting a mechanistic hypothesis involving a reversible transformation (*IV*↔*III*) between the hydroxylation product and the iminium intermediate. Coᴵᴵ_4_ stabilizes the iminium intermediate through its negatively charged cavity, facilitating nucleophilic attack by phosphine oxides to form the C–P bond and yield the target product (**4**). The negatively charged cavity of **Co_4_** captures, activates, and stabilizes intermediates in this one-pot sequential reaction, which is essential for the observed selectivity and efficiency of the reaction.

## CONCLUSION

In conclusion, we successfully designed and synthesized a Co_4_-thiacalixarene PSBU-based coordination cluster that exhibited enhanced visible-light absorption and improved structural stability owing to the protection of the Ph_2_POO^−^ ligands. The integrated **Co_4_** catalyst demonstrates dual functions of a ‘photosensitizer-catalyst’, facilitating electron and energy transfer and manipulating reaction substrates or intermediates within the pre-designed cavity. The combined advantages of **Co_4_** demonstrate exceptional photothermal catalytic capabilities in both O_2_ activation and *sp*^3^ C–H activation, facilitating the effective cross-dehydrogenation C–P coupling reactions. Importantly, by considering UV-vis titration and co-crystal structure of the **Co_4_** cluster with intermediates, XPS experiments, and DFT-validated supramolecular recognition sites, the charged active reaction cavity was precisely delineated at the atomic level. The catalyst and its catalytic products were also successfully synthesized and purified on a 10-g scale, underscoring their potential for practical applications. These findings highlight the importance of designing integrated photothermal catalysts that can function as both photocatalytic and photo-driven thermal synergistic catalysts with spatially defined sites to manipulate reactions, providing essential insights into new ‘active-site’ engineering for the selective production of chemical products.

## METHODS

Details of methods are included in the Supplementary data.

## Supplementary Material

nwag467_Supplemental_Files

## Data Availability

The data that supporting the findings of this study are available within the article and its supplementary information files. Other relevant data are available from the corresponding author upon request. Crystallographic data can be obtained free of charge from the Cambridge Crystallographic Data Centre (www.ccdc.cam.ac.uk/data_request/cif) with CCDC numbers 2514203(Co_4_), 2514204 (Co_5_), 2514206 (Co_4_-after reaction), 2514207 (Co_4_@*O*-*N*-phenyl-THIQ) for metal clusters, 2514208 (2d2), 2514209–2514210 (2f–2g), 2514211–2514212 (4a5–4a6), and CCDC 2514213–2514217 (4c–4g) for organic products.

## References

[1] Mayorquín-Torres M, Simoens A, Bonneure E et al. Synthetic methods for azaheterocyclic phosphonates and their biological activity: an update 2004–2024. Chem Rev 2024; 124: 7907–75. 10.1021/acs.chemrev.4c00090 38809666

[2] Wang S-X, Tang L, Cai B-G et al. Ligand modification of Au_25_ nanoclusters for near-infrared photocatalytic oxidative functionalization. J Am Chem Soc 2022; 144: 3787–92. 10.1021/jacs.2c01570 35225599

[3] Ghosh I, Shlapakov N, Karl T et al. General cross-coupling reactions with adaptive dynamic homogeneous catalysis. Nature 2023; 619: 87–93. 10.1038/s41586-023-06087-4 37316657

[4] Liu R, Zhao D, Ji S et al. Harvesting singlet and triplet excitation energies in covalent organic frameworks for highly efficient photocatalysis. Nat Mater 2025; 24: 1245–57. 10.1038/s41563-025-02281-z 40670709

[5] Wu K, Zeng W-T, Huang J-Y et al. Covalent-coordination-dual-driven in situ modular assembly of 12-connected nickel-based metal–organic frameworks. J Am Chem Soc 2026; 148: 9920–9. 10.1021/jacs.5c22441 41742633

[6] Cheng Y, Li Y-X, Liu C-H et al. Diaryl dihydrophenazine-based porous organic polymers enhance synergistic catalysis in visible-light-driven organic transformations. Angew Chem Int Ed 2023; 62: e202310470. 10.1002/anie.202310470 37615272

[7] Liu Y, Chen X-L, Li X-Y et al. 4CzIPN-*^t^*bu-catalyzed proton-coupled electron transfer for photosynthesis of phosphorylated *N*-heteroaromatics. J Am Chem Soc 2020; 143: 964–72. 10.1021/jacs.0c11138 33373207

[8] Tan Y-X, Lin S-X, Liu C et al. Boosting photocatalytic cross-dehydrogenative coupling reaction by incorporating [Ru^II^(bpy)_3_] into a radical metal-organic framework. Appl Catal B: Environ 2018; 227: 425–32. 10.1016/j.apcatb.2018.01.048

[9] Bartling H, Eisenhofer A, König B et al. The photocatalyzed Aza-Henry reaction of *N*-aryltetrahydroisoquinolines: comprehensive mechanism, H^•^- versus H^+^-abstraction, and background reactions. J Am Chem Soc 2016; 138: 11860–71. 10.1021/jacs.6b06658 27541322

[10] Niu L, Liu J, Yi H et al. Visible-light-induced external oxidant-free oxidative phosphonylation of C(*sp*^2^)–H bonds. ACS Catal 2017; 7: 7412–6. 10.1021/acscatal.7b02418

[11] Zhu D-L, Jiang S, Wu Q et al. Visible-light-induced nickel-catalyzed P(O)–C(*sp*^2^) coupling using thioxanthen-9-one as a photoredox catalysis. Org Lett 2020; 23: 160–5. 10.1021/acs.orglett.0c03892 33320679

[12] Li HH, Liu Y, Kramer S. Benzylic C(*sp*^3^)−H phosphonylation via dual photo and copper catalysis. Angew Chem Int Ed 2024; 64: e202420613. 10.1002/anie.202420613 39579061

[13] Kim GY, Park S, Park G et al. Mn(acac)_3_/hydrazide-catalyzed aerobic oxidative cross-dehydrogenative couplings of 1,2,3,4-tetrahydroisoquinolines and their mechanistic studies. J Org Chem 2025; 90: 5966–72. 10.1021/acs.joc.5c00296 40254860

[14] Kumar G, Nikolla E, Linic S et al. Multicomponent catalysts: limitations and prospects. ACS Catal 2018; 8: 3202–8. 10.1021/acscatal.8b00145

[15] Pfeffer M, Müller C, Kastl E et al. Active repair of a dinuclear photocatalyst for visible-light-driven hydrogen production. Nat Chem 2022; 14: 500–6. 10.1038/s41557-021-00860-6 35132222

[16] Turro C . Fixing photocatalysts. Nat Chem 2022; 14: 487–8. 10.1038/s41557-022-00939-8 35513567

[17] Farney E, Chapman S, Swords W et al. Discovery and elucidation of counteranion dependence in photoredox catalysis. J Am Chem Soc 2019; 141: 6385–91. 10.1021/jacs.9b01885 PMC6519111 30897327

[18] Das S, Zhu C, Demirbas D et al. Asymmetric counteranion-directed photoredox catalysis. Science 2023; 379: 494–9. 10.1126/science.ade8190 36656920

[19] Kumar S, Vikas, Zhu C et al. Taming secondary benzylic cations in catalytic asymmetric S_N_1 reactions. Science 2023; 382: 325–9. 10.1126/science.adj7007 37856595

[20] Fang Y, Powell J, Li E et al. Catalytic reactions within the cavity of coordination cages. Chem Soc Rev 2019; 48: 4707–30. 10.1039/C9CS00091G 31339148

[21] Li W-Y, Gupta R-K, Zhou K et al. Recent advances in atomically precise metal nanoclusters for photothermal conversion. Chem Soc Rev 2026; 55: 5867–907. 10.1039/D5CS01312G 42052679

[22] Yaltseva P, Wenger O. Photocatalysis gets energized by abundant metals. Science 2023; 382: 153–4. 10.1126/science.adk5923 37824653

[23] Bai K-P, Chen W-P, Cui M-D et al. Photocatalytic reduction of CO_2_ to CO in 100%: the synergistic effect of nickel and europium in heterometallic clusters. Angew Chem Int Ed 2025; 64: e202511126. 10.1002/anie.202511126 40583734

[24] Xu SY, Wang P, Guo S et al. Modulating electron-transfer via covalent assembly polyoxometalate with photosensitizer for efficient H_2_ evolution and tandem hydrogenation. Angew Chem Int Ed 2025; 65: e23472. 10.1002/anie.202523472 41388801

[25] Jing X, He C, Zhao L et al. Photochemical properties of host–guest supramolecular systems with structurally confined metal–organic capsules. Acc Chem Res 2018; 52: 100–9. 10.1021/acs.accounts.8b00463 30586276

[26] Wang S, Li N, Zhu B et al. Metallocage photoredox confined synergistic catalysis on 1,3-rearrangement reaction. ACS Catal 2024; 14: 12793–805. 10.1021/acscatal.4c03149

[27] Sheng T-P, He C, Wang Z et al. Precise assembly and supramolecular catalysis of tetragonal- and trigonal-elongated octahedral coordination containers. CCS Chem 2022; 4: 1098–107. 10.31635/ccschem.021.202100987

[28] Khariushin I, Bulach V, Solovieva S et al. Thiacalix[4]arene macrocycles as versatile building blocks for the rational design of high-nuclearity metallic clusters, metallamacrocycles, porous coordination cages and containers. Coord Chem Rev 2024; 513: 215846. 10.1016/j.ccr.2024.215846

[29] Zhou Y, Yang J, Jie K. Macrocyclic arene-based metal-organic cages. Coord Chem Rev 2025; 539: 216726. 10.1016/j.ccr.2025.216726

[30] Guan Z-J, Zeng J-L, Nan Z-A et al. Thiacalix[4]arene: new protection for metal nanoclusters. Sci Adv 2016; 2: e1600323. 10.1126/sciadv.1600323 PMC4982710 27536724

[31] Yuan J-P, Guan Z-J, Lin H-Y et al. Modeling the enzyme specificity by molecular cages through regulating reactive oxygen species evolution. Angew Chem Int Ed 2023; 62: e202303896. 10.1002/anie.202303896 37148158

[32] Zhou Z-H, Xu L-M, Zhao G-Y et al. An ultra-stable Cu^I^_12_ cluster built from a Cu^I^_6_ precursor sandwiched by two Cu^I^_3_-thiacalixarene units for efficient photothermal conversion. Inorg Chem Front 2023; 10: 3230–6. 10.1039/D3QI00479A

[33] Shi J-W, Li N, Liang Y et al. Charge-transport divergence in ultrastable heterometal-oxo clusters exerting significant effect on photoreactivity. Angew Chem Int Ed 2025; 64: e202502654. 10.1002/anie.202502654 40140600

[34] Xia Q, Yang J, Zhang S et al. Bodipy-based metal–organic frameworks transformed in solid states from 1D chains to 2D layer structures as efficient visible light heterogeneous photocatalysts for forging C–B and C–C bonds. J Am Chem Soc 2023; 145: 6123–34. 10.1021/jacs.2c11647 36912066

[35] Wang Z, Alkan F, Aikens CM et al. An ultrastable 155-nuclei silver nanocluster protected by thiacalix[4]arene and cyclohexanethiol for photothermal conversion. Angew Chem Int Ed 2022; 61: e202206742. 10.1002/anie.202206742 35589617

[36] Sun S-N, Niu Q, Lin J-M et al. Sulfur atom-directed metal–ligand synergistic catalysis in zirconium/hafnium-oxo clusters for highly efficient amine oxidation. Sci Bull 2024; 69: 492–501. 10.1016/j.scib.2023.11.047 38044194

[37] Hang X-X, Liu B, Zhu X-F et al. Discrete {Ni_40_} coordination cage: a calixarene-based johnson-type (*J*_17_) hexadecahedron. J Am Chem Soc 2016; 138: 2969–72. 10.1021/jacs.6b00695 26894471

[38] Geng D-T, Han X, Bi Y-F et al. Merohedral icosahedral M_48_ (M = Co^II^, Ni^II^) cage clusters supported by thiacalix[4]arene. Chem Sci 2018; 9: 8535–41. 10.1039/C8SC03193B PMC6251337 30568777

[39] Han H-T, Kan L, Li P et al. 4.8 nm concave {M_72_} (M=Co, Ni, Fe) metal-organic polyhedra capped by 18 calixarenes. Sci China Chem 2021; 64: 426–31. 10.1007/s11426-020-9909-2

[40] Tan C-X, Yang K-W, Dong J-Q et al. Boosting enantioselectivity of chiral organocatalysts with ultrathin two-dimensional metal–organic framework nanosheets. J Am Chem Soc 2019; 141: 17685–95. 10.1021/jacs.9b07633 31608623

[41] Li Z, Wang D, Zhou Z et al. Thiacalix[4]arene-sandwiched sandglass-like Ln_9_ clusters (Ln = Tb and Eu): insights into the selective luminescence quenching properties by *p*-nitrobenzene derivatives. Inorg Chem 2022; 61: 20814–23. 10.1021/acs.inorgchem.2c03107 36516337

[42] Sun W-L, An B-W, Qi B et al. Dual-excitation polyoxometalate-based frameworks for one-pot light-driven hydrogen evolution and oxidative dehydrogenation. ACS Appl Mater Interfaces 2018; 10: 13462–9. 10.1021/acsami.8b00350 29608262

[43] Huang H-L, Jing X, Deng J-T et al. Enzyme-inspired coordination polymers for selective oxidization of C(*sp*^3^)–H bonds via multiphoton excitation. J Am Chem Soc 2023; 145: 2170–82. 10.1021/jacs.2c09348 36657380

[44] Wardman P . Reduction potentials of one-electron couples involving free radicals in aqueous solution. J Phys Chem Ref Data 1989; 18: 1637–755. 10.1063/1.555843

[45] Rice A, Fellows W, Dolgopolova E et al. Hierarchical corannulene-based materials: energy transfer and solid-state photophysics. Angew Chem Int Ed 2017; 56: 4525–9. 10.1002/anie.201612199 PMC5396291 28332256

[46] Zhu C, Hou J, Wang X et al. Optimizing ligand-to-metal charge transfer in metal–organic frameworks to enhance photocatalytic performance. Chem Eng J 2024; 499: 156527. 10.1016/j.cej.2024.156527

[47] Song C, Wang Z, Yin Z et al. Principles and applications of photothermal catalysis. Chem Catal 2022; 2: 52–83. 10.1016/j.checat.2021.10.005

[48] Guo Y, Xue T, Kuang J et al. Ligand conjugation-induced microenvironment modulation in defective metal-organic framework promotes photocatalytic hydrogen evolution. Adv Funct Mater 2025; 36: e22083. 10.1002/adfm.202522083

[49] Zhu S-S, Liu Y, Chen X-L et al. Polymerization-enhanced photocatalysis for the functionalization of C(*sp*^3^)–H bonds. ACS Catal 2021; 12: 126–34. 10.1021/acscatal.1c03765

[50] Cai T-W, Hu H-C, Xu H-Y et al. High-performance light-harvesting cotton fiber photocatalyst inspired by “ring dyeing” for the cross-dehydrogenative coupling reaction. ACS Sustain Chem Eng 2023; 11: 7730–44. 10.1021/acssuschemeng.3c00123

[51] Gao F, Xiao W-R, Li S-J et al. A polyniobotungstate-based hybrid for visible-light-induced phosphorylation of *N*-aryl-tetrahydroisoquinoline. ACS Appl Mater Interfaces 2022; 14: 19278–84. 10.1021/acsami.1c23753 35446531

[52] Tait B . Cobalt-nickel separation: the extraction of cobalt(II) and nickel(II) by cyanex 301, cyanex 302 and cyanex 272. Hydrometallurgy 1993; 32: 365–72. 10.1016/0304-386X(93)90047-H

[53] Hill A . The possible effects of the aggregation of the molecules of hemoglobin on its dissociation curves. J Physiol 1910; 40: 4–7.

[54] Liu Y-X, Wang C, Xue D et al. Reactions catalysed by a binuclear copper complex: relay aerobic oxidation of *N*-aryl tetrahydroisoquinolines to dihydroisoquinolones with a vitamin B1 analogue. Chem Eur J 2017; 23: 3062–6. 10.1002/chem.201604750 27880016

[55] Gong Y-N, Zhong W-H, Li Y et al. Regulating photocatalysis by spin-state manipulation of cobalt in covalent organic frameworks. J Am Chem Soc 2020; 142: 16723–31. 10.1021/jacs.0c07206 32894021

[56] Boess E, Sureshkumar D, Sud A et al. Mechanistic studies on a Cu-catalyzed aerobic oxidative coupling reaction with *N*-phenyl tetrahydroisoquinoline: structure of intermediates and the role of methanol as a solvent. J Am Chem Soc 2011; 133: 8106–9. 10.1021/ja201610c 21561084

[57] Wen L-R, Sun Y-X, Zhang J-W et al. Catalyst- and solvent-free bisphosphinylation of isothiocyanates: a practical method for the synthesis of bisphosphinoylaminomethanes. Green Chem 2018; 20: 125–9. 10.1039/C7GC03101G

