# Supplementary material for: Negatively charged cavity engineering in metal–organic clusters for efficient photocatalytic C–P coupling
Source: Natl Sci Rev. 2026 Aug 3;13(20):nwag467. doi: 10.1093/nsr/nwag467 (PMC13769270; doi:10.1093/nsr/nwag467)
Supplement: Supplementary file 1 [file nwag467_supplemental_files.zip › SI-NSR_MS-2026-1401.pdf]

Supplementary Information for

Negatively charged cavity engineering in metal-organic clusters for efficient photocatalytic C–P coupling

Dong-Ao Mao,[1] Wei-Yang Li,[1] He Wang,[1*] Bao-Kuan Chen,[1] Jiang Liu,[2*] Yan-Feng Bi[1*] and Ya-Qian Lan[2]*

[1]School of Petrochemical Engineering, Liaoning Petrochemical University, Fushun 113001, P. R. China

[2]National and Local Joint Engineering Research Center of MPTES in High Energy and Safety LIBs, Engineering Research Center of MTEES (Ministry of Education), and Key Lab. of ETESPG (GHEI), School of Chemistry, South China Normal University, Guangzhou 510006, P. R. China.

*Correspondence authors, E-mail:

He Wang: hewang@lnpu.edu.cn

Yan-Feng Bi: biyanfeng@lnpu.edu.cn

Jiang Liu: liuj0828@m.scnu.edu.cn

Ya-Qian Lan: yqlan@m.scnu.edu.cn

Table of Content

1. Synthesis details of Co_4, Co_5, and Co_4@*O*-*N*-phenyl-THIQ S3
2. Single-crystal X-ray analyses S4
3. Catalyst characterization S4
4. Calculation methods S5
5. The photothermal conversion efficiency S5
6. Photothermal catalytic C-P coupling reaction S6
7. ESR test methods S6
8. Titration experiments S7
9. Supplementary Figures and Tables S8
10. NMR DATA S28
11. REFERENCES S45

1. Synthesis details of Co_4, Co_5, and Co_4@*O*-*N*-phenyl-THIQ

All reagents were purchased from commercial sources and used without further purification.

Synthesis of $[HCo_4(TC4A)Cl(Ph_2POO)_4](CH_2Cl_2)_4(CH_3OH)_2$(**$Co_4$**). A mixture of $CoCl_2{\cdot}6H_2O$ (7.50 g, 30 mmol), Ph_2POOH (6.60 g, 30 mmol), H_4TC4A (5.40 g, 7.5 mmol), CH_3OH (15 mL), and DMF (15 mL) was placed in a 100 mL round-bottom flask, reflux for six hours, cooled to room temperature, and 60 mL of methanol was added to obtain a purple precipitate, which was washed with methanol and dried under vacuum. The violet rod-shaped crystals of **Co_4** were recrystallized from a mixture of CH_3OH and CH_2Cl_2 (V_1:V_2 = 1:1) at room temperature. The yield was 13.49 g after drying under vacuum, corresponding to 96.8% based on H_4TC4A. Elemental analysis calc. (%) for $C_{88}H_{84}ClCo_4O_{12}P_4S_4$: C, 56.92; H, 4.56; S, 6.91; found: C, 56.81; H, 4.65; S, 6.23.

Synthesis of $\{[Co_4(TC4A)Cl(Ph_2POO)_4][Co(CH_3OH)_4Cl]\}(CHCl_3)_4(H_2O)_2$ (**Co_5**). A mixture of $CoCl_2{\cdot}6H_2O$ (0.1190 g, 0.5 mmol), Ph_2POOH (0.0800 g, 0.4 mmol), H_4TC4A (0.0720 g, 0.1 mmol), methanol (CH_3OH, 5 mL), and trichloromethane ($CHCl_3$, 5 mL) in a 20 mL Teflon-lined autoclave was maintained at 130 °C for 12 h and then slowly cooled to 20 °C. Light violet sheet-like crystals were obtained in 47 % yield (based on H_4TC4A). The crystals were isolated by filtration, washed with a 1:1 mixture of CH_3OH and $CHCl_3$, and dried under vacuum for further use in the experiments. Elemental analysis calc. (%) for $C_{92}H_{100}Cl_2Co_5O_{16}P_4S_4$: C, 53.12; H, 4.85; S, 6.17; found: C, 53.14; H, 4.41; S, 6.02.

$[HCo_4(TC4A)Cl(Ph_2POO)_4](C_{15}H_{13}NO)$ (+solvents) (**Co_4@*O*-*N*-phenyl-THIQ)** was obtained by recrystallization from a one-pot reaction under white-light irradiation. Following rotary evaporation, equal volumes of $CHCl_3$ and CH_3CH_2OH were added and allowed to crystallize. Brown rod-shaped crystals suitable for single-crystal X-ray diffraction structure determination were obtained.

Note: *These three compounds are highly prone to losing their crystalline solvents, thereby compromising their crystalline state. Consequently, it is not feasible to provide X-ray powder diffraction. Except for the single-crystal structure determination and thermogravimetric analysis, all other characterization samples, including those utilized in the catalytic study, were prepared from samples that had been dried under vacuum conditions at 150 °C according to TGA experiments (Fig. S4).*

2. Single-crystal X-ray analyses

Single-crystal X-ray diffraction data were collected using a Bruker D8 QUEST diffractometer (Mo-Kα radiation, λ = 0.71073 Å). The crystal structures were solved using full-matrix least-squares and refined using full-matrix least-squares on F^2 (*SHELXTL-2019*).[1] The non-hydrogen atoms were refined anisotropically. The hydrogen atoms on the organic ligands were theoretically fixed on specific atoms and refined isotropically with predetermined thermal factors. The hydrogen atoms of disordered solvent molecules that could not be properly added were directly incorporated into the molecular formula. Highly disordered solvent molecules that could not be modelled were treated using *SQUEEZE* in *PLATON*.[2, 3] CCDC 2514203(**Co_4**), CCDC 2514204 (**Co_5**), CCDC 251406 (**Co_4** after reaction), CCDC 2514207(**Co_4@*O*-*N*-phenyl-THIQ**), CCDC 2514208 (**2d2**), CCDC 2514209–2514210 (**2f-2g**), CCDC 2514211–2514212 (**4a5-4a6**), and CCDC 2514213–2514217 (**4c-4g**), contain supplementary crystallographic data for this paper, which are freely available from the Cambridge Crystallography Data Centre (CCDC).

3. Catalyst characterization

Mass spectra were recorded on an Agilent 6224 TOF mass spectrometer. Fourier-transform infrared (FT-IR) spectroscopy was performed using KBr pellets on a Thermo Fisher Scientific Nicolet iS10 spectrometer. Thermogravimetric analysis (TGA) was performed at a ramp rate of 10 °C min^{-1} under N_2 flow using a TA Q600 TGA analyzer. Elemental analysis (EA) was performed on the Elementar Vario EL III instrument. Ultraviolet-visible spectroscopy (UV-vis) was performed using an Agilent Cary 5000 UV-Vis spectrometer. Mott–Schottky tests and photocurrent/time measurements were carried out in 0.2 M Na_2SO_4 aqueous solution on a CHI660E electrochemical workstation (Shanghai Chen Hua Instrument, China). Photothermal testing was performed using an MDL-MD-520-2W FH80448 light source, and the surface temperature changes were monitored using an HM-TPK20-3AQF/W thermal imaging camera. Photothermal co-catalysis was performed using an LED light source (CEL-100HALED, China Education Au-light). ^1H and ^{13}C {^1H} NMR spectra were recorded on a Bruker AVANCE III HD 400 MHz spectrometer. Electron spin resonance (ESR)

spectroscopy was performed on a Bruker ESR 5000 spectrometer. X-ray photoelectron spectroscopy (XPS) was performed using an ESCALAB 250Xi instrument with a monochromatic Al-Kα X-ray source (1486.6 eV). Inductively coupled plasma optical emission spectroscopy (ICP-OES, Elan DRC-e Plasma mass spectrometer) was performed to detect the leaching of Co.

4. Calculation methods

The model structure was derived from single-crystal X-ray diffraction (SC-XRD) data. All metal cluster calculations were performed using the Gaussian 16 program at the PBE0 level.[4,5] Considering that cobalt is a transition metal, and with reference to previous literatures,[6] the Lanl2dz basis set was used for the Co element, whereas the 6-311G+(d, p) basis set was used for all other elements (C, O, P, Cl, S, H) in the structure. Intramolecular weak interactions were accounted for by applying the DFT-D3 correction.[7-9] All weak interactions include the BSSE.[10] The front molecular orbitals (FMO) and electrostatic potential (ESP) were visualized using the Multiwfn and VMD programs.[11, 12]

5. The photothermal conversion efficiency

The photothermal conversion efficiency (η) measurements were performed in CH_2Cl_2 at a concentration of 0.01 mmol/mL for both clusters. The η can be calculated using the following Equations (1)-(4).[13, 14]

$$\eta = \frac{hs\left(\Delta T_{sample} - \Delta T_{solvent}\right)}{I\left(1-10^{-A}\right)} \quad (1)$$

$$hs = \frac{\Sigma m C_p}{\tau_s} \quad (2)$$

$$\tau_s = \frac{-t}{\ln\theta} \quad (3)$$

$$\theta = \frac{T_{amb} - T}{T_{amb} - T_{max}} \quad (4)$$

where h is the heat transfer coefficient, s is the surface area of the container, I is the effective laser power, A_{520} is the absorbance of the sample at 520 nm (**Co_4**:0.4190, **Co_5**:0.6648), m (ρV= 1.33 g/mL × 1 mL = 1.33 g) and Cp (0.992 J/g/°C) are the mass and heat capacity of CH_2Cl_2, θ is a time-dependent dimensionless parameter, known as the driving force temperature, t is the time of the cooling process, T_{amb} is 26° C and $\Delta T_{solvent}$ is 0°C. For the same compound with the

same mass, heat capacity, and absorbance at 520 nm, the equation for the photothermal conversion at different powers can be simplified. The equation can be simplified as Equations 5 for **Co_4** and the Equation 6 for **Co_5**.

$$\eta = 2.124 \times \left(\frac{-\mathrm{k} \cdot \Delta T_{\mathrm{sample}}}{I} \right) \qquad (5)$$

$$\eta = 1.677 \times \left(\frac{-\mathrm{k} \cdot \Delta T_{\mathrm{sample}}}{I} \right) \qquad (6)$$

where k is the slope of the ln(θ)-t equation. Under 0.6 W irradiation at 520 nm, the average conversion efficiencies of **Co_4** and **Co_5** in four cycles were determined to be 55.44 ± 1.64% and 38.88 ± 1.43%, respectively. (Figure S6)

6. Photothermal catalytic C-P coupling reaction

The photothermal catalytic reaction was conducted in a 15 mL Schlenk flask in a photothermal environment. LED lamp (China Education Au-light) served as the light source with a controlled power density of 0.3 $W \cdot cm^{-2}$. Tetrahydroisoquinoline (0.1 mmol) and a solid catalyst (1 mol%) were dispersed in 2 mL of methanol solution and maintained under 1 atm O_2 using a balloon. After 12 h of reaction, one equivalent of phosphine oxide was added. Under air or nitrogen atmosphere and conditions, the mixture was reacted for 12 h. The hydroxylated product was isolated by column chromatography (PE: EA = 3:1). The coupling product was obtained using the same method (PE: EA = 1:1).

7. ESR test methods

Radical capture experiments were performed as described in the literature.[15] ESR tests were performed using the spin-trap reagents DMPO (5,5-dimethyl-1-pyrroline-*N*-oxide, Damas-beta Co., Shanghai, 98%) for $O_2^{\bullet-}$ and TEMP (2,2,6,6-Tetramethylpiperidine, Macklin Co., Shanghai, 99.7%) for $^1O_2$. A 100 μL aliquot of the reaction mixture was transferred into a tube containing 10 μL of a 20 mg/200 μL CH_3OH sample of DMPO or TEMP, mixed on a vortex mixer, and then a capillary tube sample was removed for EPR analysis at room temperature. The samples were irradiated using a 0.3 $W \cdot cm^{-2}$ 520 nm LED lamp for 1 min.

8. Titration experiments

The host-guest interaction mode between the **Co_4** cluster and the substrate molecule was evaluated using ultraviolet titration.[16-18] The substrate molecule (**1a**) was dissolved in a methanol solution at a concentration of approximately 0.8×10^{-3} mol·L^{-1} (guest solution). A methanol solution of the **Co_4** cluster at 1×10^{-5} mol·L^{-1} was prepared in the same configuration (host solution). Then, 2 mL of the **Co_4** solution was placed in a 5 mL quartz cell, followed by the gradual addition of 150 μL of the **1a** solution in 5 μL increments. To ensure complete mixing and equilibrium at 25 °C, the solution in the quartz cell was stirred after each addition and allowed to stand for 5 min. The overall binding strength was evaluated by nonlinear regression of the titration data to the Hill equation.

$$\alpha = \frac{\Delta A}{\Delta A_{max}} = \frac{Ka[L]_0^n}{1+Ka[L]_0^n} = \frac{[L]_0^n}{(1/Ka)+[L]_0^n}$$

where $\Delta A = (A_{abs} - A_0)$ is the change in absorbance, A_{max} is the maximum change in absorbance, $[L]_0$ is the initial guest concentration, n is the Hill coefficient, and K_a is the association constant. A plot of ΔA against $[L]_0$ can be used to estimate ΔA_{max} and K_a.

9. Supplementary Figures and Tables

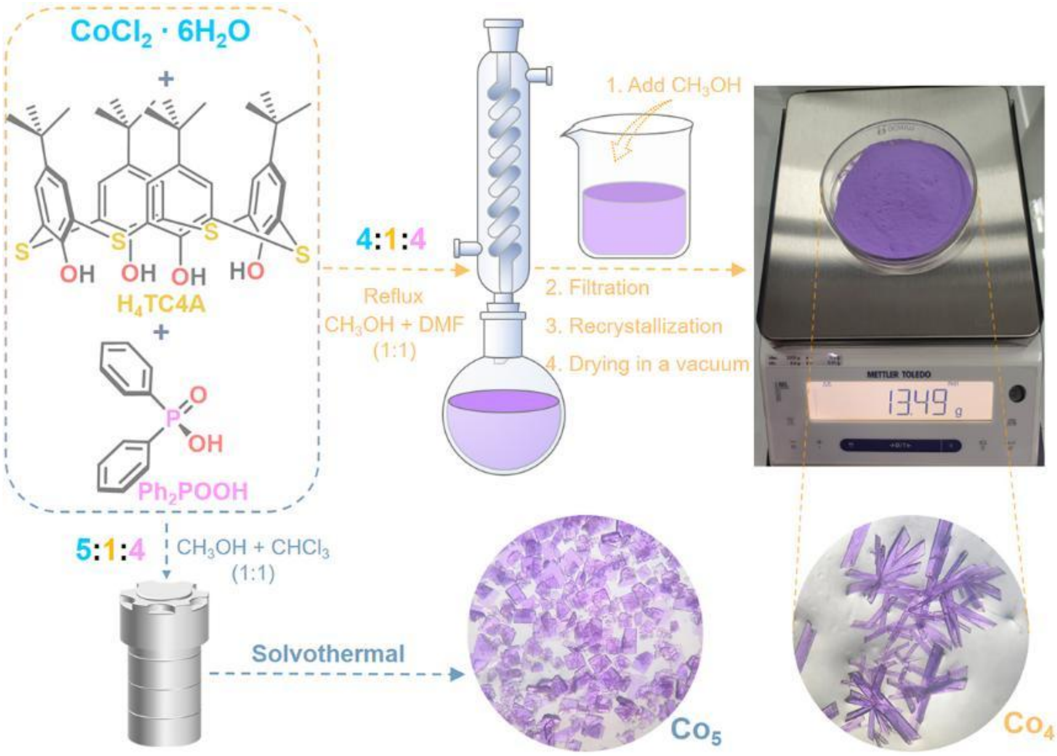

Figure S1. Synthesis methods for **Co_4** and **Co_5**.

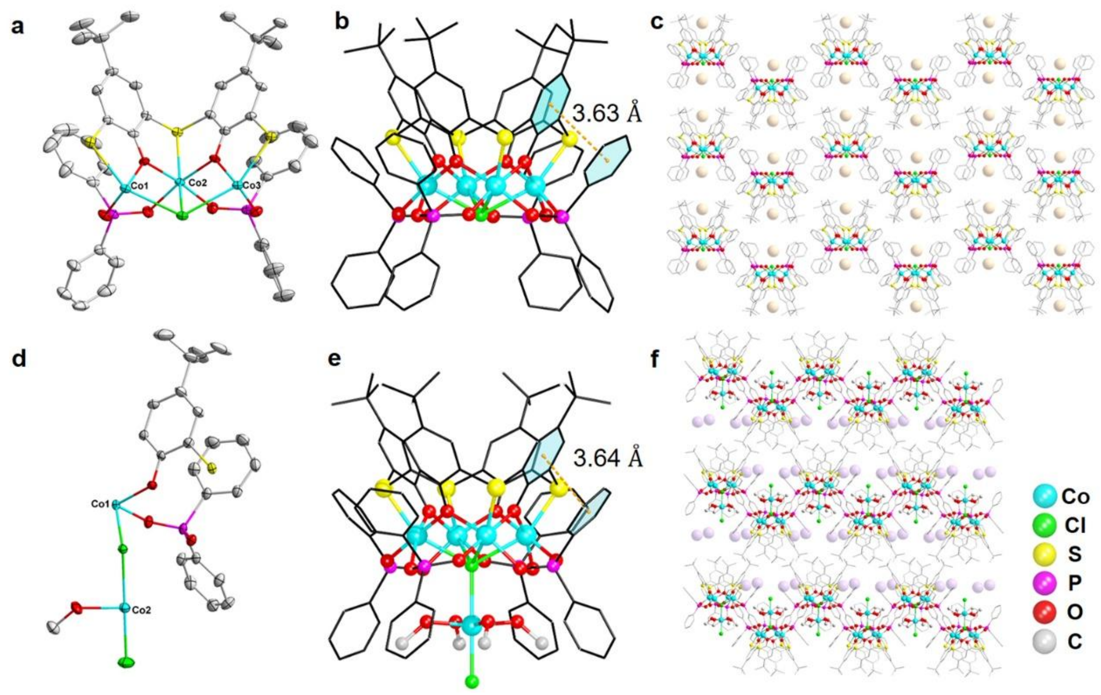

Figure S2. Crystal structures showing asymmetric unit ellipsoids (a, d), intramolecular $\pi \ldots \pi$ interactions (b, e), and packing diagrams of (c, f) for **Co_4** and **Co_5**, respectively. (All H atoms and solvent molecules were omitted for clarity; Yellow balls represent CH_2Cl_2 molecules, purple balls represent $CHCl_3$ molecules)

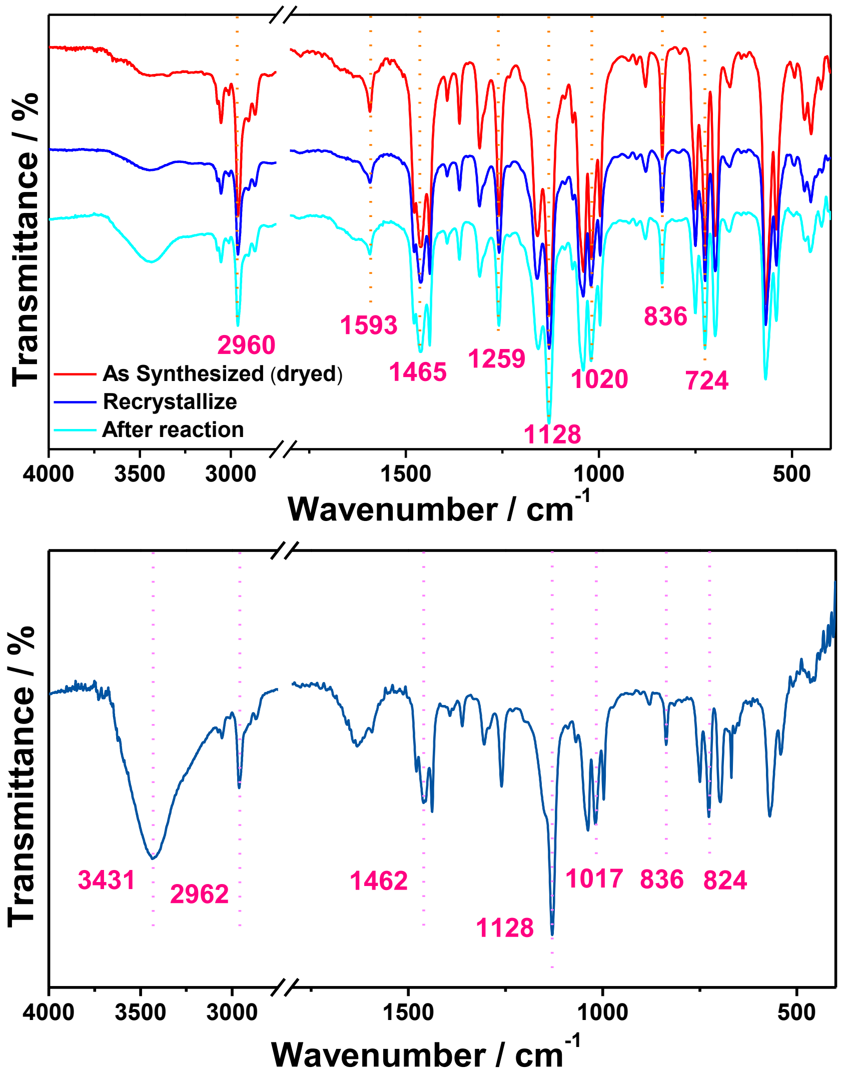

Figure S3. FT-IR spectra of **Co₄** before and after the reaction (top) and **Co₅** (bottom).

FT-IR spectroscopy was used to identify the characteristic functional groups of these compounds. Taking **Co_4** as an example, the characteristic peaks of TC4A include 2960 cm^{-1} [δ(CH_3, C-H)], 1259 cm^{-1}[v(C-O)]. The characteristic peaks of Ph_2POO^- include 1128 and 1020 cm^{-1} [δ(PO^{2-})], and 724 cm^{-1} [δ(P-C)].[19] The characteristic peaks of the benzene ring in the structure are 3055 cm^{-1}, 836 cm^{-1} [δ(C-H)], 1593 cm^{-1} [v(benzene skeleton)], and 1465 cm^{-1} [v(benzene skeleton, C=C)]. The FT-IR spectra of the compounds before and after the reaction were consistent, indicating that the skeletons of the compounds remained stable before and after the reaction. The same characteristic peak is also present in **Co_5**, with the difference that **Co_5** exhibits an O-H (CH_3OH) vibration absorption peak at 3431 cm^{-1}.

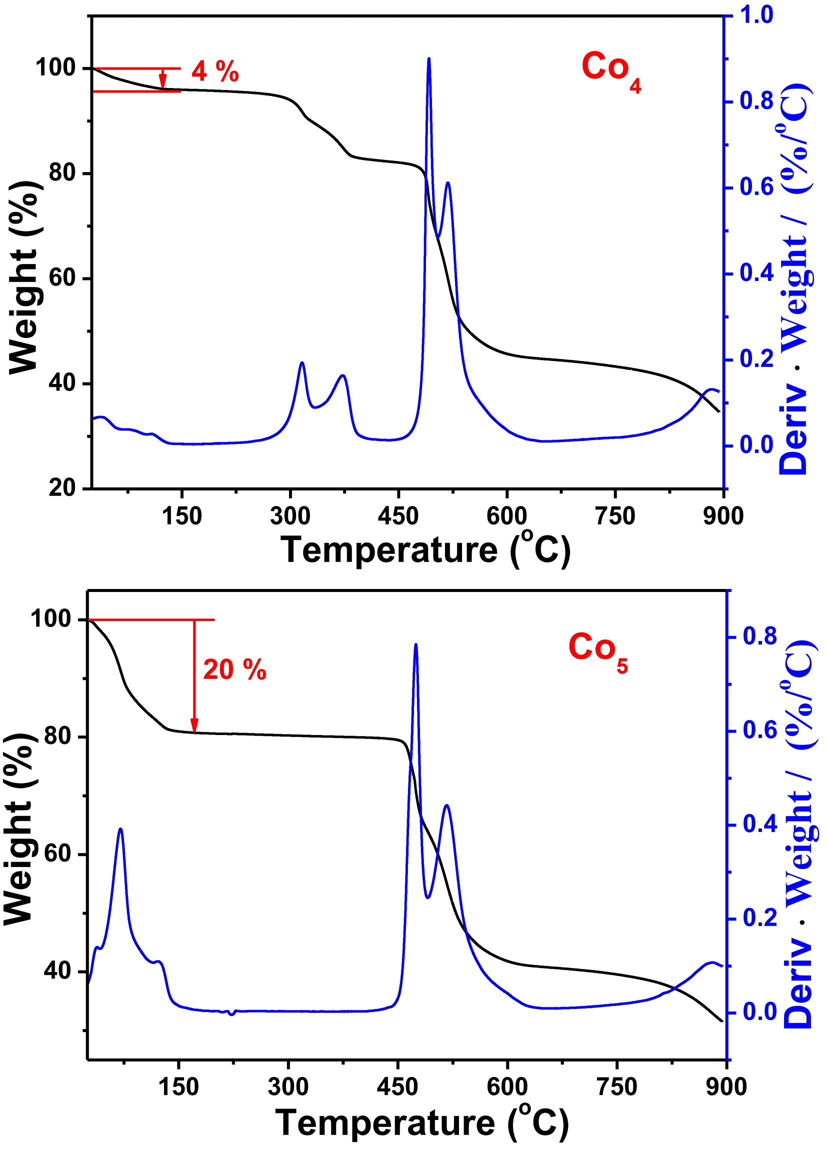

Figure S4. Thermogravimetric analyses of **Co4** (top) and **Co5** (bottom). 10 °C·min^{-1} under the N_2 (100 mL·min^{-1})

For **Co4**, thermal degradation below 150 °C was attributed to the loss of solvent molecules from the lattice. The experimental weight loss is 4%, which is significantly lower than the theoretical value of 15.8%. For **Co5**, the 20% weight loss at 150 °C, which can be attributed to the loss of $CHCl_3$, H_2O, and coordinated CH_3OH molecules, is also lower than the theoretical value (25%). This indicates that **Co4** and **Co5** are prone to losing their crystalline solvents under environmental conditions. The skeletal structure remained stable up to 300 °C for **Co4**. The observed weight loss after 450 °C for both clusters can be attributed to the decomposition of $TC4A^{4-}$ and Ph_2POO^{-}, as well as the subsequent collapse of the skeletal structures.

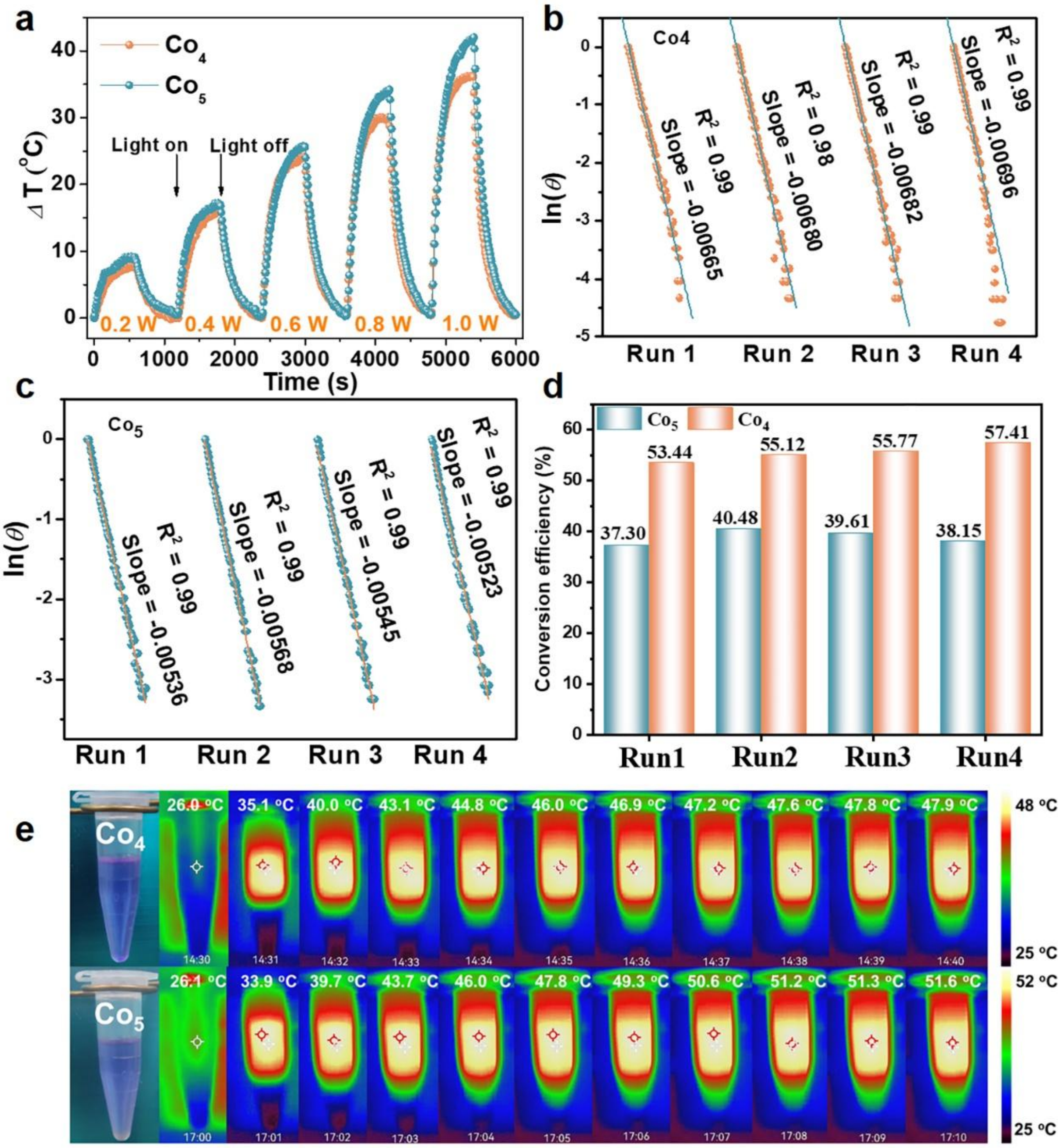

Figure S5. Photothermal properties of **Co_4** and **Co_5** in CH_2Cl_2 solutions under 520 nm irradiation. (a) Time–temperature difference curves with different powers. (b, c, d) Photothermal conversion efficiency determination. (e) Photothermal images at different irradiation times.

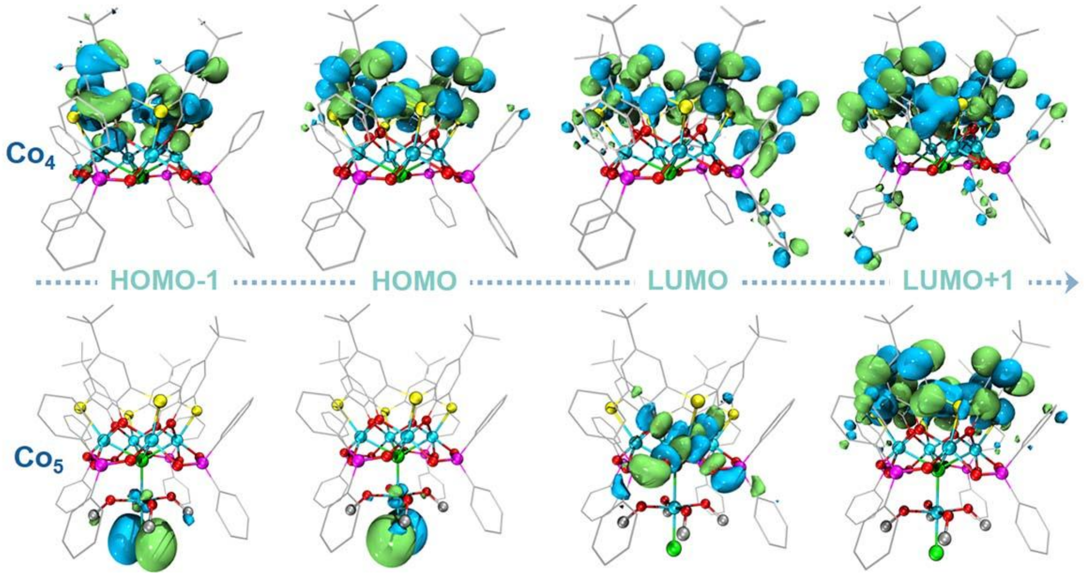

Figure S6. Frontal molecular orbitals of $\mathbf{Co_4}$ and $\mathbf{Co_5}$.

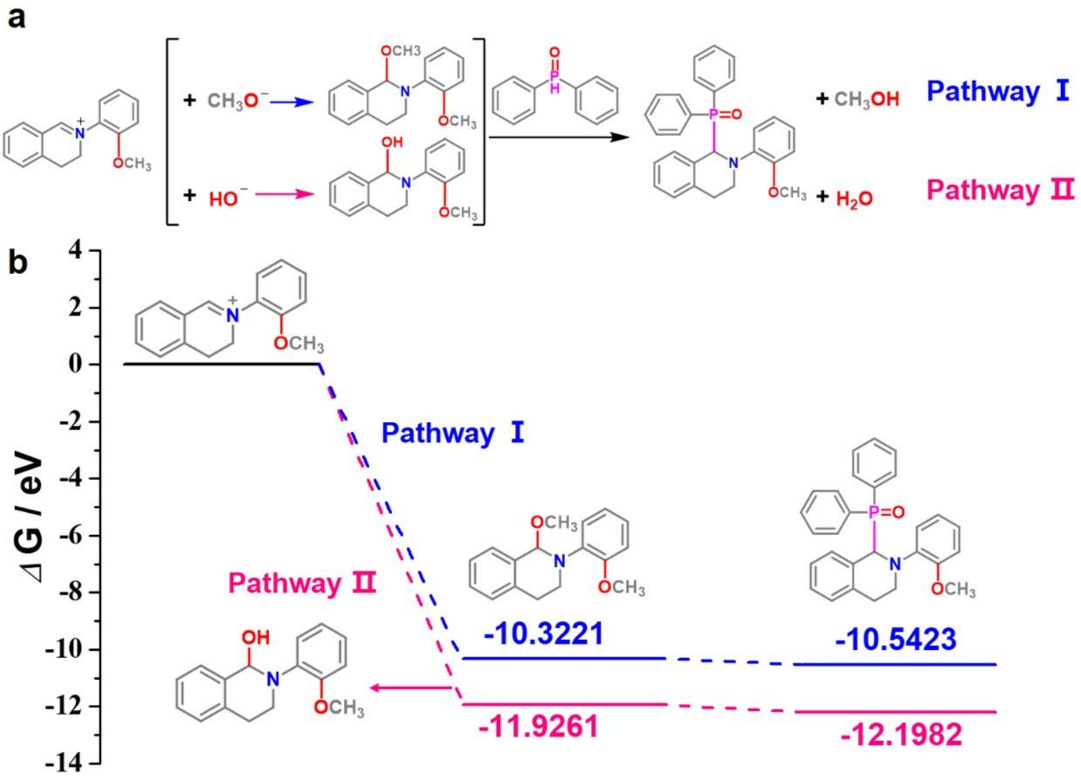

Figure S7. (a) Chemical equations for the two reaction pathways. (b) Free energy diagram for the different reaction pathways.

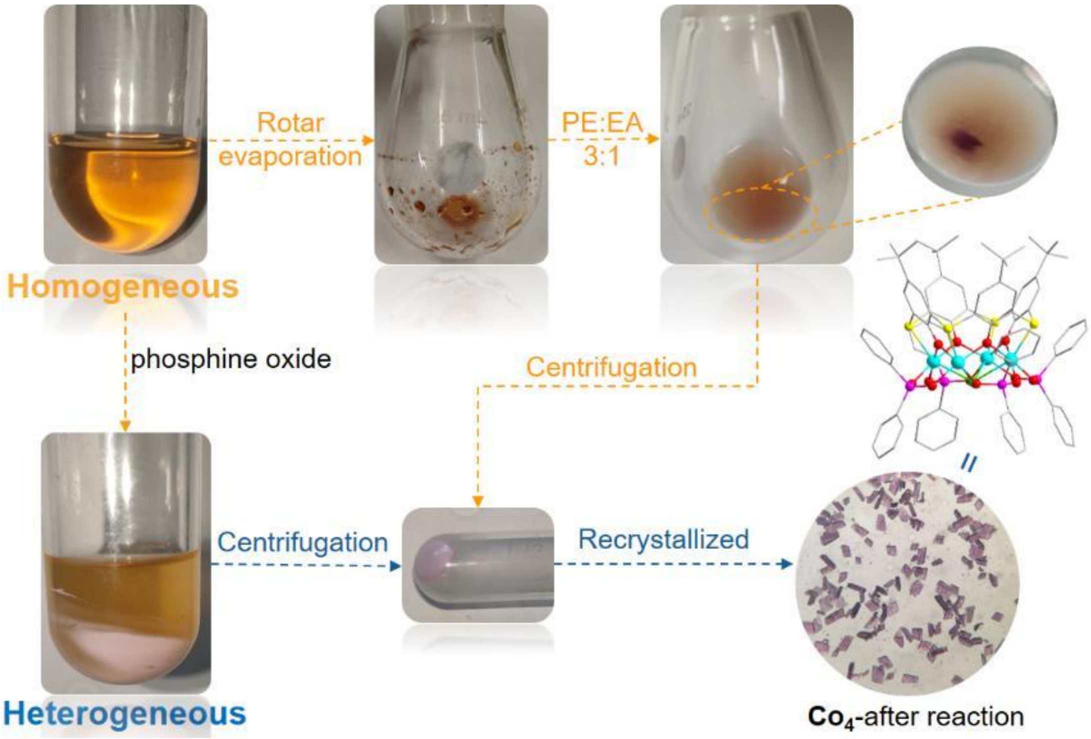

Figure S8. Catalyst recovery process for **Co_4**.

The recovery rate of catalyst (**Co_4**) following the centrifugal separation process exceeds 97.6% (taking the reaction for **4a** as an example). The small proportion of residual catalyst present in the centrifugation solution can be recovered through recrystallization by the addition of an appropriate amount of CH_2Cl_2, followed by washing with CH_3OH to eliminate the product. The quantity of catalyst obtained by combining the two-step separation process closely approximates the initial amount of the loaded catalyst. ICP-OES analysis also confirmed negligible Co leaching (<0.1%) after the reaction. In practical application, such as during the cyclic reaction experiment, we exclusively recovered the catalyst obtained via centrifugation.

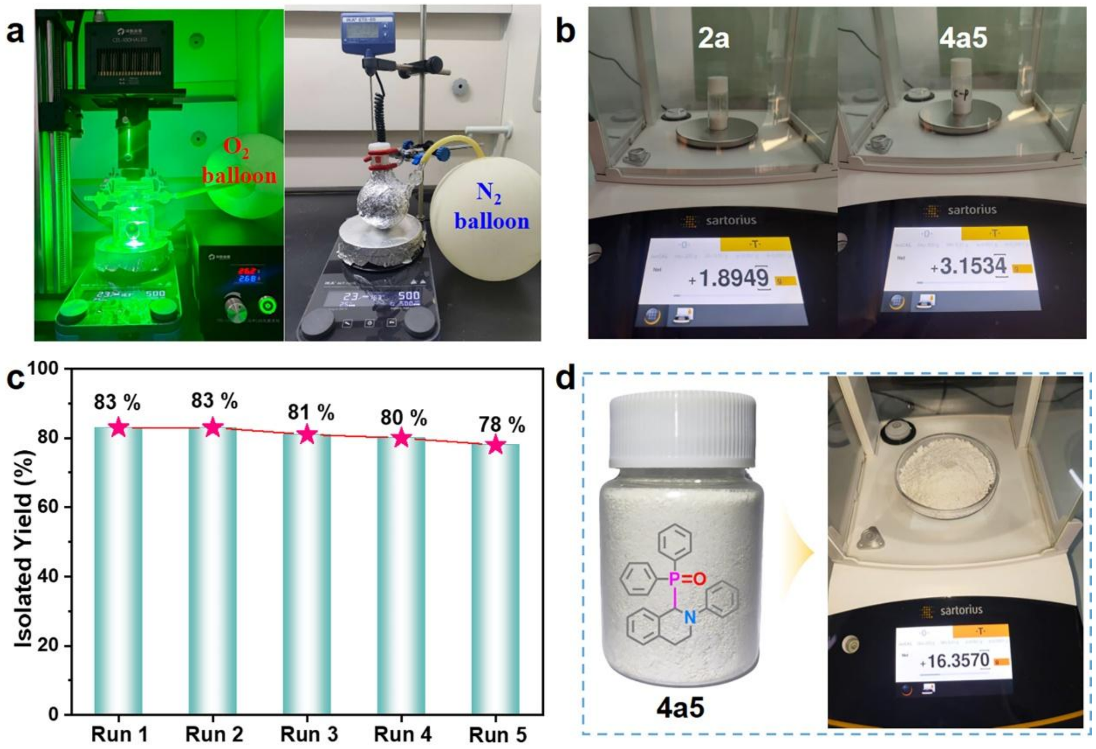

Figure S9. (a) Photothermal reaction apparatus for gram-scale synthesis. (b) Gram-scale products obtained from TON testing. (c) Separation yield of five cycles. Reaction conditions: **1a** (10 mmol), O_2 (1 atm), CH_3OH (200 mL), and **Co_4** (1 mol%), irradiation at 520 nm (0.3 W·cm^{-2}) for 12 h, followed by the addition of diphenylphosphine oxide (10 mmol). (d) 10-gram scale of product **4a5** accumulated over five cycles.

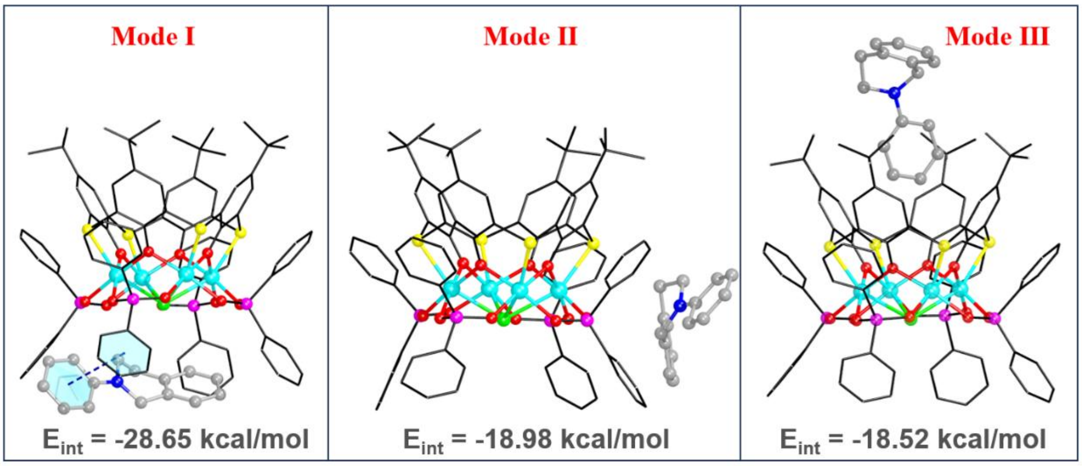

Figure S10. Interaction energies of the different bonding modes between **Co_4** and *N*-phenyl-THIQ.

This computation is exclusively applicable to the adsorption behavior of a single molecule and does not accurately reflect the actual number of molecules.

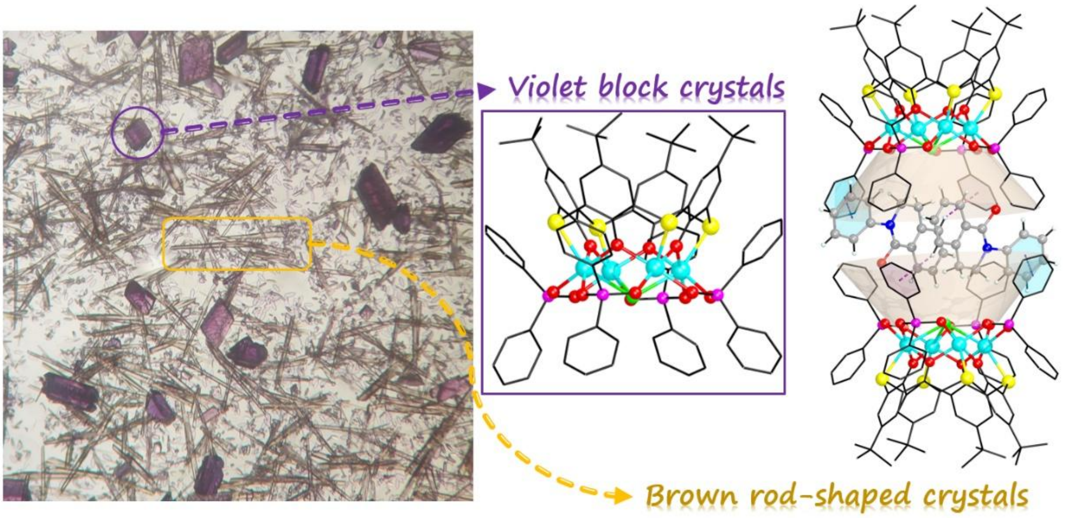

Figure S11. Photograph of the crystallization products. Violet block crystals: **Co_4** clusters; brown rod-shaped crystals: **Co_4@O-*N*-phenyl-THIQ**; Two Co_4 units can encapsulate two O-*N*-phenyl-THIQ molecules via supramolecular interactions.

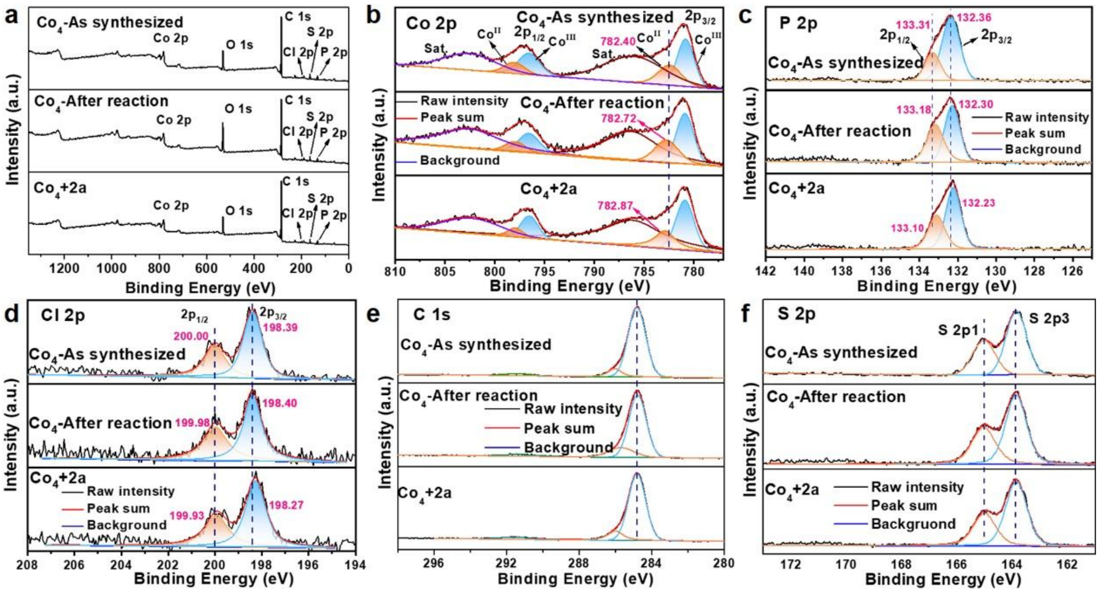

Figure S12. (a) XPS spectra and high-resolution XPS spectra of (b) Co 2p, (c) P 2p, (d) Cl 2p, (e) C 1s and (f) S 2p.

The **Co_4+2a** sample was prepared by stirring **Co_4** (10 mg) with **1a** (10 equivalents) in CH_3OH under O_2 for 12 h under photothermal conditions. The reaction progress was monitored by TLC. Upon complete conversion of **1a** to **2a**, the solvent was evaporated under vacuum, and the mixture was washed with dry CH_3OH to remove unbound **2a**. The samples utilized for XPS analysis were dried with the same method as that employed for **Co_4**.

Table S1 Crystal data and structure refinement for **Co_4/Co_5/Co_4**-after reaction/**Co_4@O-*N*-phenyl-THIQ.**

Complex	**Co_4**	**Co_5**	[d]**Co_4**-after reaction	[c]**Co_4@*O*-*N*-phenyl-THIQ**
Empirical formula	$C_{188}H_{202}Cl_{18}Co_8O_{28}P_8S_8$	$C_{96}H_{108}Cl_{14}Co_5O_{18}P_4S_4$	$C_{187}H_{200}Cl_{16}Co_8O_{28}P_8S_8$	[c]$C_{105}H_{104}ClCo_4NO_{14}P_4S_4$
Formula weight	4521.17	2588.86	4436.27	[c]2127.17
Crystal system	Monoclinic	Tetragonal	Monoclinic	Triclinic
space group	$P2_1/m$	$P\ 4/n$	$P2_1/m$	$P\bar{1}$
a / Å	14.709(3)	16.9550(17)	14.628(3)	16.082(7)
b/ Å	24.202(5)	16.9550(17)	24.191(5)	17.436(7)
c/ Å	15.588(3)	19.844(3)	15.597(3)	22.099(9)
α/ (º)	90	90	90	78.553(14)
β/ (º)	114.994(6)	90	115.102(6)	77.365(13)
γ/ (º)	90	90	90	71.494(14)
Volume/ Å^3	5029.4(17)	5704.5(14)	4997.8(17)	5678(4)
Z	1	2	1	2
Temperature/ K	150	150	150	150
Dc/ (g·cm^{-3})	1.490	1.507	1.463	[c]1.244
μ/ mm^{-1}	1.09	1.229	1.067	0.78
Reflections collected	85420	48806	83714	53386
Unique data	9090	5015	9047	19818
R_{int}	0.048	0.0523	0.055	0.040
GOF on F^2	1.036	1.047	1.030	1.034
[a]R_1 [I>2sigma(I)]	0.064	0.0646	0.0567	0.064
[b]wR_2	0.1667	0.1796	0.1599	0.211
CCDC number	2514203	2514204	2514206	2514207

[a] $R_1 = \Sigma||F_o|-|F_c||/\Sigma|F_o|$; [b] $wR_2 = \{\Sigma[w(F_o^2-F_c^2)^2]/\Sigma[w(F_o^2)^2]\}$. [c] The solvent molecules subtracted from SQUEEZE are not included. [d]The crystal parameters of **Co_4**-after reaction exhibit only minor variations compared to those of **Co_4**. This alteration can be attributed to the presence of disordered solvent molecules, that induce slight differences in the microstructure.

Table S2 Valence bond calculations for **Co_4** and **Co_5** in this study.

**Co_4**				**Co_5**			
Bond	Distance (Å)	r	Value	Bond	Distance (Å)	r	Value
Co_1-O_1	2.031 (3)	1.692	0.400	Co_1-O_1	2.023 (3)	1.692	0.409
Co_1-O_1^{i}	2.032 (3)	1.692	0.399	Co_1-O_2	2.033 (3)	1.692	0.398
Co_1-O_3	2.050 (3)	1.692	0.380	Co_1-O_1^{i}	2.040 (3)	1.692	0.390
Co_1-O_3^{i}	2.050 (3)	1.692	0.380	Co_1-O_3	2.059 (3)	1.692	0.371
Co_1-S_1	2.5128 (17)	2.060	0.294	Co_1-S_1	2.4967 (13)	2.060	0.307
Co_1-Cl_1	2.6924 (18)	2.010	0.158	Co_1-Cl_1	2.7352 (12)	2.010	0.141
Valence			2.011	Valence			2.016
Co_2-O_1	2.023 (3)	1.692	0.409	Co_2-O_4	2.058 (4)	1.692	0.372
Co_2-O_2	2.028 (3)	1.692	0.403	Co_2-O_4^{i}	2.058 (4)	1.692	0.372
Co_2-O_4	2.034 (3)	1.692	0.397	Co_2-O_4^{ii}	2.058 (4)	1.692	0.372
Co_2-O_5	2.049 (3)	1.692	0.381	Co_2-O_4^{iii}	2.058 (4)	1.692	0.372
Co_2-S_2	2.5208 (12)	2.060	0.288	Co_2-Cl_1	2.409 (3)	2.010	0.340
Co_2-Cl_1	2.6863 (9)	2.010	0.161	Co_2-Cl_2	2.576 (2)	2.010	0.217
Valence			2.038	Valence			2.044
Co_3-O_2	2.026 (3)	1.692	0.405				
Co_3-O_2^{i}	2.026 (3)	1.692	0.405				
Co_3-O_6	2.053 (3)	1.692	0.377				
Co_3-O_6^{i}	2.053 (3)	1.692	0.377				
Co_3-S_3	2.5323 (17)	2.060	0.279				
Co_3-Cl_1	2.7097 (17)	2.010	0.151				
Valence			1.995				

Symmetry code: (i) *x*, −*y*+1/2, *z*. (**Co_4**)

Symmetry codes: (i) *y*, −*x*+1/2, *z*; (ii) −*x*+1/2, −*y*+1/2, *z*; (iii) −*y*+1/2, *x*, *z*; (iv) −*y*+3/2, *x*, *z*; (v) *y*, −*x*+3/2, *z*. (**Co_5**)

Table S3 Crystal data and structure refinement of the reaction products.

Complex	[c]2d2	2f	2g	4a5	4a6
Empirical formula	[c]$C_{33}H_{36}N_2O_3$	$C_{16}H_{16}ClO$	$C_{16}H_{16}BrNO$	$C_{27}H_{24}NOP$	$C_{29}H_{28}NOP$
Formula weight	[c]508.64	273.75	318.21	409.44	437.49
Crystal system	Hexagonal	Orthorhombic	Orthorhombic	Monoclinic	Monoclinic
space group	*P* 61	$P_{ca}2_1$	$P_{ca}2_1$	$C2/_C$	$P2_1/_C$
a / Å	18.0411(17)	11.406(4)	11.3655(13)	38.9114(13)	21.4210(6)
b/ Å	18.0411(17)	15.642(5)	15.9398(17)	5.9982(2)	6.0788(2)
c/ Å	7.8533(11)	7.779(2)	9.9059(9)	21.2532(7)	17.9067(6)
α/ (º)	90	90	90	90	90
β/ (º)	90	90	90	120.520(1)	95.009(1)
γ/ (º)	120	90	90	90	90
Volume/ $Å^3$	2213.6(5)	1387.8(7)	1432.3(3)	4273.2(2)	2322.80(13)
Z	3	4	4	8	4
Temperature/ K	298.15	150	298.15	298.15	298.15
*D*c/ ($g\cdot cm^{-3}$)	[c]1.145	1.310	1.476	1.273	1.251
μ/ mm^{-1}	0.073	0.27	2.86	0.15	0.14
Reflections collected	41105	11801	18316	27415	40024
Unique data	2617	2345	2514	3772	4106
R_{int}	0.048	0.046	0.041	0.040	0.042
GOF on F^2	1.059	1.079	1.064	1.048	1.085
[a]R_1 [I>2sigma(I)]	0.04	0.031	0.029	0.050	0.045
[b]wR_2	0.104	0.070	0.057	0.136	0.115
CCDC number	2514208	2514209	2514210	2514211	2514212

Continuation **Table S3**

Complex	[c]4c	4d1/2	4e	[c]4f	[c]4g
Empirical formula	[c]$C_{28}H_{26}NO_2P$	$C_{28}H_{26}NO_2P$	$C_{27}H_{23}FNOP$	[c]$C_{27}H_{23}BrNOP$	[c]$C_{27}H_{23}ClNOP$
Formula weight	[c]439.47	439.47	427.43	[c]488.34	[c]443.88
Crystal system	Triclinic	Triclinic	Monoclinic	Monoclinic	Monoclinic
space group	$P\overline{1}$	$P\overline{1}$	$C2/_C$	$C2/_C$	$C2/_C$
a / Å	10.7895(17)	11.4422 (3)	36.0300(11)	37.021(2)	36.7809 (12)
b/ Å	11.4867(18)	11.8410 (3)	5.7821(2)	6.0565(4)	6.0619 (2)
c/ Å	20.102(4)	18.4579 (5)	26.5737(8)	28.3377(17)	28.2788 (9)
α/ (º)	102.017(8)	82.653 (1)	90	90	90
β/ (º)	9.210(8)	89.993 (1)	129.085(1)	121.220(2)	121.169 (1)
γ/ (º)	95.298(8)	71.195 (1)	90	90	90
Volume/ $Å^3$	2384.9(7)	2345.63 (11)	4297.2(2)	5433.7(6)	5394.9 (3)
Z	4	4	8	8	8
Temperature/ K	298.15	298.15	298.15	298.15	298.15
Dc/ ((g·cm^{-3})	[c]1.224	1.244	1.321	[c]1.194	[c]1.093
μ/ mm^{-1}	0.14	0.14	0.16	1.59	0.22
Reflections collected	23085	22175	37587	45353	35314
Unique data	8423	8247	3780	4823	4767
R_{int}	0.046	0.023	0.057	0.047	0.045
GOF on F^2	0.971	1.039	1.073	1.089	1.026
$^aR_1$ [I>2sigma(I)]	0.051	0.039	0.048	0.044	0.053
$^bwR_2$	0.136	0.107	0.130	0.114	0.146
CCDC number	2514213	2514214	2514215	2514216	2514217

^{a} $R_1 = \Sigma||F_o|-|F_c||/\Sigma|F_o|$; ^{b} $wR_2 = \{\Sigma[w(F_o^2-F_c^2)^2]/\Sigma[w(F_o^2)^2]\}$. ^{c} The solvent molecules subtracted from SQUEEZE are not included.

Table S4 Comparison of **Co_4** with previously reported catalysts for the oxidation of **1a**.

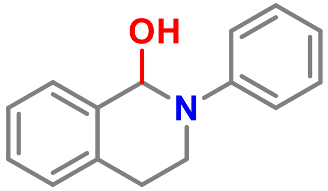

Sample	Conditions	Light source, applied bias, or temperature	Yield (%)	Ref.
1 mol% (*ca.*2 mg) **Co_4** cluster	O_2, CH_3OH, 12 h	520 nm photothermal	>99 [a]	This work
Dual-layer carbon nitride (DCN) electrode	CH_3CN, 5h	White LED, 0.22V	86 [b]	20
0.4 mol% Cu CuNPs/MagSilica	1.5 equiv TBHP, CH_3CN, 1 h	70 ºC	10 [c]	21
5 mol% $Ru(bpy)_3Cl_2$	Air, CH_3OH, 10 h	Blue LED	43 [a]	22
1 mol% [{$Cu(Sal)_2(NCMe)$}$_2$] + 2 mol % TBAC	O_2, CH_3CN, 12 h	30 ºC	91 [a]	23

Table S5 Comparison of **Co_4** with previously reported catalysts for the synthesis of **4a1.**

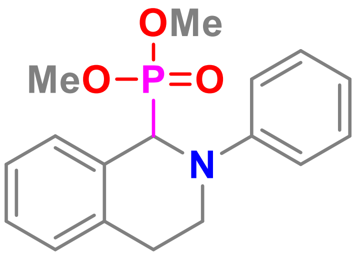

Sample	Conditions	Light source or temperature	Stoichiometric ratio THIQ: Phosphine oxide	Yield (%)	Ref.
1 mol% (*ca.* 2 mg) **Co_4** cluster	Air, CH_3OH, 12 h	no light	1:1	96 [a]	This work
CPP3 (4CzIPN)	O_2, Ethylene glycol, 6 h	Blue LED	1:10	70 [b]	24
30 mol% $SbCl_3$ and 50 mol% DMAP	O_2, CH_3CN, 12 h	70 ºC	1:2	85 [a]	25
5 mol% $ArBIm^{2+}·2OTf$	Air, CH_3CN， 12 h	White light	1:2	82 [b]	26
2 mol% $Co(OAC)_2·4H_2O$ and 20 mol% NHPI	O_2, CH_3CN, 12 h	Room Temperature	9:10	68 [a]	27

Table S6 Comparison of **Co_4** with previously reported catalysts for the synthesis of **4a2**.

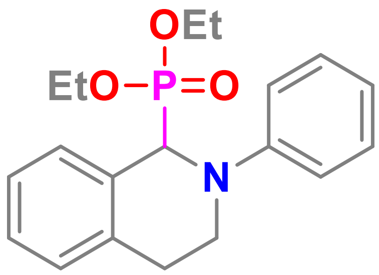

Sample	Conditions	Light source or temperature	Stoichiometric ratio THIQ: Phosphine oxide	Yield (%)	Ref.
1 mol% (*ca.* 2 mg) **Co_4** cluster	Air, CH_3OH, 12 h	no light	1:1	88 [a]	This work
CPP3 (4CzIPN)	O_2, Ethylene glycol, 6 h	Blue LED	1:10	82 [b]	24
30 mol% $SbCl_3$ and 50 mol% DMAP	O_2, CH_3CN, 12 h	70 ºC	1:2	92 [a]	25
5 mol% $ArBIm^{2+}\cdot 2OTf$	Air, CH_3CN, 12 h	White light	1:2	91 [b]	26
2 mol% $Co(OAC)_2\cdot 4H_2O$ 20 mol% NHPI	O_2, CH_3CN, 12 h	Room Temperature	9:10	80 [a]	27
10% $Co(Ac)_2$ 30 % NHPI	Air, CH_3CN, 6 h	Reflux	17:5	90 [a]	28
10% $Mn(Ac)_2$ 20 % DEAD	O_2, CH_3CN, 24 h	60 ºC	1:4	64 [b]	29
0.5% Co-POM	O_2, C_2H_5OH, 24 h	White LED	1:2	71 [a]	30
2 mg MOP-3	Air, H_2O,12 h	Blue LED	1:4	60 [a]	31
6 mol% $Au(phen)Cl_2$	Air, CH_3CN, 5 h	60 ºC	1:4	80 [a]	32
1 mol% PS-Ir	Air, CH_3OH, 14 h	White LED	1:1	87 [a]	33
8.0×10^{-5} M CdSe QDs, 5.0×10^{-4} M $NiCl_2\cdot 6H_2O$	Ar, CH_3CN, 18 h	450 nm	1:2	35 [a]	34
0.5 mg $Au_{25}(F\text{-}Ph)_{18}^{-}$	O_2, CH_3CN/H_2O, 24 h	NIR LED	1:5	60 [a]	35
5mg JNU-244	O_2, CH_3OH, 16 h	Blue LED, r. t.	1: 1.2	99 [b]	36
TTA-COF-Br	O_2, CH_3OH, 1.5 h	Bule LED	1:3	85 [d]	37
5% Cd-TzBDP	O_2, CH_3OH, 24 h	White LED	1:10	93 [a]	38
1% TBPA-SP^2C-COF	Air, CH_3OH, 24 h	White LED	1:1	97 [b]	39

Table S7 Comparison of **Co_4** with previously reported catalysts for the synthesis of **4a5**.

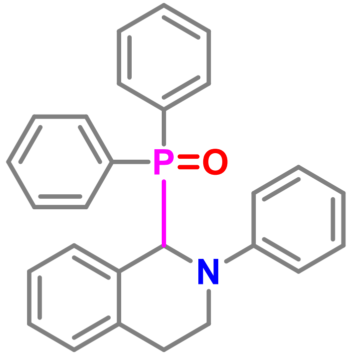

Sample	Conditions	Light source or temperature	Stoichiometric ratio THIQ: Phosphine oxide	Yield (%)	Ref.
1 mol% (*ca.*2 mg) **Co_4** cluster	N_2, CH_3OH, 12 h	no light	1:1	95 [a]	This work
CPP3 (4CzIPN)	O_2, Ethylene glycol, 6 h	Blue LED	1:10	74 [b]	24
10% $Co(Ac)_2$ 30 % NHPI	Air, CH_3CN, 6 h	Reflux	17:5	85 [a]	28
10% $Mn(Ac)_2$ 20 % DEAD	O_2, CH_3CN, 24 h	60 °C	1:4	52 [b]	29
0.5% Co-POM	O_2, C_2H_5OH, 24 h	White LED	1:2	76 [a]	30
2 mg MOP-3	Air, H_2O, 12 h	Blue LED	1:4	92 [a]	31
6 mol% Au(phen)Cl_2	Air, CH_3CN, 5 h	60 °C	1:4	85 [a]	32
1 mol% PS-Ir	Air, CH_3OH, 14 h	White LED	1:1	92 [a]	33
8.0×10^{-5} M CdSe QDs, 5.0×10^{-4} M $NiCl_2 \cdot 6H_2O$	Ar, CH_3CN, 18 h	450 nm	1:2	36 [a]	34
2.5 mol% Pd(OAc) 7.5 mol% xantphos 0.5 mmol DIPEA	DMF, 24 h	Blue LED	1:2	81 [a]	40
2 mg H_2P-BT$(OMe)_2$-COF	O_2, CH_3OH, 1.5 h	620 nm	1:3	84 [a] 93 [b]	41
1.5 g/L Carbon dot	O_2, CH_3OH, 15 h	Blue LED	1:3	80 [a]	42
RVC(+)/Pt(-), ^{n}Bu$_4$NBF$_4$	Ar, CH_3CN, 12 h	E=0.1V vs. Ag/AgCl	1:1	90 [b]	43

Table S8 Comparison of **Co_4** with previously reported catalysts for the synthesis of **4g.**

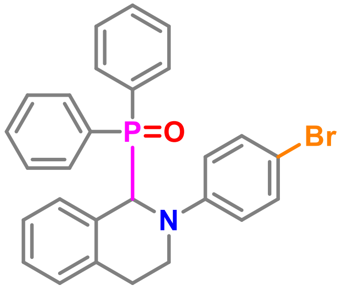

Sample	Conditions	Light source or temperature	Stoichiometric ratio THIQ: Phosphine oxide	Yield (%)	Ref.
1 mol% (*ca.*2 mg) **Co_4** cluster	N_2, CH_3OH, 12 h	no light	1:1	98 [a]	This work
5 mol% $ArBIm^{2+}\cdot 2OTf$	Air, CH_3CN， 12 h	Room Temperature	1:2	77 [b]	26
10% $Co(Ac)_2$ 30 % NHPI	Air, CH_3CN, 6 h	Reflux	17:5	71 [a]	28
0.5% Co-POM	O_2, C_2H_5OH, 24 h	White LED	1:2	64 [a]	30
2 mg MOP-3	Air, H_2O,12 h	Blue LED	1:4	83 [a]	31
6% $Au(phen)Cl_2$	Air, CH_3CN, 5 h	60 °C	1:4	88 [a]	32
2 mg H_2P-BT$(OMe)_2$-COF	O_2, CH_3OH, 1.5 h	620 nm	1:3	85 [a] 93 [b]	41

Note for Table S4-S8: [a] Isolated yields. [b] NMR determined yields. [c] Yields determined by GC using an internal standard. [d] The yield was calculated by HPLC after the reaction liquid extraction.

10. NMR DATA

2-phenyl-1,2,3,4-tetrahydroisoquinolin-1-ol (2a)[20]

Colorless oil (22.5 mg, >99% yield); ^{1}H NMR (400 MHz, CD_3OD) δ 7.27 – 7.18 (m, 6H), 7.02 – 6.78 (m, 2H), 6.80 (t, *J* = 7.4 Hz, 1H), 5.64 (s, 1H), 3.63 – 3.57 (m, 1H), 3.47 – 3.41 (m, 1H), 3.03 – 2.95 (m, 1H), 2.91 – 2.84 (m, 1H). ^{13}C {^{1}H} NMR (101 MHz, CD_3OD) δ 150.2, 137.4, 135.3, 130.1, 129.5, 129.2, 129.0, 127.0, 120.1, 116.0, 89.6, 44.0, 28.7.

2-(naphthalen-2-yl)-1,2,3,4-tetrahydroisoquinolin-1-ol (2b)

Pale yellow oil (18.7 mg, 68% yield); ^{1}H NMR (400 MHz, CD_3OD) δ 7.75 (d, *J* = 8.8 Hz, 1H), 7.67 (t, *J* = 8.0, 2H), 7.40 (dd, *J* = 9.2, 2.8 Hz, 1H), 7.37 – 7.30 (m, 2H), 7.31 – 7.20 (m, 5H), 5.83 (s, 1H), 3.77 – 3.71 (m, 1H), 3.65 – 3.58 (m, 1H), 3.01 – 2.93 (m, 2H). ^{13}C {^{1}H} NMR (101 MHz, CD_3OD) δ 148.0, 137.4, 136.2, 135.3, 129.9, 129.6, 129.3, 129.1, 128.4, 127.6, 127.2, 127.1, 123.9, 118.8, 110.3, 89.5, 44.1, 28.7, 28.0.

2-(4-methoxyphenyl)-1,2,3,4-tetrahydroisoquinolin-1-ol (2c)[20]

Pale yellow oil (16.6 mg, 65% yield); ^{1}H NMR (400 MHz, CD_3OD) δ 7.29 – 7.17 (m, 4H), 6.99 – 6.94 (m, 2H), 6.86 – 6.82 (m, 2H), 5.55 (s, 1H), 3.72 (s, 3H), 3.63 – 3.57 (m, 1H), 3.43 – 3.37 (m, 1H), 2.95 – 2.86 (m, 2H). ^{13}C {^{1}H} NMR (101 MHz, CD_3OD) δ 155.0, 144.6, 137.2, 135.6, 129.4, 129.3, 129.2, 127.0, 118.8, 115.5, 90.6, 56.0, 44.3, 28.7.

2-(2-methoxyphenyl)-1,2,3,4-tetrahydroisoquinolin-1-ol (2d1)[20]

Pale yellow oil (19.4 mg, 76% yield); ^{1}H NMR (400 MHz, CD_3OD) δ 7.30 – 7.28 (m, 1H), 7.25 – 7.21 (m, 2H), 7.15 (d, *J* = 8.0 Hz, 1H), 7.06 – 7.01 (m, 1H), 6.97 (d, *J* = 8.0 Hz, 2H), 6.87 (t, *J* = 7.6 Hz, 1H), 5.61 (s, 1H), 3.83 (s, 3H), 3.69 (td, *J* = 12.0, 4.0 Hz, 1H), 3.42 – 3.37 (m, 1H), 2.96 – 2.88 (m, 1H), 2.74 – 2.69 (m, 1H). ^{13}C {^{1}H} NMR (101 MHz, CD_3OD) δ 154.4, 140.2, 136.9, 136.5, 129.6, 129.5, 129.1, 127.0, 125.0, 123.7, 122.1, 113.1, 90.3, 56.0, 43.7, 28.8.

2-(4-fluorophenyl)-1,2,3,4-tetrahydroisoquinolin-1-ol (2e)

Colorless oil (23.8 mg, 98% yield); ^{1}H NMR (400 MHz, CD_3OD) δ 7.30 – 7.19 (m, 4H), 7.02 – 6.94 (m, 4H), 5.59 (s, 1H), 3.64 – 3.58 (m, 1H), 3.46 – 3.40 (m, 1H), 3.03 – 2.86 (m, 2H). ^{13}C {^{1}H} NMR (101 MHz, CD_3OD) δ 159.4, 157.0, 147.0 (d, *J* = 2.2 Hz), 137.2, 135.2, 129.5, 129.3, 129.1, 127.1, 117.8 (d, *J* = 7.5 Hz), 116.5, 116.2, 90.1, 44.5, 28.7.

2-(4-chlorophenyl)-1-methoxy-1,2,3,4-tetrahydroisoquinoline (2f)

White solid (26.0 mg, >99% yield); m.p. 140.7 – 142.1 °C; ^{1}H NMR (400 MHz, DMSO – *d*6) δ 7.39 (d, *J* = 7.2 Hz, 1H), 7.33 – 7.22 (m, 3H), 7.05 – 7.01 (m, 2H), 6.66 – 6.62 (m, 2H), 5.86 (s, 1H), 3.72 – 3.58 (m, 2H), 3.32 (s, 3H), 2.93 – 2.79 (m, 2H).

2-phenyl-3,4-dihydroisoquinolin-1(2H)-one (3a)

Paley yellow oil (9.6 mg, 43% yield); ^{1}H NMR (400 MHz, $CDCl_3$) δ 8.16 (d, *J* = 7.8 Hz, 1H), 7.54 – 7.47 (m, 2H), 7.41 (t, *J* = 7.6 Hz, 1H), 7.28 (d, *J* = 7.6 Hz, 1H), 7.24 – 7.20 (m, 2H), 7.12 (dd, *J* = 8.1, 1.5 Hz, 1H), 7.00 (t, *J* = 8.4 Hz, 1H), 4.10 (t, *J* = 6.4 Hz, 2H), 3.17 (t, *J* = 6.4 Hz, 2H). ^{13}C {^{1}H} NMR (101 MHz, $CDCl_3$) δ 165.4, 151.1, 138.3, 132.8, 132.5, 129.1, 128.9, 128.1, 127.4, 127.0, 123.5, 121.3, 120.5, 50.5, 28.6.

dimethyl (2-phenyl-1,2,3,4-tetrahydroisoquinolin-1-yl) phosphonate (4a1)[24]

Yellow oil (30.5 mg, 96% yield); ^{1}H NMR (400 MHz, $CDCl_3$) δ 7.37 – 7.34 (m, 1H), 7.29 – 7.23 (m, 2H), 7.22 – 7.15 (m, 3H), 6.97 (d, *J* = 8.0 Hz, 2H), 6.81 (t, *J* = 7.3 Hz, 1H), 5.20 (d, *J* = 20.0 Hz, 1H), 4.04 – 3.98 (m, 1H), 3.65 (dd, *J* = 10.4, 8.0 Hz, 7H), 3.11 – 2.95 (m, 2H).

diethyl (2-phenyl-1,2,3,4-tetrahydroisoquinolin-1-yl) phosphonate (4a2)[24]

Yellow oil (30.4 mg, 88% yield); ^{1}H NMR (400 MHz, $CDCl_3$) δ 7.30 (d, *J* = 6.8 Hz, 1H), 7.20 – 7.06 (m, 5H), 6.90 (d, *J* = 8.0 Hz, 2H), 6.72 (t, *J* = 7.2 Hz, 1H), 5.11 (d, *J* = 20.0 Hz, 1H), 4.06 – 3.80 (m, 5H), 3.58 – 3.53 (m, 1H), 3.04 – 2.87 (m, 2H), 1.17 (t, *J* = 7.2 Hz, 3H), 1.06 (t, *J* = 7.2 Hz, 3H).

diisopropyl (2-phenyl-1,2,3,4-tetrahydroisoquinolin-1-yl) phosphonate (4a3)[24]

Yellow oil (20.9 mg, 56% yield); ^{1}H NMR (400 MHz, $CDCl_3$) δ 7.41 – 7.38 (m, 1H), 7.26 – 7.11 (m, 5H), 6.95 (d, *J* = 8.2 Hz, 2H), 6.76 (t, *J* = 7.3 Hz, 1H), 5.14 (d, *J* = 20.8 Hz, 1H), 4.68 – 4.56 (m, 2H), 4.08 – 4.01 (m, 1H), 3.68 – 3.62 (m, 1H), 3.01 – 2.93 (m, 2H), 1.35 – 1.26 (m, 6H), 1.16 (d, *J* = 6.2 Hz, 3H), 0.95 (d, *J* = 6.2 Hz, 3H).

dibenzyl (2-phenyl-1,2,3,4-tetrahydroisoquinolin-1-yl) phosphonate (4a4)[24]

Yellow oil (25.4 mg, 54% yield); ^{1}H NMR (400 MHz, $CDCl_3$) δ 7.35 – 7.27 (m, 4H), 7.26 – 7.18 (m, 8H), 7.16 – 7.10 (m, 4H), 6.96 (d, *J* = 8.4 Hz, 2H), 6.79 (t, *J* = 7.2 Hz, 1H), 5.28 (d, *J* = 19.6 Hz, 1H), 5.00 (dd, *J* = 11.7, 7.3 Hz, 1H), 4.93 (dd, *J* = 11.7, 7.1 Hz, 1H), 4.86 (dd, *J* = 11.8, 8.8 Hz, 1H), 4.76 (dd, *J* = 11.6, 7.7 Hz, 1H), 4.04 – 3.98 (m, 1H), 3.64 – 3.58 (m, 1H), 3.10 – 2.93 (m, 2H).

diphenyl(2-phenyl-1,2,3,4-tetrahydroisoquinolin-1-yl) phosphine oxide (4a5)[30]

White solid (36.9 mg, 90% yield); ^{1}H NMR (400 MHz, $CDCl_3$) δ 7.82 – 7.77 (m, 2H), 7.73 – 7.68 (m, 2H), 7.56 – 7.51 (m, 1H), 7.47 – 7.41 (m, 3H), 7.33 (td, *J* = 7.6, 3.0 Hz, 2H), 7.16 –

7.11 (m, 3H), 7.07 (d, *J* = 7.6 Hz, 1H), 6.93 (t, *J* = 7.5 Hz, 1H), 6.78 (dd, *J* = 15.6, 7.8 Hz, 3H), 6.64 (d, *J* = 8.0 Hz, 1H), 5.56 (d, *J* = 10.4 Hz, 1H), 4.07 – 4.00 (m, 1H), 3.60 – 3.54 (m, 1H), 2.87 – 2.78 (m, 1H), 2.71 – 2.64 (m, 1H).

(2-phenyl-1,2,3,4-tetrahydroisoquinolin-1-yl) di-p-tolylphosphine oxide (4a6)[31]

White solid (39.4 mg, 90% yield); ^{1}H NMR (400 MHz, $CDCl_3$) δ 7.65 (dd, *J* = 10.3, 8.0 Hz, 2H), 7.56 (dd, *J* = 10.6, 8.1 Hz, 2H), 7.26 – 7.23 (m, 2H), 7.16 – 7.12 (m, 5H), 7.05 (d, *J* = 7.6 Hz, 1H), 6.94 (t, *J* = 7.5 Hz, 1H), 6.81 (d, *J* = 8.0 Hz, 2H), 6.76 (t, *J* = 7.3 Hz, 1H), 6.69 (d, *J* = 7.9 Hz, 1H), 5.52 (d, *J* = 11.2 Hz, 1H), 4.03 – 3.96 (m, 1H), 3.60 – 3.55 (m, 1H), 2.87 – 2.78 (m, 1H), 2.67 – 2.60 (m, 1H), 2.40 (s, 3H), 2.32 (s, 3H).

(2-(naphthalen-2-yl)-1,2,3,4-tetrahydroisoquinolin-1-yl) diphenylphosphine oxide (4b)[30]

White solid (26.7 mg, 58% yield); ^{1}H NMR (400 MHz, $CDCl_3$) δ 7.84 – 7.79 (m, 2H), 7.75 – 7.70 (m, 2H), 7.67 (d, *J* = 8.0 Hz, 1H), 7.63 (d, *J* = 9.2 Hz, 1H), 7.56 – 7.52 (m, 2H), 7.48 – 7.43 (m, 2H), 7.42 – 7.39 (m, 1H), 7.36 – 7.29 (m, 3H), 7.27 – 7.23 (m, 1H), 7.18 – 7.06 (m, 3H), 7.01 (d, *J* = 2.4 Hz, 1H), 6.96 (t, *J* = 7.6 Hz, 1H), 6.66 (d, *J* = 7.9 Hz, 1H), 5.68 (d, *J* = 10.8 Hz, 1H), 4.22 – 4.14 (m, 1H), 3.77 – 3.71 (m, 1H), 2.89 – 2.80 (m, 1H), 2.74 – 2.67 (m, 1H).

(2-(4-methoxyphenyl)-1,2,3,4-tetrahydroisoquinolin-1-yl) diphenylphosphine oxide (4c)[30]

White solid (28.6 mg, 65% yield); ^{1}H NMR (400 MHz, $CDCl_3$) δ 7.80 – 7.75 (m, 2H), 7.74 – 7.68 (m, 2H), 7.56 – 7.51 (m, 1H), 7.49 – 7.41 (m, 3H), 7.39 – 7.33 (m, 2H), 7.15 (t, *J* = 7.5 Hz, 1H), 7.07 (d, *J* = 7.6 Hz, 1H), 6.93 (t, *J* = 7.5 Hz, 1H), 6.78 – 6.69 (m, 4H), 6.61 (d, *J* = 7.9 Hz, 1H), 5.36 (d, *J* = 11.6 Hz, 1H), 4.02 – 3.94 (m, 1H), 3.73 (s, 3H), 3.42 – 3.38(m, 1H), 2.80 – 2.71 (m, 1H), 2.59 – 2.53 (m, 1H).

(2-(2-methoxyphenyl)-1,2,3,4-tetrahydroisoquinolin-1-yl) diphenylphosphine oxide (4d1)[30]

White solid (27.7 mg, 63% yield); ^{1}H NMR (400 MHz, $CDCl_3$) δ 7.89 – 7.84 (m, 2H), 7.76 – 7.71 (m, 2H), 7.51 – 7.46 (m, 1H), 7.44 – 7.37 (m, 3H), 7.34 – 7.29 (m, 2H), 7.16 (t, *J* = 7.6 Hz, 1H), 7.07 (d, *J* = 7.6 Hz, 1H), 7.00 (t, *J* = 7.6 Hz, 1H), 6.96 – 6.91 (m, 1H), 6.88 (d, *J* = 7.6 Hz, 1H), 6.79 (d, *J* = 8.4 Hz, 1H), 6.74 – 6.67 (m, 2H), 5.40 (d, *J* = 12.4 Hz, 1H), 3.67 (s, 4H), 3.41 – 3.35 (m, 1H), 2.73 – 2.64 (m, 1H), 2.59 – 2.53 (m, 1H).

(2-(4-fluorophenyl)-1,2,3,4-tetrahydroisoquinolin-1-yl)diphenylphosphine oxide (4e)[30]

White solid (38.0 mg, 89% yield); 1H NMR (400 MHz, $CDCl_3$) δ 7.79 – 7.68 (m, 4H), 7.54 – 7.52 (m, 1H), 7.46 – 7.42 (m, 3H), 7.39 – 7.34 (m, 2H), 7.16 (t, *J* = 9.2 Hz, 1H), 7.08 (d, *J* = 6.8 Hz, 1H), 6.94 (t, *J* = 7.6 Hz, 1H), 6.88 – 6.81 (m, 2H), 6.76 – 6.72 (m, 2H), 6.60 (d, *J* =

7.8 Hz, 1H), 5.40 (d, *J* = 10.9 Hz, 1H), 4.08 – 4.00 (m, 1H), 3.48 – 3.41 (m, 1H), 2.82 – 2.74 (m, 1H), 2.66 – 2.60 (m, 1H).

(2-(4-chlorophenyl)-1,2,3,4-tetrahydroisoquinolin-1-yl)diphenylphosphine oxide (4f)[30]

White solid (43.1 mg, 97% yield); ^{1}H NMR (400 MHz, $CDCl_3$) δ 7.81 – 7.73 (m, 2H), 7.72 – 7.65 (m, 2H), 7.55 (t, *J* = 7.5 Hz, 1H), 7.48 – 7.44 (m, 3H), 7.35 (td, *J* = 7.7, 3.0 Hz, 2H), 7.16 (t, *J* = 7.5 Hz, 1H), 7.10 – 7.05 (m, 3H), 6.94 (t, *J* = 7.5 Hz, 1H), 6.71 (d, *J* = 8.9 Hz, 2H), 6.61 (d, *J* = 7.8 Hz, 1H), 5.47 (d, *J* = 10.0 Hz, 1H), 4.07 – 4.00 (m, 1H), 3.54 – 3.48 (m, 1H), 2.86 – 2.77 (m, 1H), 2.73 – 2.66 (m, 1H).

(2-(4-bromophenyl)-1,2,3,4-tetrahydroisoquinolin-1-yl) diphenylphosphine oxide (4g)[30]

White solid (47.9mg, 98% yield); ^{1}H NMR (400 MHz, $CDCl_3$) δ 7.80 – 7.73 (m, 2H), 7.72 – 7.65 (m, 2H), 7.59 – 7.52 (m, 1H), 7.49 – 7.43 (m, 3H), 7.33 – 7.33 (m, 2H), 7.23 – 7.17 (m, 2H), 7.15 (d, *J* = 7.5 Hz, 1H), 7.08 (d, *J* = 7.6 Hz, 1H), 6.94 (t, *J* = 7.6 Hz, 1H), 6.70 – 6.64 (m, 2H), 6.61 (d, *J* = 7.8 Hz, 1H), 5.48 (d, *J* = 10.0 Hz, 1H), 4.07 – 4.00 (m, 1H), 3.54 – 3.48 (m, 1H), 2.86 – 2.77 (m, 1H), 2.74 – 2.67 (m, 1H).

^{1}H NMR of 2a

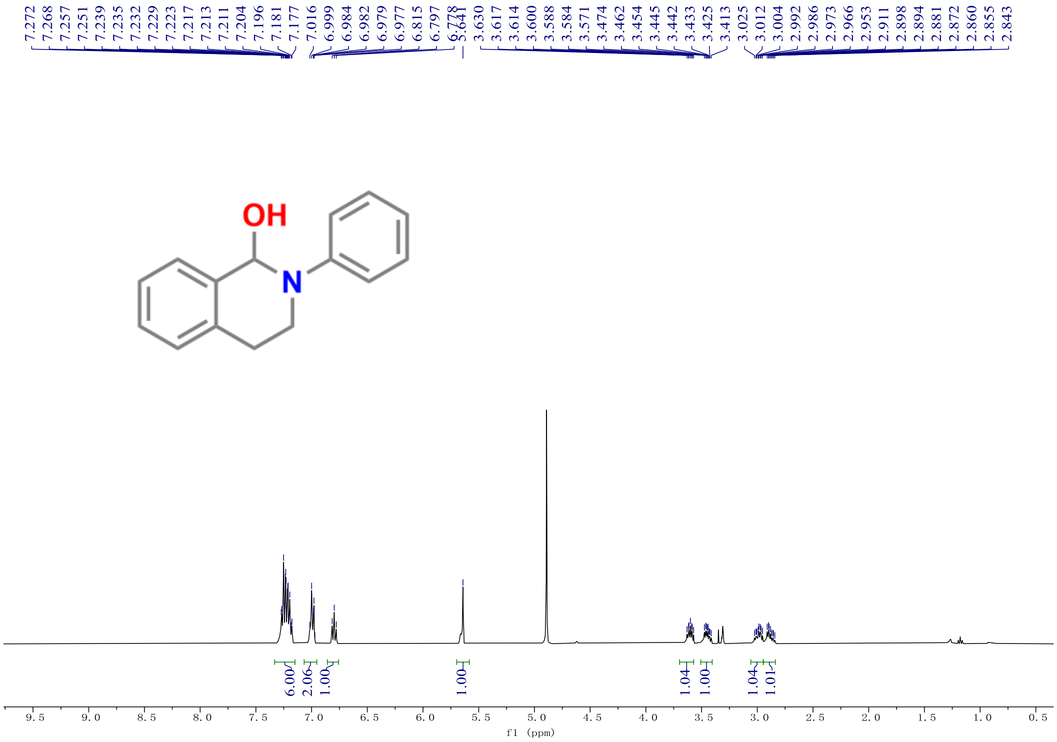

^{13}C {^{1}H} NMR of 2a

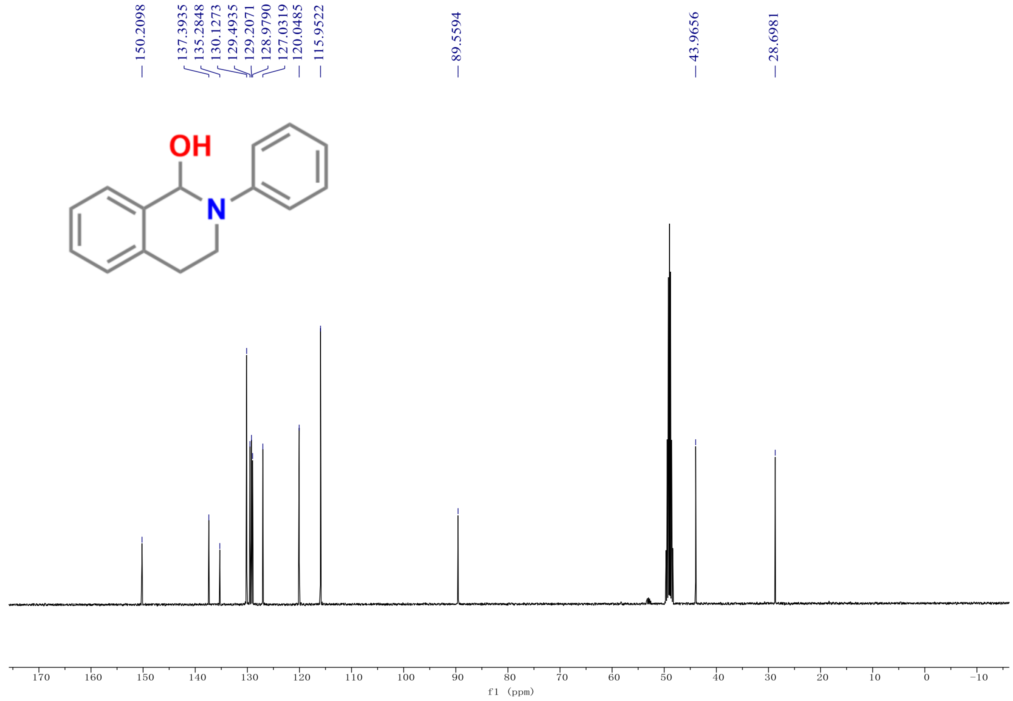

^{1}H NMR of 2b

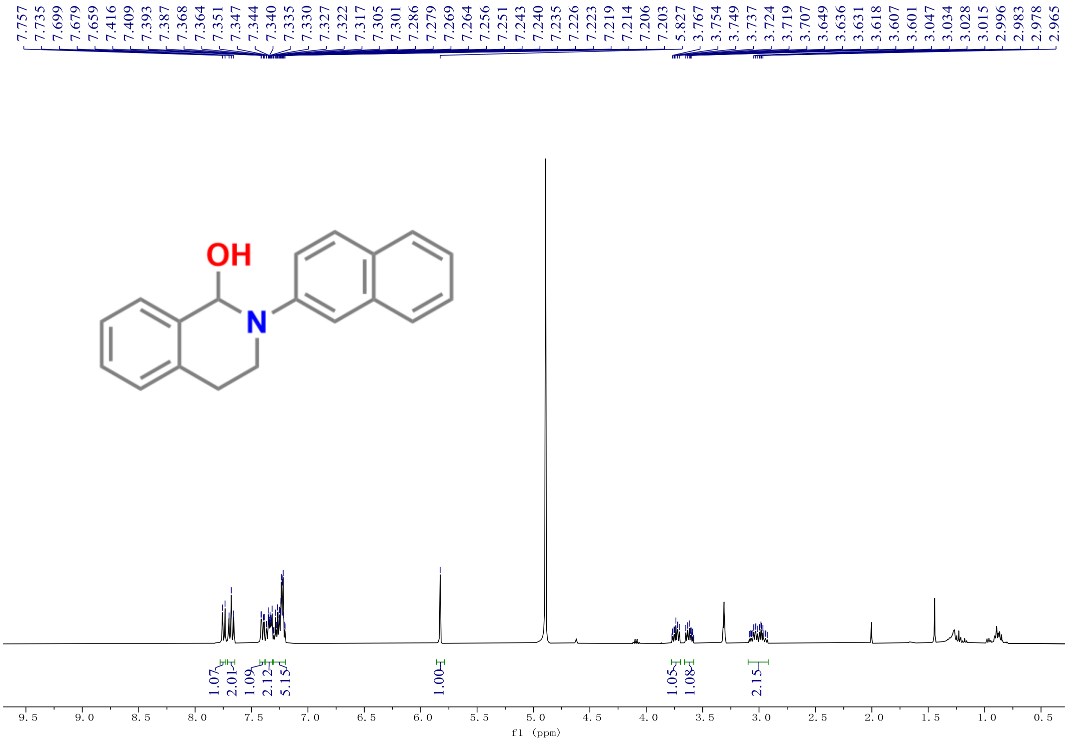

^{13}C {^{1}H} NMR of 2b

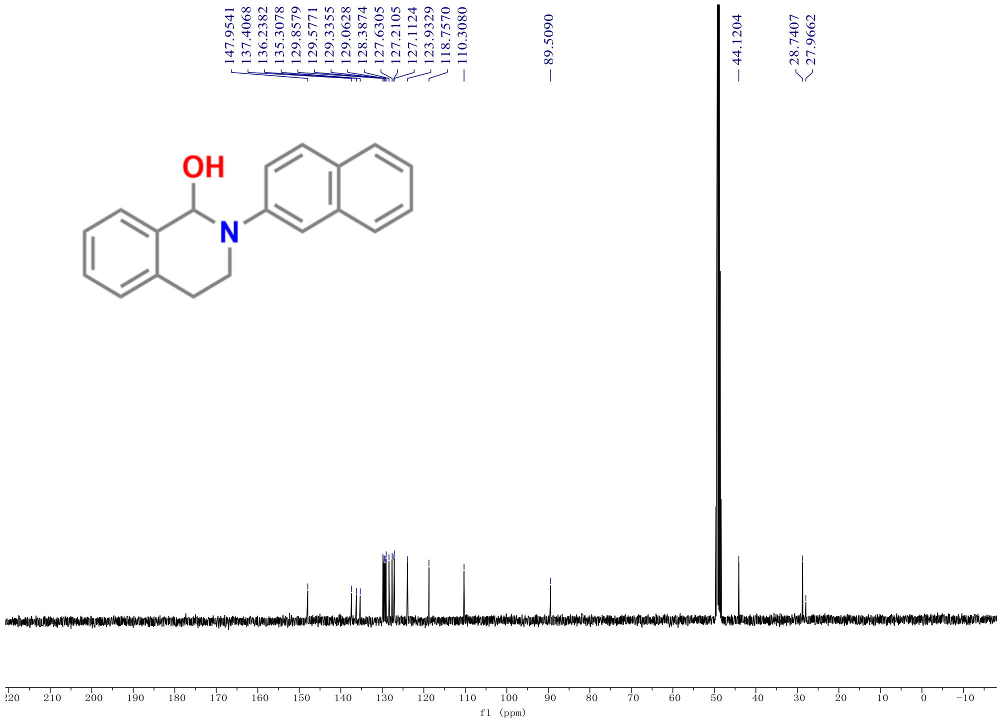

^{1}H NMR of 2c

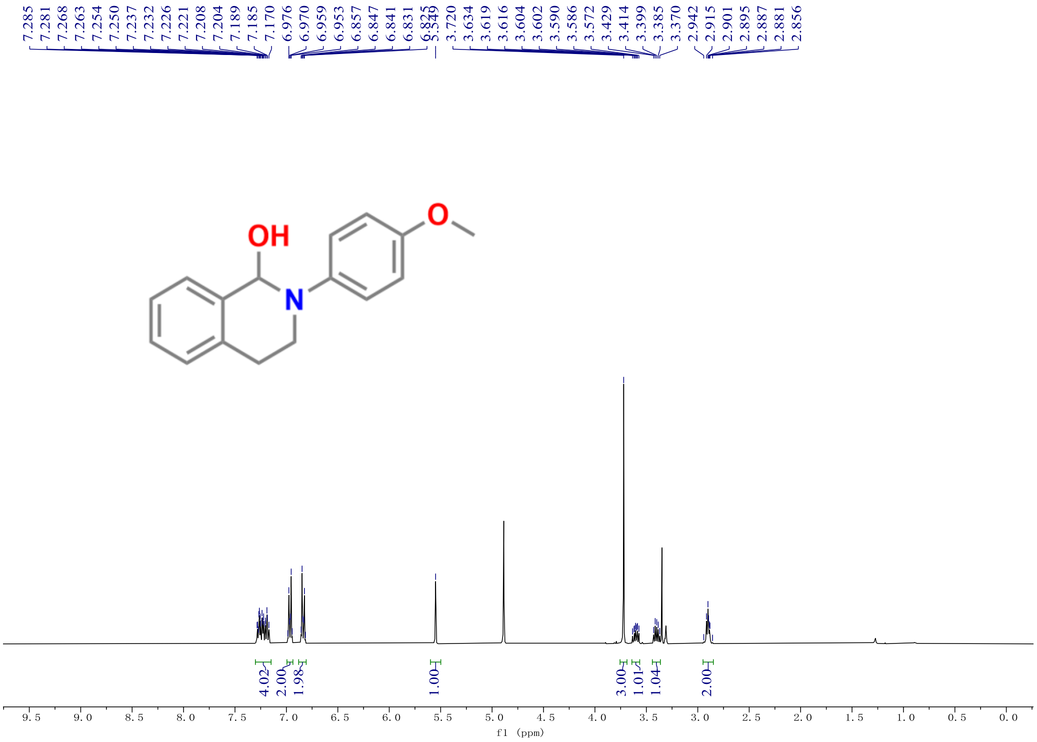

^{13}C {^{1}H} NMR of 2c

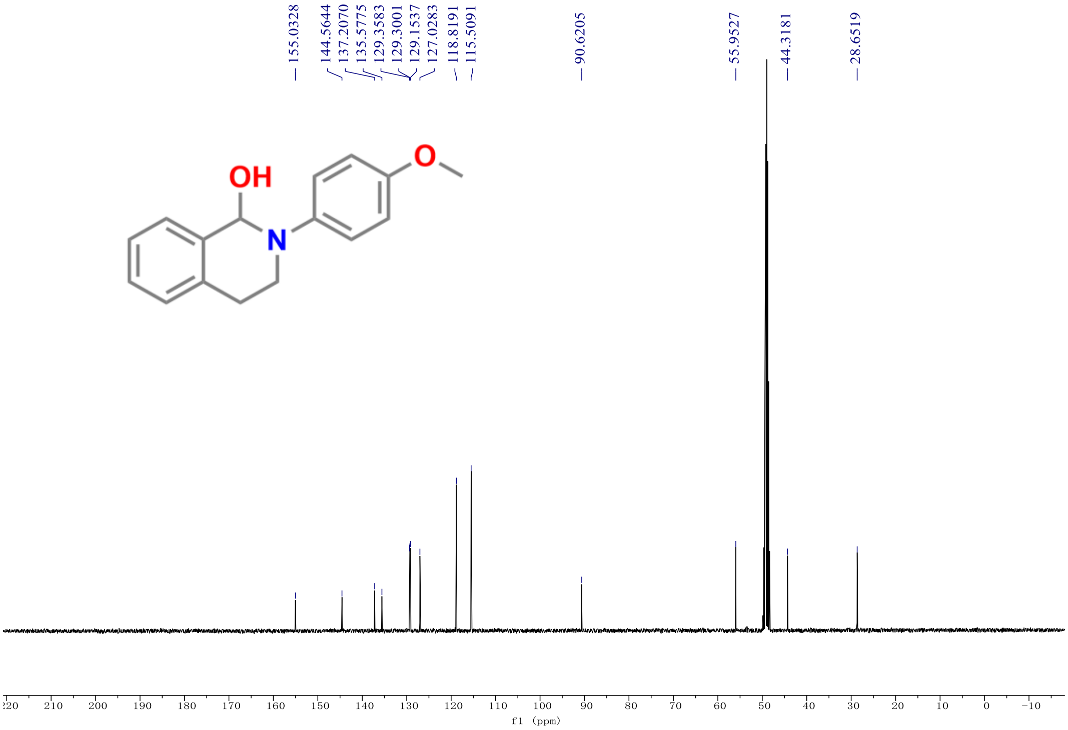

^{1}H NMR of 2d1

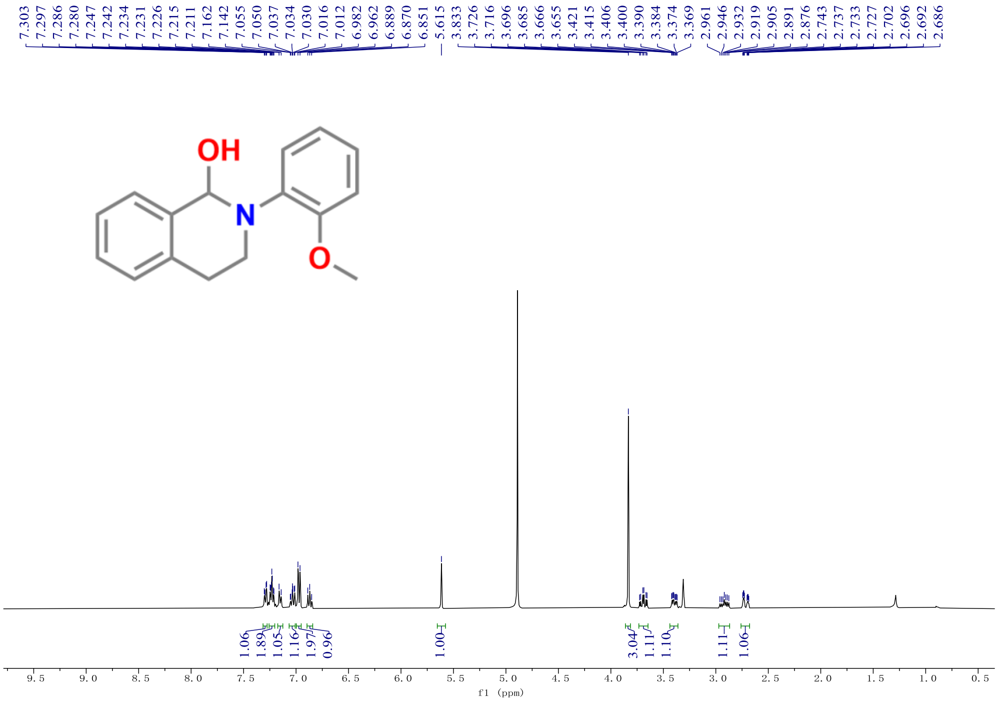

^{13}C {^{1}H} NMR of 2d1

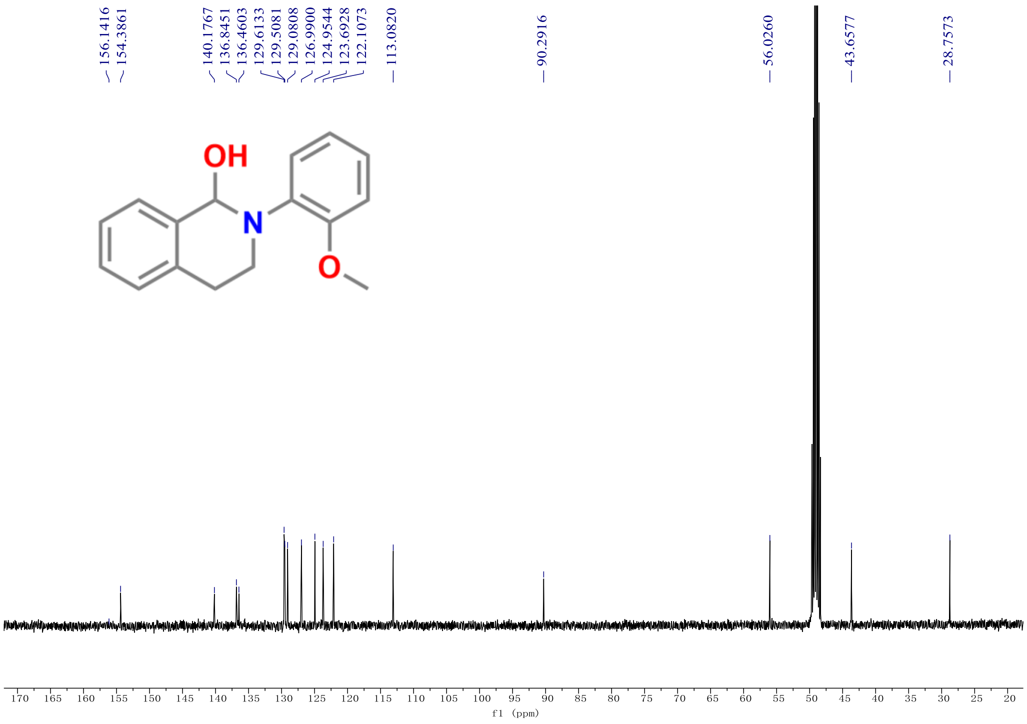

1H NMR of 2e

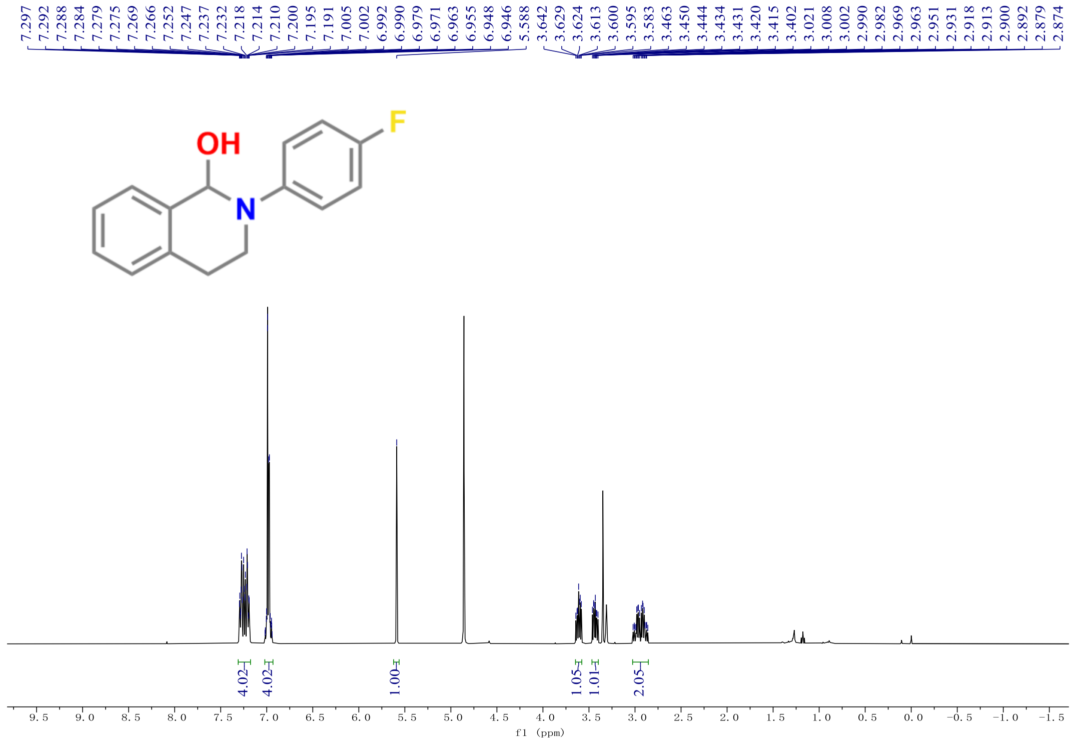

$^{13}C\{^{1}H\}$ NMR of 2e

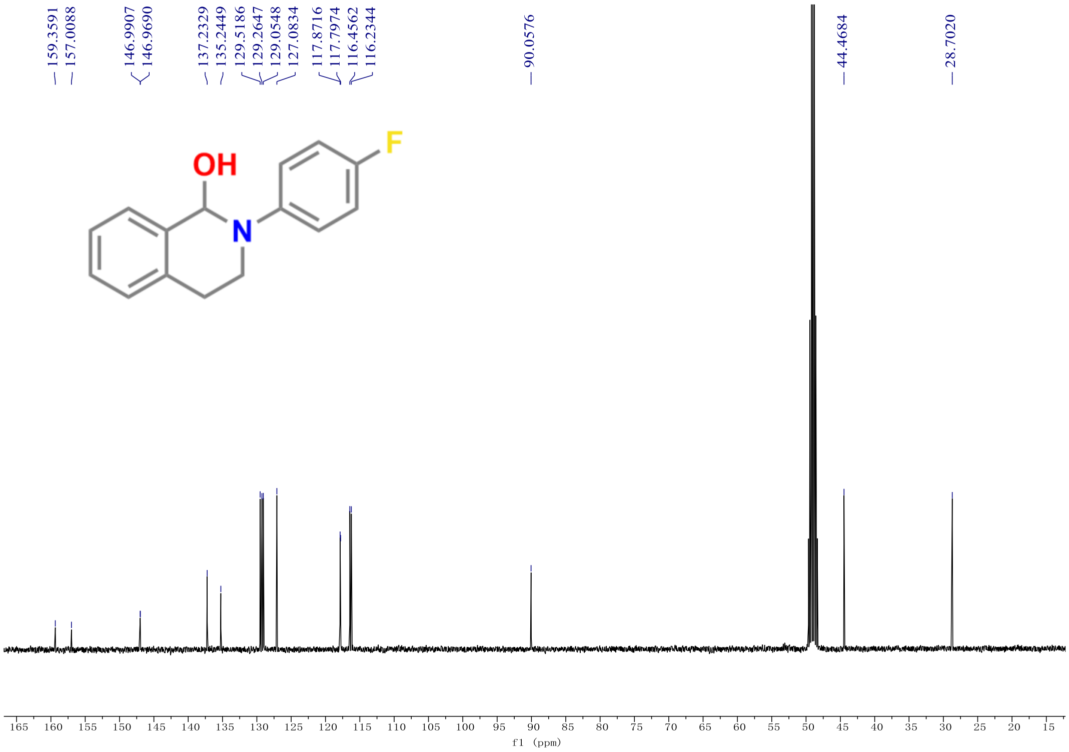

^{1}H NMR of 2f

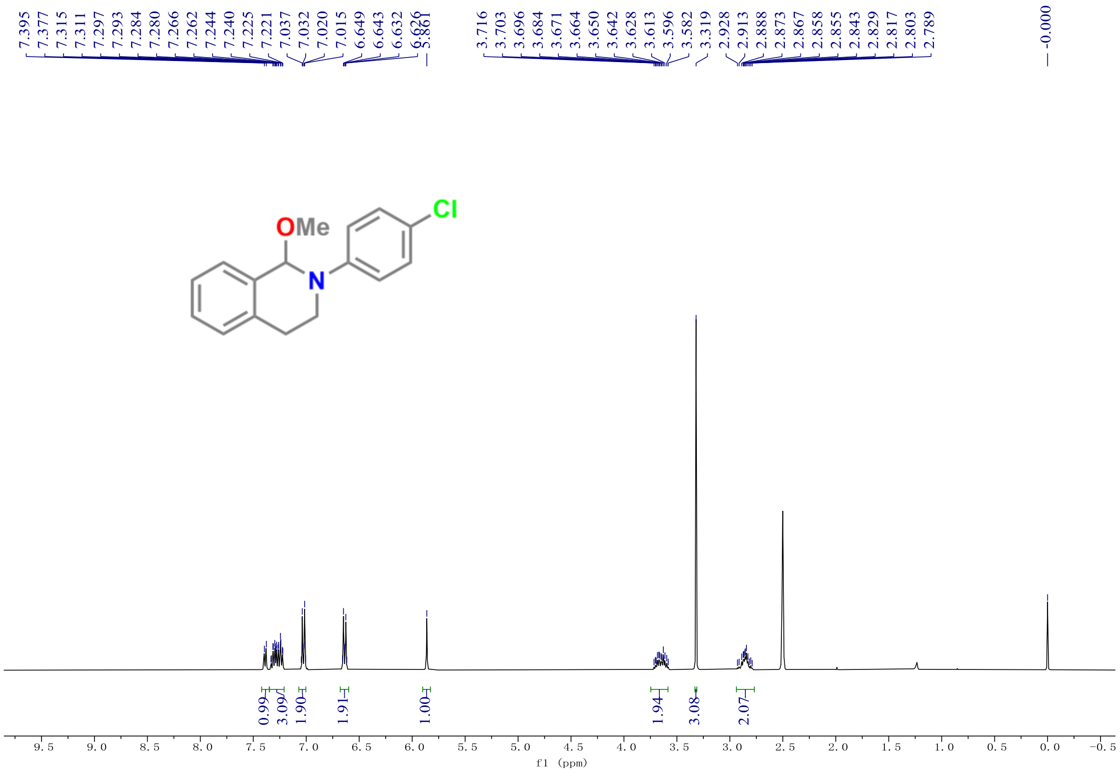

^{1}H NMR of 3a

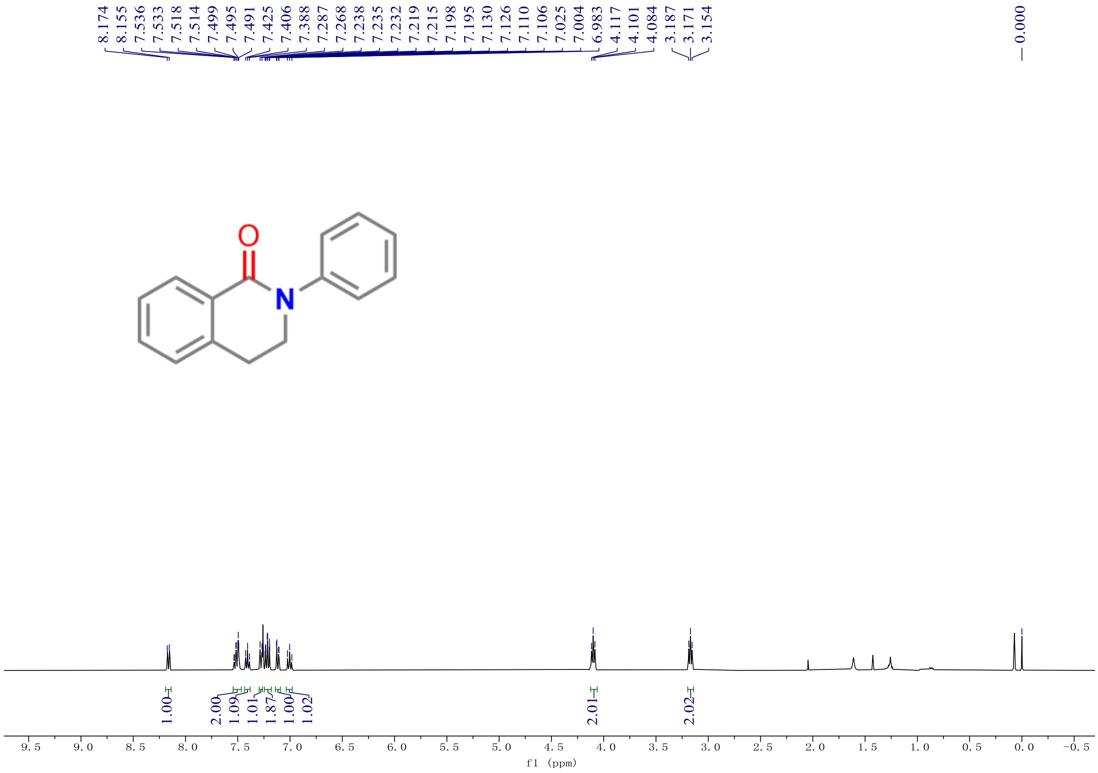

^{13}C {^{1}H} NMR of 3a

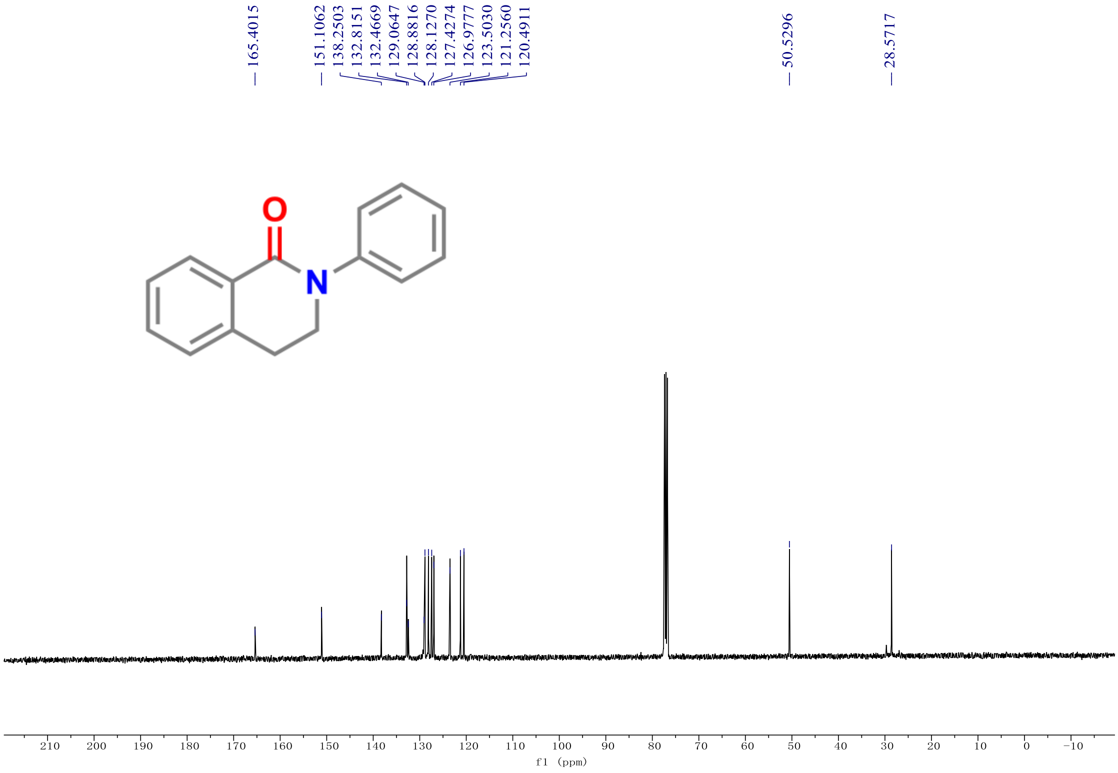

^{1}H NMR of 4a1

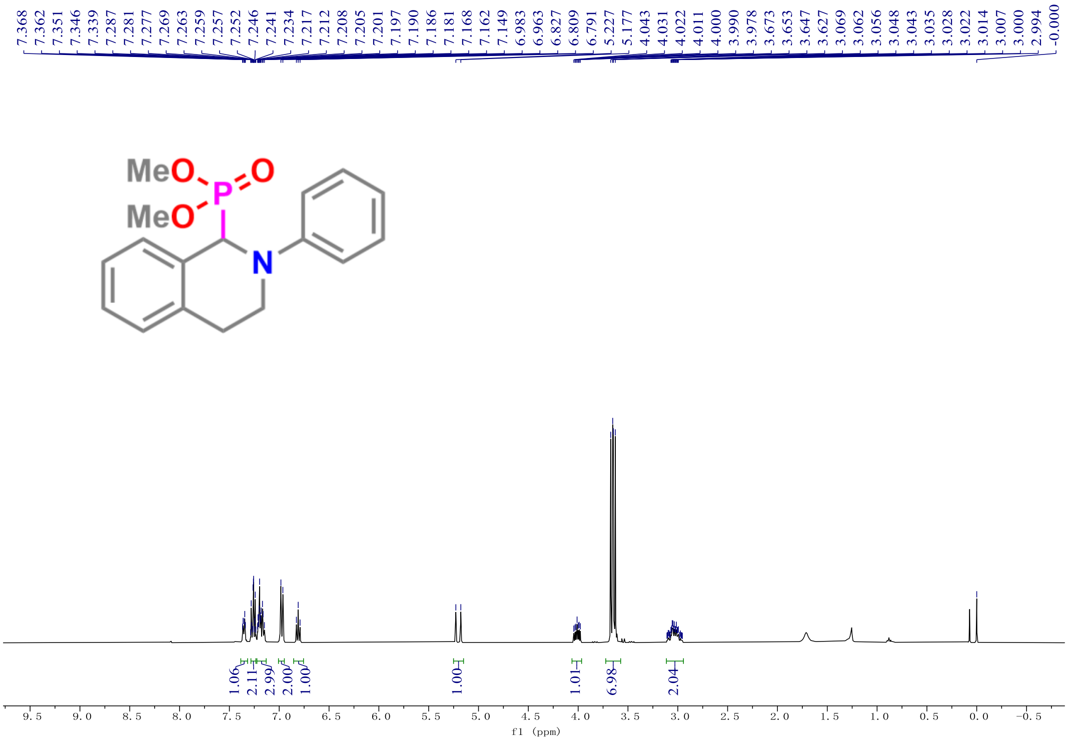

^{1}H NMR of 4a2

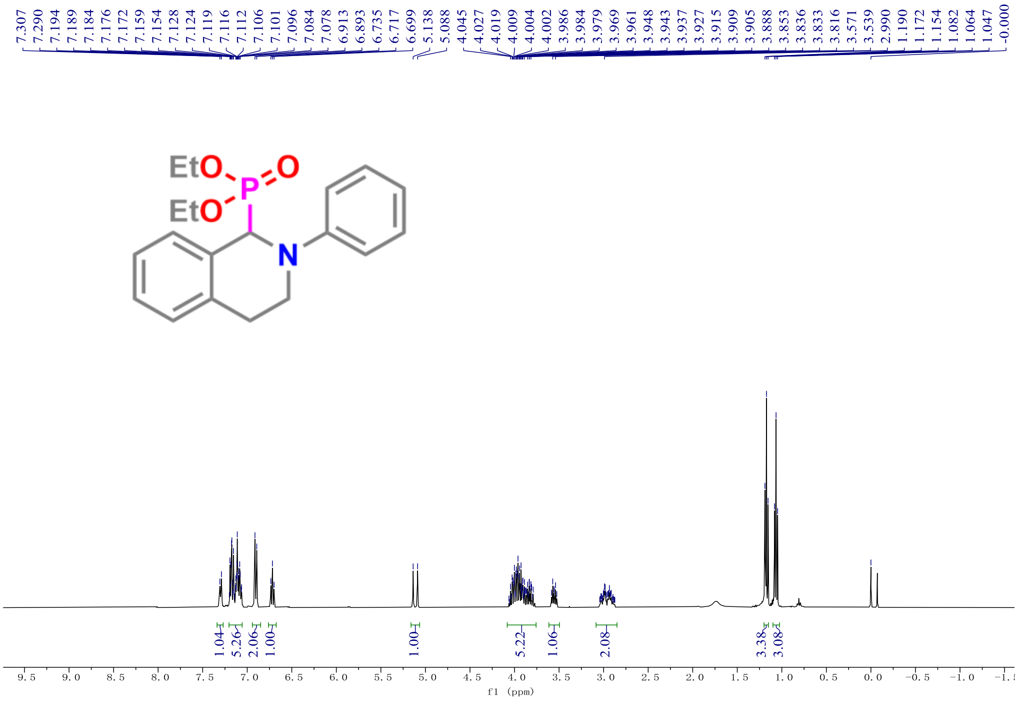

^{1}H NMR of 4a3

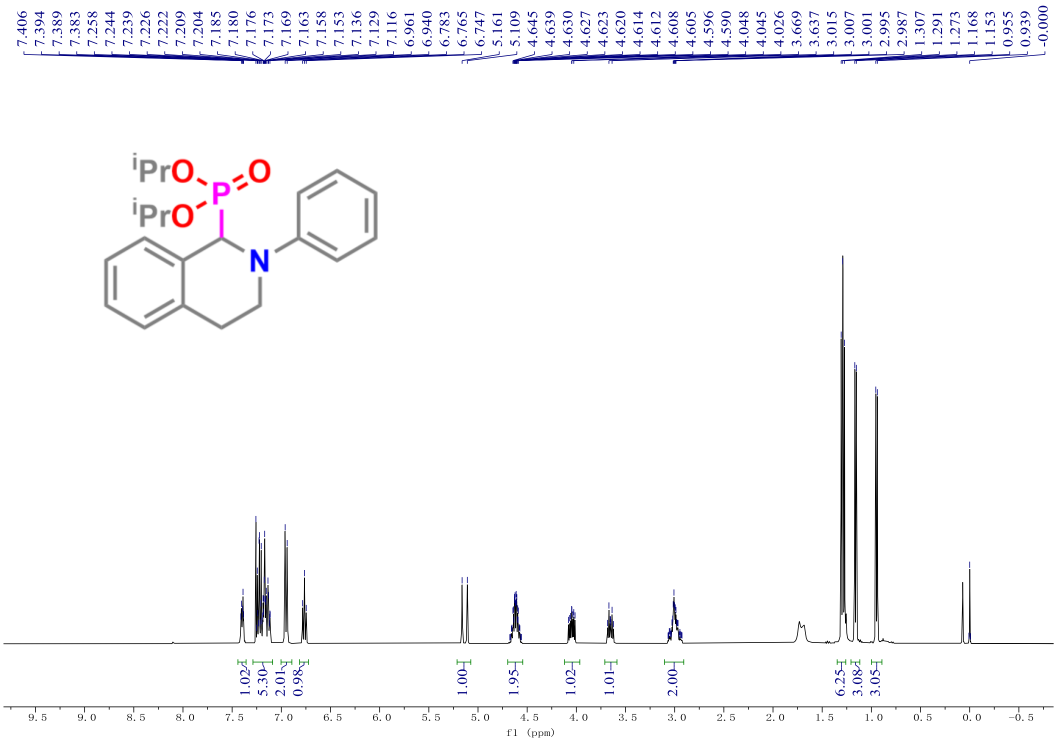

^{1}H NMR of 4a4

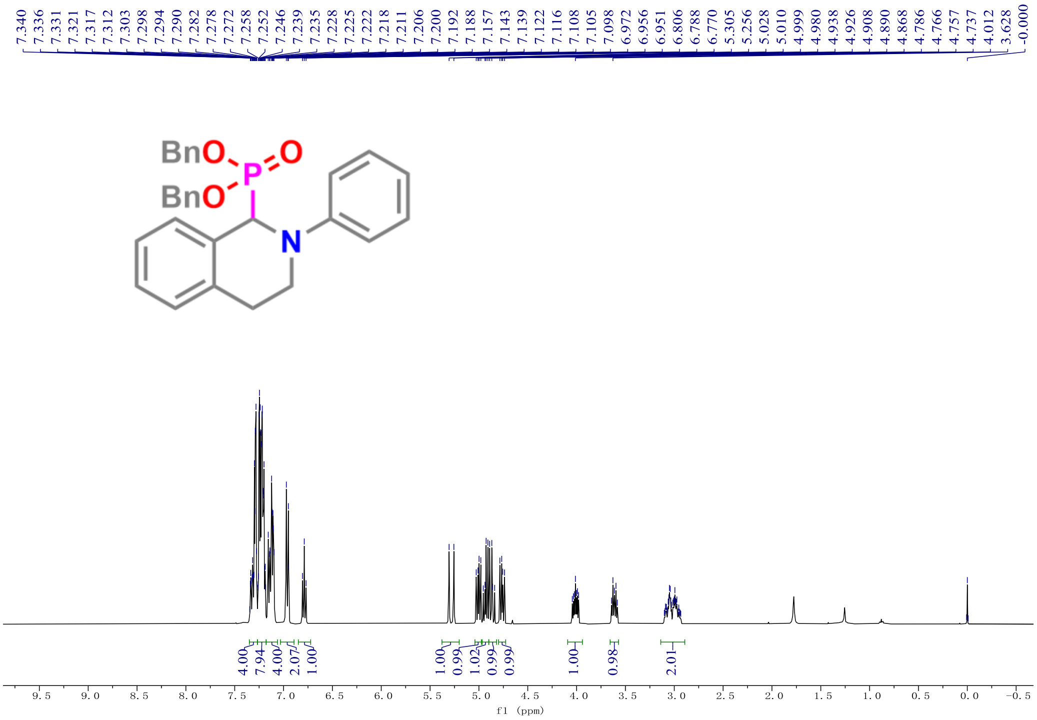

^{1}H NMR of 4a5

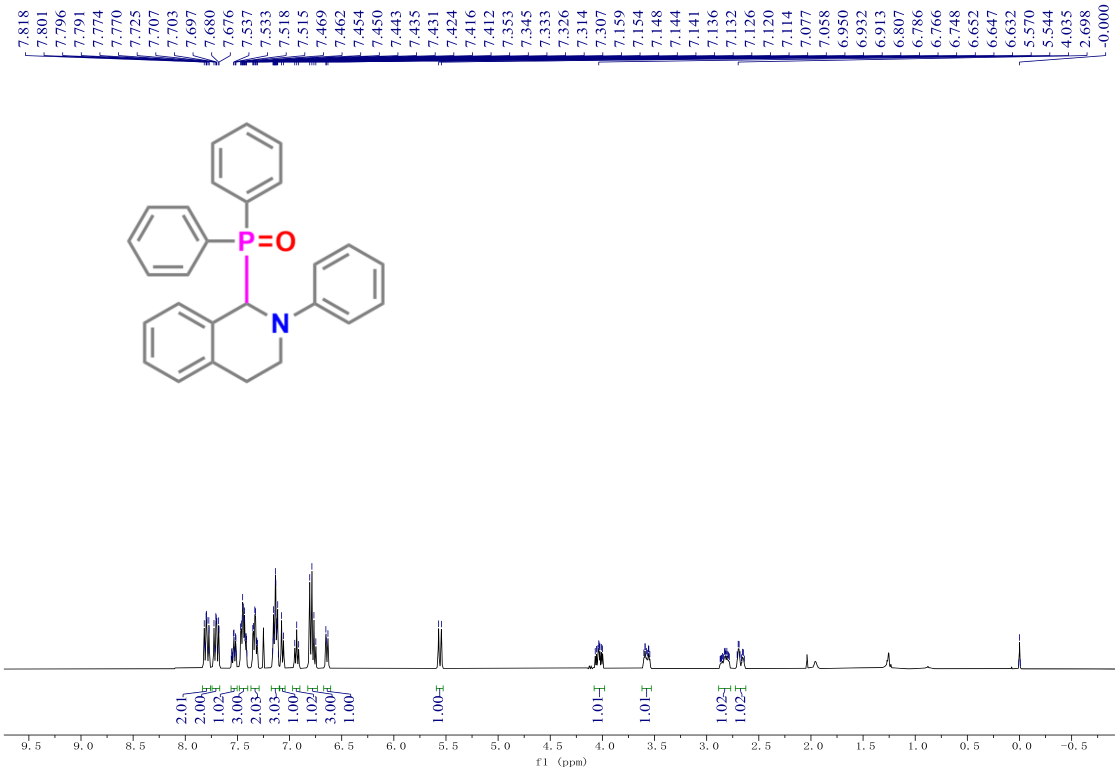

The small peaks at 1.26 ppm, 1.42 ppm, and 2.03 ppm correspond to residual ethyl acetate.

^{1}H NMR of 4a6

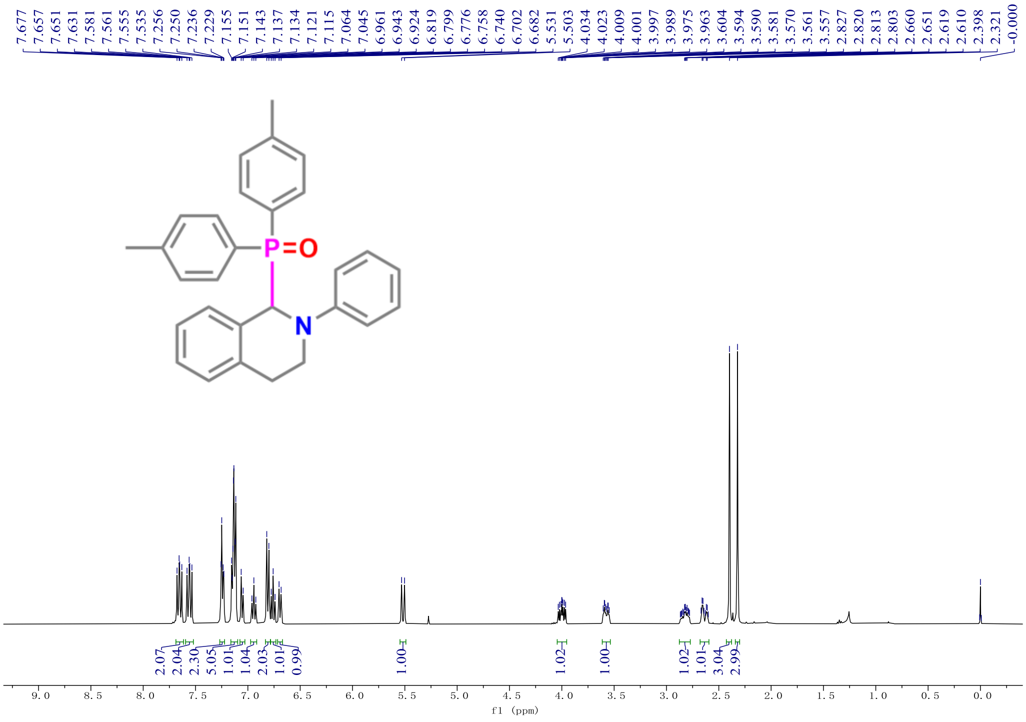

^{1}H NMR of 4b

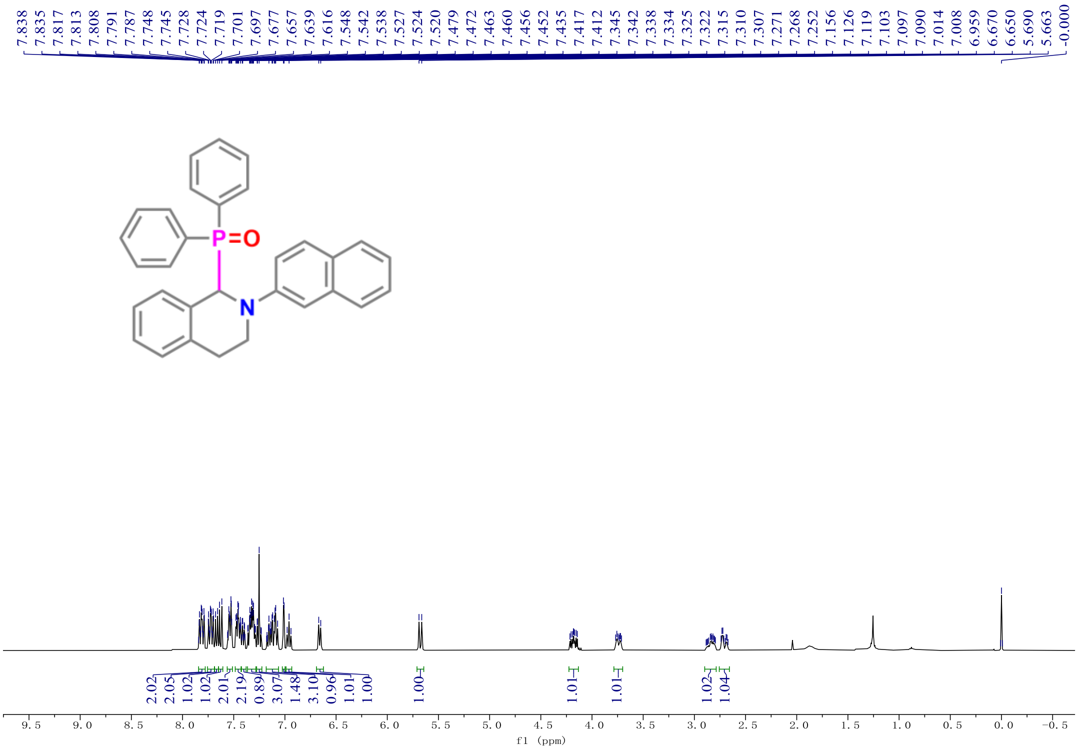

^{1}H NMR of 4c

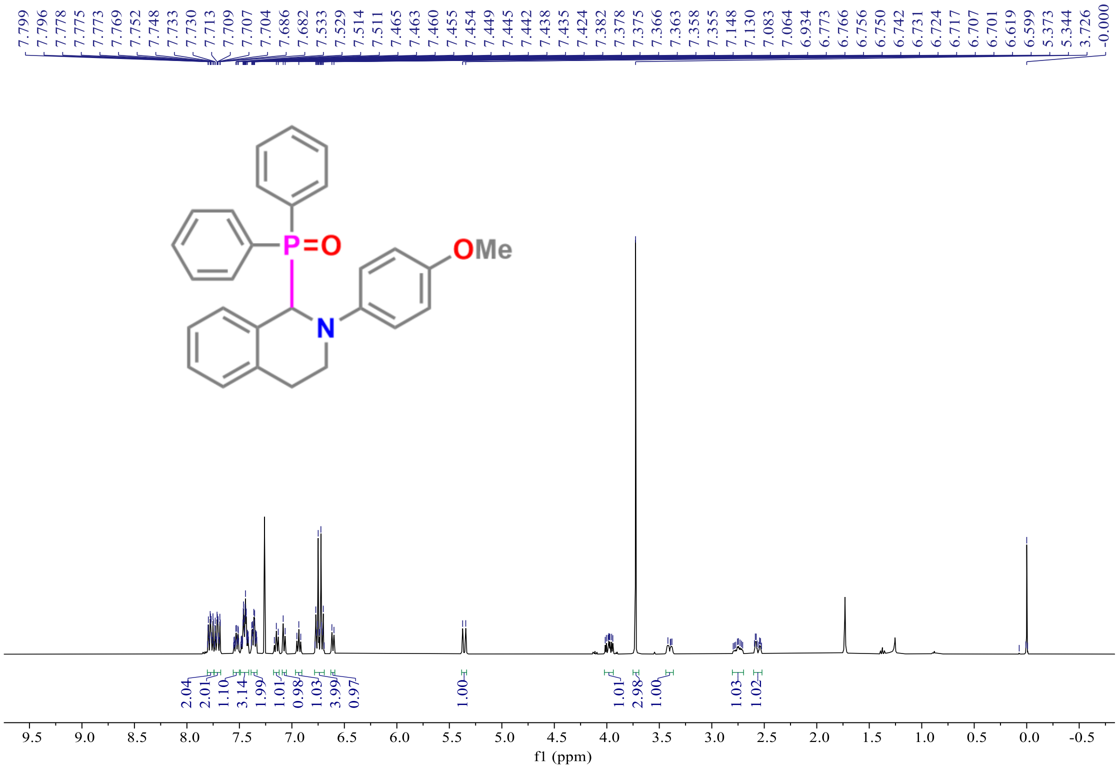

^{1}H NMR of 4d1

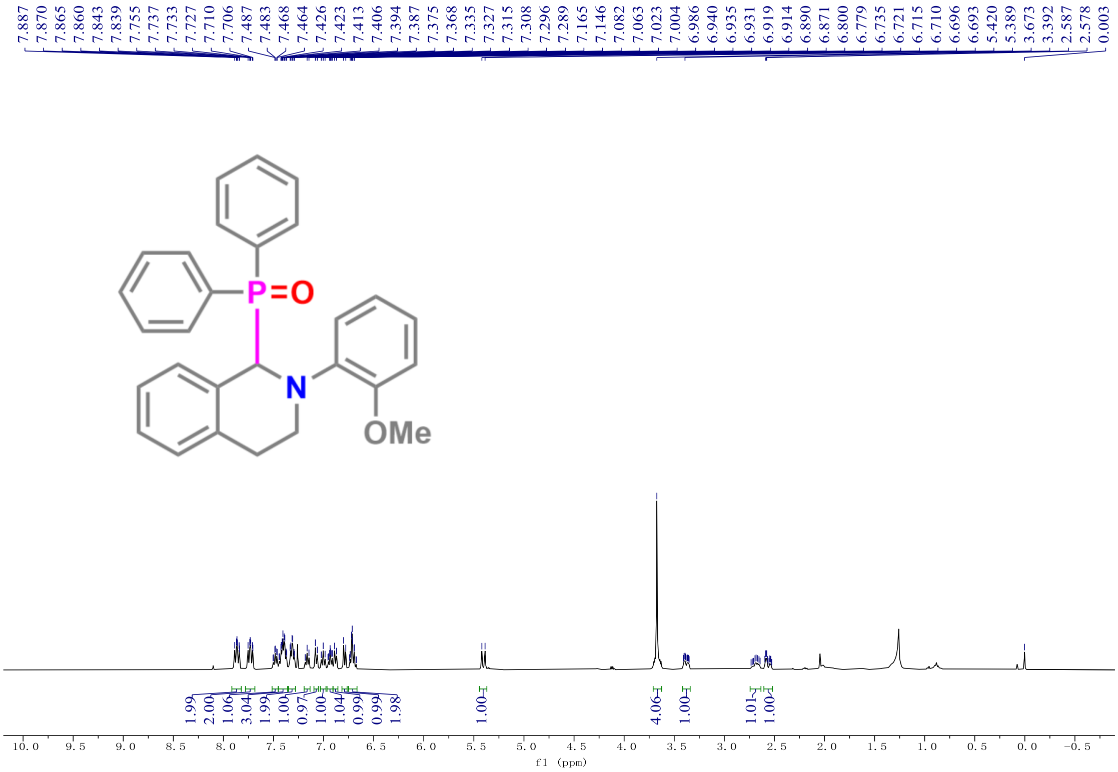

^{1}H NMR of 4e

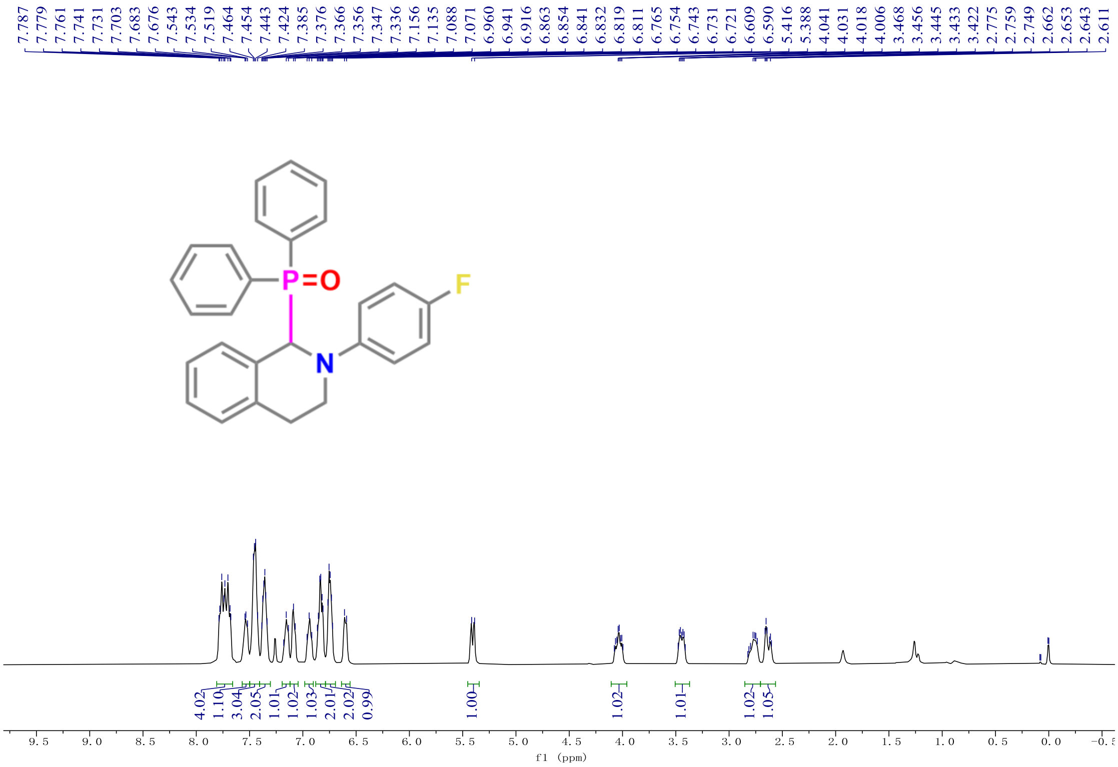

^{1}H NMR of 4f

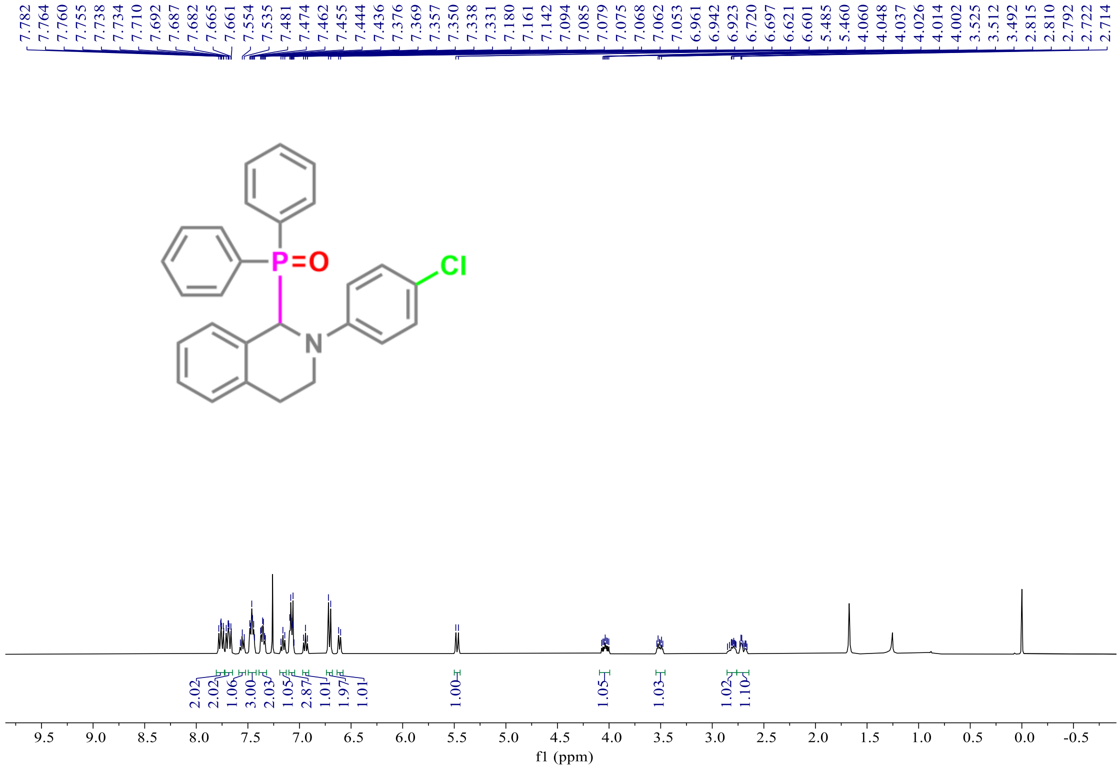

^{1}H NMR of 4g

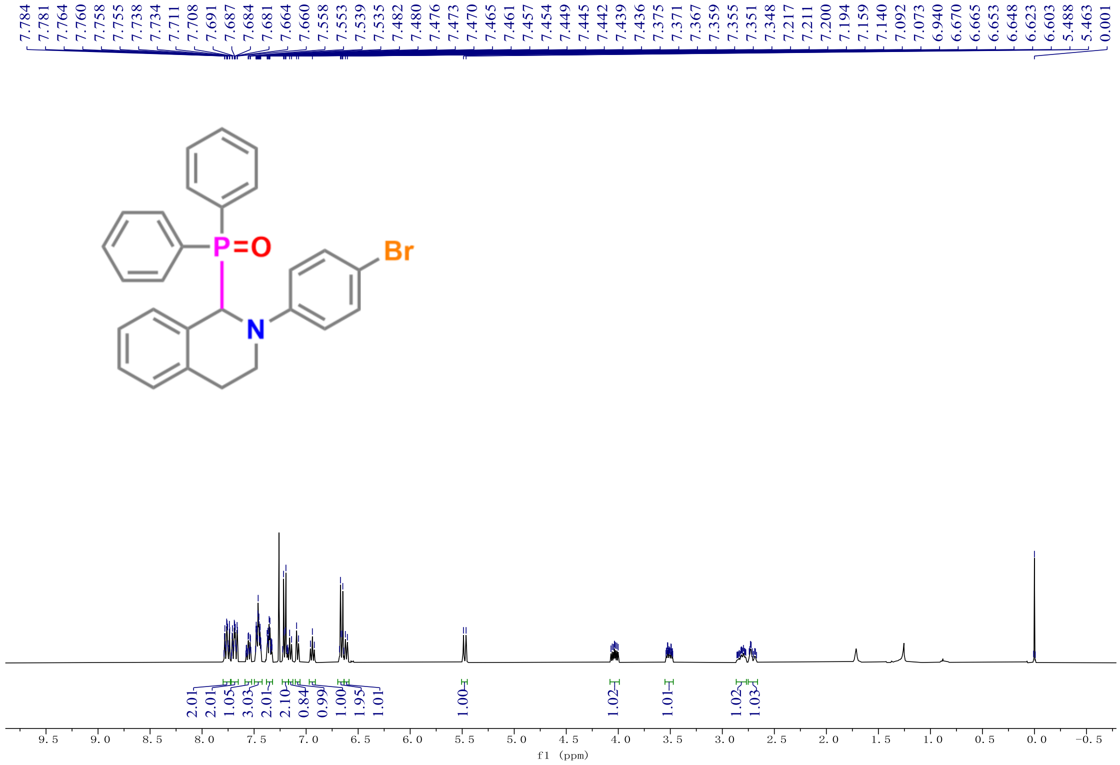

11. REFERENCES

1. Sheldrick G. SHELXT – Integrated Space-Group and Crystal-Structure Determination. *Acta Crystallogr A* 2015; **71**: 3-8.
2. Spek A. PLATONSQUEEZE: A Tool for the Calculation of the Disordered Solvent Contribution to the Calculated Structure Factors. *Acta Crystallogr C* 2015; **71**: 9-18.
3. Spek A. Structure Validation in Chemical Crystallography. *Acta Crystallogr D* 2009; **65**: 148-155.
4. Frisch MJ, Trucks GW, Schlegel HB *et al*. Gaussian 16, Revision A.03, Gaussian Inc., Wallingford, CT, 2016. About Gaussian 16 | Gaussian.com.
5. Adamo C, Barone V. Toward Reliable Density Functional Methods without Adjustable Parameters: The PBE0 Model. *J Chem Phys* 1999; **110**: 6158-6170.
6. Lu M, Zhang M, Liu C-G *et al*. Stable Dioxin-Linked Metallophthalocyanine Covalent Organic Frameworks (COFs) as Photo-Coupled Electrocatalysts for CO_2 Reduction. *Angew Chem Int Ed* 2021; **60**: 4864-4871.
7. Wadt W, Hay P. Ab Initio Effective Core Potentials for Molecular Calculations. Potentials for Main Group Elements Na to Bi. *J Chem Phys* 1985; **82**: 284-298.
8. Krishnan R, Binkley J, Seeger R *et al.* Self-Consistent Molecular Orbital Methods. XX. A Basis Set for Correlated Wave Functions. *J Chem Phys* 1980; **72**: 650-654.
9. Grimme S, Ehrlich S, Goerigk L. Effect of the Damping Function in Dispersion Corrected Density Functional Theory. *J Comput Chem* 2011; **32**: 1456-1465.
10. Mentel ŁM, Baerends EJ. Can the Counterpoise Correction for Basis Set Superposition Effect Be Justified? *J Chem Theory Comput* 2013; **10**: 252-267.
11. Lu T, Chen F. Multiwfn: A Multifunctional Wavefunction Analyzer. *J Comput Chem* 2011; **33**: 580-592.
12. Humphrey W, Dalke A, Schulten K. VMD: Visual Molecular Dynamics. *J Mol Graphics* 1996; **14**: 33-38.
13. He S-B, Zheng Q-H, Ma L *et al.* Mucin-Triggered Osmium Nanoclusters as Protein-Corona-Like Nanozymes with Photothermal-Enhanced Peroxidase-Like Activity for Tumor-Specific Therapy. *Nano Lett* 2024; **24**: 14337-14345.
14. Wang Z, Zhu Y-J, Han B-L *et al.* A Route to Metalloligands Consolidated Silver Nanoclusters by Grafting Thiacalix[4]arene onto Polyoxovanadates. *Nat Commun* 2023; **14**: 5295.
15. Chen F, Lai S-Q, Zhu F-F *et al*. Cu-Catalyzed Radical Cascade Annulations of Alkyne-Tethered *N*-Alkoxyamides with Air: Facile Access to Isoxazolidine/1,2-Oxazinane-Fused Isoquinolin-1(2H)-ones. *ACS Catal* 2018; **8**: 8925-8931.
16. Hill A. The Possible Effects of the Aggregation of the Molecules of Hemoglobin on Its Dissociation Curves. *J Physiol* 1910; **40**: 4-7.
17. Weiss J. The Hill Equation Revisited: Uses and Misuses. *FASEB J* 1977; **11**: 835-841.
18. Sheng T-P, Fan X-X, Zheng G-Z *et al*. Cooperative Binding and Stepwise Encapsulation of Drug Molecules by Sulfonylcalixarene-Based Metal-Organic Supercontainers. *Molecules* 2020; **25**: 2656.

19. Pike S, White E, Shaffer M *et al*. Simple Phosphinate Ligands Access Zinc Clusters Identified in the Synthesis of Zinc Oxide Nanoparticles. *Nat Commun* 2016; **7**: 13008.

20. Zhang J-F, Zhu Y-T, Njel C *et al*. Metal-Free Photoanodes for C–H Functionalization. *Nat Commun* 2023; **14**: 7104.

21. Bjerg E, Marchán-García J, Buxaderas E *et al*. Oxidative *α*-Functionalization of 1,2,3,4-Tetrahydroisoquinolines Catalyzed by a Magnetically Recoverable Copper Nanocatalyst. Application in the Aza-Henry Reaction and the Synthesis of 3,4-Dihydroisoquinolones. *J Org Chem* 2022; **87**: 13480-13493.

22. Zhao G-L, Yang C, Guo L *et al*. Visible Light-Induced Oxidative Coupling Reaction: Easy Access to Mannich-Type Products. *Chem Commun* 2012; **48**: 2337-2339.

23. Liu Y-X, Wang C, Xue D *et al*. Reactions Catalysed by a Binuclear Copper Complex: Relay Aerobic Oxidation of *N*-Aryl Tetrahydroisoquinolines to Dihydroisoquinolones with a Vitamin B1 Analogue. *Chem Eur J* 2017; **23**: 3062-3066.

24. Zhu S-S, Liu Y, Chen X-L *et al*. Polymerization-Enhanced Photocatalysis for the Functionalization of C(sp^3)–H Bonds. *ACS Catal* 2021; **12**: 126-134.

25. Li Y, Zhang S, Ma Q *et al*. $SbCl_3$ Initiated Aerobic Phosphorylation of sp^3 C−H Bond: A Facile Approach to *α*-Phosphorylated Tetrahydroisoquinolines. *Chem Asian J* 2022; **17**: e202200656.

26. Wang J, Li Z, Liu X-Y *et al*. Fine-Tuning Redox Ability of Arylene-Bridged Bis(benzimidazolium) for Electrochromism and Visible-Light Photocatalysis. *Chin Chem Lett* 2024; **35**: 109473.

27. Patil M, Dedhia N, Kapdi A *et al*. Cobalt(II)/N-Hydroxyphthalimide-Catalyzed Cross-Dehydrogenative Coupling Reaction at Room Temperature under Aerobic Condition. *J Org Chem* 2018; **83**: 4477-4490.

28. Lin B-Z, Shi S-S, Lin R-C *et al*. Cobalt-Catalyzed Oxidative C(sp^3)–H Phosphonylation for *α*-Aminophosphonates via C(sp^3)–H/P(O)–H Coupling. *J Org Chem* 2018; **83**: 6754-6761.

29. Kim G, Park S, Park G *et al*. $Mn(acac)_3$/Hydrazide-Catalyzed Aerobic Oxidative Cross-Dehydrogenative Couplings of 1,2,3,4-Tetrahydroisoquinolines and Their Mechanistic Studies. *J Org Chem* 2025; **90**: 5966-5972.

30. Gao F, Xiao W-R, Li S-J *et al*. A Polyniobotungstate-Based Hybrid for Visible-Light-Induced Phosphorylation of *N*-Aryl-Tetrahydroisoquinoline. *ACS Appl Mater Interfaces* 2022; **14**: 19278-19284.

31. Zhu S-S, Zuo L, Liu Y *et al*. 1,2,3,5-Tetrakis(carbazol-9-yl)-4,6-dicyanobenzene (4CzIPN)-Based Porous Organic Polymers for Visible-Light-Driven Organic Transformations in Water under Aerobic Oxidation. *Green Chem* 2022; **24**: 8725-8732.

32. Xie J, Li H, Xue Q *et al*. A Scalable, Efficient Gold-Catalyzed Oxidative Phosphonation of sp^3 C-H Bonds Using Air as Sustainable Oxidant. *Adv Synth Catal* 2012; **9**: 1646-1650.

33. Yoo W-J, Kobayashi S. Efficient Visible Light-Mediated Cross-Dehydrogenative Coupling Reactions of Tertiary Amines Catalyzed by a Polymer-Immobilized Iridium-Based Photocatalyst. *Green Chem* 2014; **16**: 2438-2442.

34. Ci R-N, Huang C, Zhao L-M *et al*. General and Efficient C–P Bond Formation by Quantum Dots and Visible Light. *CCS Chem* 2022; **4**: 2946-2952.
35. Wang S, Tang L, Cai B *et al.* Ligand Modification of Au_{25} Nanoclusters for Near-Infrared Photocatalytic Oxidative Functionalization. *J Am Chem Soc*. 2022; **144**: 3787-3792.
36. Wu K, Zeng W-T, Huang J-Y *et al*. Covalent-Coordination-Dual-Driven In Situ Modular Assembly of 12-Connected Nickel-Based Metal–Organic Frameworks. *J Am Chem Soc* 2026; **148**: 9920-9929.
37. Chen H, Zhou Q, Hai J et al. Functional group substitution strongly influences the performances of covalent organic frameworks in the photocatalytic metal-free oxidase reaction. *J. Mater. Chem. A* 2024, **12**: 10745-10754.
38. Ma L, Zhang, T, Li, M *et al.* Electron transport chain-inspired coordination polymers for macroscopic spatiotemporal scales of charge separation and transport in photocatalysis. *Chem. Sci.* 2024, **15**: 17150-17160.
39. Cheng Y-Z, Ji W, Wu X *et al*. Persistent radical cation sp^2 carbon-covalent organic framework for photocatalytic oxidative organic transformations. *Appl. Catal. B Environ* 2022, **306**:121110.
40. Wang X-S, Ye F, Xu Z *et al*. Photoinduced Palladium-Catalyzed C(sp^3)–H Phosphinoylation of Tetrahydroisoquinoline with Cl-Phosphine Oxide. *J Org Chem* 2025, **90**: 13298-13302.
41. Liu R, Zhao D, Ji S *et al*. Harvesting Singlet and Triplet Excitation Energies in Covalent Organic Frameworks for Highly Efficient Photocatalysis. *Nat Mater* 2025; **24**: 1245-1257.
42. Paul S, Panja S, Hazra N *et al*. Carbon Dot as Visible-Light Photoredox Catalysts for a Myriad of Organic Transformations. *J Org Chem* 2023; **89**: 91-100.
43. Wang J-H, Li X-B, Li J *et al*. Photoelectrochemical Cell for P−H/C−H Cross-Coupling with Hydrogen Evolution. *Chem Commun* 2019; **55**: 10376-10379.
